# Comprehensive Review of Models and Methods for Inferences in Bio-Chemical Reaction Networks

**DOI:** 10.3389/fgene.2019.00549

**Published:** 2019-06-14

**Authors:** Pavel Loskot, Komlan Atitey, Lyudmila Mihaylova

**Affiliations:** ^1^College of Engineering, Swansea University, Swansea, United Kingdom; ^2^Department of Automatic Control and Systems Engineering, University of Sheffield, Sheffield, United Kingdom

**Keywords:** automation, Bayesian analysis, biochemical reaction network, estimation, inference, modeling, survey, text mining

## Abstract

The key processes in biological and chemical systems are described by networks of chemical reactions. From molecular biology to biotechnology applications, computational models of reaction networks are used extensively to elucidate their non-linear dynamics. The model dynamics are crucially dependent on the parameter values which are often estimated from observations. Over the past decade, the interest in parameter and state estimation in models of (bio-) chemical reaction networks (BRNs) grew considerably. The related inference problems are also encountered in many other tasks including model calibration, discrimination, identifiability, and checking, and optimum experiment design, sensitivity analysis, and bifurcation analysis. The aim of this review paper is to examine the developments in literature to understand what BRN models are commonly used, and for what inference tasks and inference methods. The initial collection of about 700 documents concerning estimation problems in BRNs excluding books and textbooks in computational biology and chemistry were screened to select over 270 research papers and 20 graduate research theses. The paper selection was facilitated by text mining scripts to automate the search for relevant keywords and terms. The outcomes are presented in tables revealing the levels of interest in different inference tasks and methods for given models in the literature as well as the research trends are uncovered. Our findings indicate that many combinations of models, tasks and methods are still relatively unexplored, and there are many new research opportunities to explore combinations that have not been considered—perhaps for good reasons. The most common models of BRNs in literature involve differential equations, Markov processes, mass action kinetics, and state space representations whereas the most common tasks are the parameter inference and model identification. The most common methods in literature are Bayesian analysis, Monte Carlo sampling strategies, and model fitting to data using evolutionary algorithms. The new research problems which cannot be directly deduced from the text mining data are also discussed.

## 1. Introduction

Biological systems are presently subject to extensive research efforts to ultimately control the underlying biological processes. The challenge is the level of complexity of these systems with intricate dependencies on the internal and external conditions. Biological systems are inherently non-linear, dynamic as well as stochastic. Their responses to input perturbations are often difficult to predict as they may respond differently to the same inputs. Moreover, biological phenomena must be considered at different spatio-temporal scales, from single molecules to gene-scale reaction networks.

Many biological systems can be conveniently represented as biological circuits (Zamora-Sillero et al., 2011), or as networks of biochemical reactions (Ashyraliyev et al., 2009). Common examples of biological systems which can be described as BRNs are: metabolic networks, signal transduction networks, gene regulatory networks (GRNs), and more generally, the networks of biochemical pathways. Moreover, BRNs share similar characteristics with evolutionary and prey-predatory networks in population biology, and disease spreading networks in epidemiology. Synthetic bio-reactors and other types of chemical reactors used in industrial production are other examples of BRNs (Ali et al., 2015).

Qualitative as well as quantitative observations of biological systems are necessary to elucidate their functional and structural properties. Despite the advent of high throughput experiments, the biological phenomena are often only partially observed. Since the internal system state cannot be fully nor directly observed, it must be inferred from the measurements. Such inferences are possible due to the dependency of observations on the internal states and parameter values (Fröhlich et al., 2017). Single molecule techniques are promising for advancing the cell biology as they enable more focused observations, however, their resolution and dimensionality is still limited.

The observations in experiments are often distorted and noisy, and involve some form of averaging. Extended models can be assumed for the measurements involving distortion (Ruttor and Opper, 2009). The measurement noise may not be additive nor Gaussian, and its variance may be dependent on the values of other parameters. The parameter values may differ for *in vitro* and *in vivo* experiments (Famili et al., 2005). In systems comprising chemical reactions, the parameters of interest are usually initial and instantaneous concentrations, reaction rates and possibly other kinetic constants including the diffusion and drift coefficients. The molecular concentrations can be usually measured directly whereas the other parameters must be inferred from measurements (Fröhlich et al., 2017). The parameter inferences as well as measurements can be performed sequentially (online) or in batches (off-line) (Arnold et al., 2014).

In BRNs, the number of chemical species is usually much smaller than the number of chemical reactions. In some cases, it may be useful to estimate the number of reactions between consecutive measurements (Reinker et al., 2006). The structural identifiability of a chemical reaction system is affected by which reactions are occurring.

The observations at possibly non-equidistant time instances represent longitudinal data which can be used to create or validate mathematical models. The rate of discrete time observations is important (Fearnhead et al., 2014), since more frequent observations can be costly, and affect the observed biological processes. Processing the large volumes of data is also computationally demanding. The observations and their processing can be merged to create so-called observers in order to replace the high-cost sensors in chemical reactors (Rapaport and Dochain, 2005). Observers can be classified as explanatory or predictive to describe the existing or future data, respectively (Ali et al., 2015). Observers can process discretized and delayed measurements, and yield the interval measurements of quantities with a variable observation gain (Vargas et al., 2014). The average state observers of large-scale systems are defined in Sadamoto et al. (2017).

The dynamics of biological processes can be elucidated from their mathematical models. The importance of modeling in biology is discussed in Chevaliera and Samadb (2011), and general modeling strategies are described in Banga and Canto (2008). The research problems in biology dictate what physical and chemical processes must be included in the models. It is usually more efficient to only collect the observations which are necessary to formulate and test a biological hypothesis than to perform extensive, time consuming and expensive laboratory experiments. Such a strategy is referred to as a forward modeling (Reinker et al., 2006). On the other hand, finding the parameter values to reproduce the observations can be enhanced by the experiment design, and it is known as a reverse modeling (Hagen et al., 2013). The differences between forward and reverse modeling strategies are explained in Ashyraliyev et al. (2009).

The models of biological systems are dependent on the *in vivo* or *in vitro* experiments considered. BRNs can be modeled as deterministic input-output non-linear transformations which can be sometimes locally linearized at a given time scale and resolution. The models can be modified using additional transformations to facilitate their analysis. Apart from deterministic models, there are also stochastic, event-driven and probabilistic models of BRNs. When the number of species is large, the stochastic models converge to deterministic models (Rempala, 2012). The same model used multiple times can represent a biological population (Woodcock et al., 2011).

Biological models need to be unbiased in order to avoid systematic errors. Since they are usually evaluated many times, they need to be computationally fast, and at the right level of coarse grain description. For instance, microscopic stochastic models may be computationally expensive whereas a deterministic macroscopic description, such as population-average modeling may not be sufficiently accurate due to a low level of resolution.

Development of large-scale kinetic systems is one of the key tasks in contemporary computational biology (Penas et al., 2017). The corresponding models can be multidimensional and have 100's or even 1000's of parameters, and constraints while the initial conditions are not known. The models can be hierarchical or nested, and have parts interconnected by multiple feedback loops (Rodriguez-Fernandez et al., 2013). The parameter estimation for large-scale reaction networks is considered in Remlia et al. (2017).

The model analysis can yield the transient responses of a biological system, and to obtain the behavior at steady state or in equilibrium (Atitey et al., 2018a). It may be also useful to explore complex multi-dimensional parameter spaces. The viable parameter values of many models of biological systems form only a small fraction of the overall parameter space (Atitey et al., 2019), so identifying this sub-volume by simple sampling is rather inefficient (Zamora-Sillero et al., 2011). The model analysis is further complicated by the size of the state space, the number of unknown parameters, the analytical intractability, and various numerical problems. Evaluation of the observation errors can both facilitate as well as validate the model analysis (Bouraoui et al., 2015).

The majority of analytical and numerical methods can be used universally for models with different structures. The efficiency of model analysis can be considered in the statistical or computational sense. In the statistical sense, the analysis needs to be robust against the uncertainty in model structure and the parameter values estimated from noisy and limited observations. The computational efficiency can be achieved by developing the algorithms which are prone to massively parallel implementations (Nobile et al., 2012).

In this review paper, we are primarily concerned with the parameter inference in biological and chemical systems described by various models of BRNs. We use the terms inference and estimation interchangeably. In the literature, the parameter inference is also referred to as an inverse problem (Engl et al., 2009), point estimation, model calibration and model identification. The key objective of the parameter inference is to minimize a suitably defined estimation error while suppressing the effects of measurement errors (Sadamoto et al., 2017). More recently, machine learning methods are becoming popular as an alternative to learn not only the model parameters, but also to learn the model features from the labeled and unlabeled observations (Sun et al., 2012; Schnoerr et al., 2017).

The parameter inference is affected by many factors. For instance, different models experience a different degree of structural identifiability. Provided that different parameter values or different inputs generate the same dynamic response, such as the statistics of synthesized molecules, the model parameters cannot be identified, or can only be partially identified. In some cases, the structural identifiability can be overcome by changing the modeling strategy (Yenkie et al., 2016). The structural identifiability is a necessary but not sufficient condition for the overall model identifiability (Gábor et al., 2017). A relationship between the identifiability and observability is discussed in Baker et al. (2011). The practical identifiability (also known as a posterior identifiability) assesses whether there is enough data to suppress the measurement noises. It may be beneficial to test the identifiability of the parameters of interest prior to attempting their inference. For instance, the parameters may not be identifiable at a given time scale, or the data may not have sufficient dimensionality (variability) or volume. The lack of suitable data makes the inference problem to be ill conditioned. A crucial issue is then how well the parameters need to be known in order to answer a given biological question. However, in all cases, it is important to validate the obtained estimates.

Sensitivity analysis can complement as well as support the parameter estimation (Saltelli et al., 2004; Fröhlich et al., 2016). In particular, the parameters can be ranked in the order of their importance, from the most easy to the most difficult to estimate. The parameters can be screened using a small amount of observations to select those which are identifiable prior to their inference from a full set of data. Other tasks in sensitivity analysis include prioritizing the parameters, testing their independence, and fixing or identifying the important regions of their values. A survey of methods used for the sensitivity analysis in BRNs is provided in Saltelli et al. (2005). The sensitivity profiles of 180 biological models were compared and analyzed in Erguler and Stumpf (2011).

In the rest of this paper, our main objective is to survey the models and methods which have been used in the literature to perform the parameter and state inferences in BRNs. After explaining our methodology in Section 2, different modeling strategies for BRNs are outlined in Section 3. It is followed by a survey of the estimation methods for BRNs and the related computational tasks in Section 4. Since the performance and effectiveness of estimation methods is crucially dependent on the specific models adopted, in Section 5, we explore what methods are used in literature for given models, and also, what estimation methods are used in given tasks. This enables us to uncover the possible future research directions in sub-section 5.1. We also mention several inference techniques which are used in other fields, but which can likely be assumed for BRNs.

Our contributions are 3-fold, and they are structured as the following surveys:

1. Models and modeling strategies of BRNs;
2. Parameter estimation methods, strategies and related tasks for models of BRNs;
3. Combinations of models and parameter estimation methods and tasks for BRNs.

The first version of this review appeared online as Loskot et al. (2019).

## 2. Methodology

It is important to define first the scope of our comprehensive review in order to understand its aims and constraints. In particular, there are at least 14 types of literature reviews which differ in their purpose, methodology, and limitations (Grant and Booth, 2009). For example, the literature review can be systematic (SLR) to a various degree (Tranfield et al., 2003). The purpose of SLR is to answer an *a priori* formulated question or hypothesis using a clearly defined procedure of searching and examining the literature, so that it can be reproduced by others. The SLRs are particularly suitable for the evidence (data) based research fields as in biology and medicine (Grant and Booth, 2009).

However, the main purpose of our review is to present a comprehensive and critical overview of the models and methods which have been popular in literature to perform different inference tasks in BRNs. Such a review is known as the traditional or narrative literature review (NLR) (Onwuegbuzie and Frels, 2016). The outcome of NLR is state-of-the art of current knowledge, and identifying knowledge gaps, patterns, and emerging trends which can guide future research. The present review is comprehensive in the sense of striving to collect and categorize as many models and methods for inferences in BRNs as possible in order to provide a reference for further research on this topic. It leaves out the requirement for the review to be systematic and reproducible. We also cannot guarantee that all important and relevant papers in the field were identified or considered.

Our review resumed by collecting a relatively large number of representative and otherwise relevant papers. The papers were first identified using various keyword searches in Google. The subsequent more refined searches were performed in Google Scholar which also provides information on the citing papers, and contains the collections of papers by individual authors. Our intention was to specifically consider the papers on inference problems in BRNs; there are many other papers which are concerned with methods and strategies for general dynamic systems. We have also considered a number of graduate research theses which are publicly accessible online. The theses were evaluated separately from the papers. Moreover, we decided to exclude electronic books and textbooks from our study as their coverage is normally rather broad, and their contents processing would require to identify and extract chapters into separate files.

Almost 700 electronic documents in the portable document format (PDF) were collected from various sources using the following search keywords and their combinations: *biochemical, network, model, inference, estimation, parameters*, and *identification*. The initially collected papers were manually evaluated whether they are sufficiently relevant to the purpose of our study. For example, many papers involving parameter estimation in general dynamic systems were discarded unless they were deemed to have some other value for our review. While evaluating the papers, we were updating 2 lists of keywords. The first list contains keywords representing the models of BRNs, such as *state-space, differential equation, Markov chain*, and similar. The second keyword list describes the inference methods, for example, *Bayesian, MCMC, least squares*, and other. The keywords were used to perform more focused searches for additional papers, and to screen and classify the already collected papers. In the end, we assumed 25 BRN models and 23 inference methods, and also defined the 5 inference-related tasks: *estimation, inference, identifiability, observability, reachability, experiment design, bifurcation analysis*, and *sensitivity analysis*.

**Figure 1:**
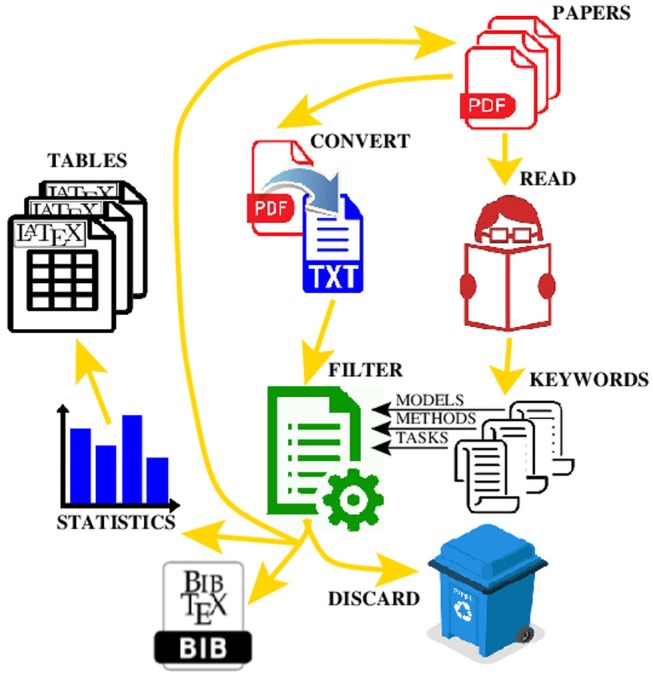
A workflow for processing the PDF files to automate the production of the 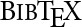 reference file, and the 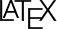 tables with statistical data.

All PDF documents were converted into ordinary text files to enable text mining of their contents. The text files were scanned to find occurrences of the keywords from the 2 lists defined above using the regular expressions representing textual patterns. The papers containing sufficiently large number of keywords were kept whereas the papers that did not pass the test were manually checked before being discarded. It allowed us to quickly reduce the number of papers from 700 to <300. There is a trade-off between the strictness (i.e., reliability) of the automated paper selection and possibility to automatically discard some papers, and how many of the remaining papers have to be checked manually. We observed that a small number of occurrences of a keyword usually indicates that the keyword appeared mainly within the references of the paper. A high-level view of our paper selection process is depicted in Figure 1.

As the number of published papers is increasing exponentially, there is clearly a need to develop new tools to facilitate more automated paper selection and pre-screening (Loskot, 2018). In order to automate many text processing tasks and enable evaluation of the 100's of papers in our study, we took advantage of the text processing capabilities readily available on the Linux operating systems. In particular, all PDF files were first converted to ordinary text files with the ascii encoding of characters (UTF-8) and the transliterated special characters in the foreign alphabets. The conversion was done using the standard pdftotext utility version 0.62 which is based on the open source Poppler library developed for rendering the PDF files. The PDF conversion is not and does not have to be 100% accurate. For example, the words containing characters which are not recognized can be omitted. Moreover, some words are occasionally split into several parts which can be detected using a dictionary. However, such undesirable cases can be largely neglected for our purposes. It is also useful to remove the end-of-line characters from within the paragraphs, and to merge parts of the paragraphs which were split by displayed equations or by page breaks in order to improve the searches for more complex text patterns.

The scripts to automate many text processing tasks were programmed in the BASH interpreter version 4.4 running in a Linux terminal. The scripts use extensively standard Linux tools including grep, sed, and awk programmable text filters. In particular, the scripts were used to automatically identify and count relevant papers, generate LaTeX tables to visualize the results, facilitate semi-automated creation of bibliographic entries in the master BibTeX file, and to obtain URL links for citing papers in Google Scholar (Table S3). The keyword searches can assume multiple terms combined in sophisticated hierarchical expressions with AND-OR operators, include conditions on the number of occurrences, and sort the results as required.

However, the adopted procedure and the tools we developed for identifying and selecting the most relevant papers have some limitations. In particular, the paper selection and text mining in our study is restricted to keyword searches using regular expressions. A certain level of manual processing is still required, although it is likely that this can be reduced with future versions of the tools. A fully automated paper analysis with minimum human interventions would require the use of natural language processing (NLP). The NLP libraries are already available in many programming languages, but it is outside the scope of the present paper.

Furthermore, our study is mostly concerned with inferences of parameters and states whereas the inferences of network structures (i.e., which chemical reactions are occurring) is omitted. Our classification of models and methods have been developed to facilitate the analysis of trends and patterns in the literature. For instance, some models and methods considered in the next sections may be related, or a special case of one another. However, for the purpose of our study, the models and methods are presented as they appear in the cited references. In addition, although we generally distinguish between the deterministic and stochastic inference methods, we do not make such strict distinction between the deterministic and stochastic models. It should be also noted that many references can be cited in multiple contexts, i.e., for several models or methods considered. In many cases, the papers are chosen as illustrative examples for a given model or method, so they are likely many other important references which could be cited. Finally, more complete information how the papers cited in this review are related to the assumed models and methods is given in the Supplementary Tables.

## 3. Review of Modeling Strategies for BRNs

Mathematical models describe dependencies of observations on the model parameters. A general procedure for constructing mathematical models of biological systems is described in Chou and Voit (2009). The bio-reactors are mathematically described in Vargas et al. (2014), Ali et al. (2015), and Farza et al. (2016). The model building is an iterative process which is often combined with the optimum experiment design (Rodriguez-Fernandez et al., 2006b). The model structure affects the selection as well as the performance of parameter estimators. The structural identifiability and validity of multiple models together with the parameter sensitivity was considered in Jaqaman and Danuser (2006). The parameter estimation can be performed together with the discrimination among several competing models, for instance, when the model structure is only partially known. The model structure and the parameter values to achieve the desired dynamics can be obtained by the means of statistical inference (Barnes et al., 2011). The synthesis of parameter values for BRNs is also considered in Češka et al. (2017). The probabilistic model checking can be used to facilitate the robustness analysis of stochastic biochemical models (Česka et al., 2014). The model checking is investigated in a number of references including Palmisano (2010), Brim et al. (2013), Česka et al. (2014), Mizera et al. (2014), Hussain et al. (2015), Mancini et al. (2015), Češka et al. (2017), and Milios et al. (2018). An iterative, feedback dependent modularization of models with the parameters identification was devised in Lang and Stelling (2016). A selection among several hierarchical models assuming Akaike information was studied in Rodriguez-Fernandez et al. (2013).

Modeling strategies of BRNs often involve the kinetics of chemical reactants which are described by the law of mass action or by the rate law (Schnoerr et al., 2017). Both these laws model the dependency of chemical reaction rates on the species concentrations. The reaction kinetics can be considered at steady state or in the transition to steady state, although the steady state may not be always achieved. There are also other kinetic models, such as the Michaelis-Menten kinetics for the enzyme-substrate reactions (Rumschinski et al., 2010), the Hill kinetics for cooperative ligand binding to macromolecules (Fey and Bullinger, 2010), the kinetics for logistic growth models in GRNs (Ghusinga et al., 2017), the kinetics for the birth-death processes (Daigle et al., 2012), and the stochastic Lotka-Volterra kinetics which are associated with the prey-predatory networks (Boys et al., 2008).

Single molecule stochastic models describe BRNs qualitatively by generating the probabilistic trajectories of species counts. A BRN can be modeled as a sequence of reactions occurring at random time instances (Amrein and Künsch, 2012). The stochastic kinetics mathematically correspond to a Markov jump process with the random state transitions between the species counts (Andreychenko et al., 2012). Alternatively, the time sequence of chemical reactions can be viewed as a hidden Markov process (Reinker et al., 2006). The Markov jump processes can be simulated exactly using the classical Gillespie algorithm, so that the competing reactions are selected assuming a Poisson process with the intensity proportional to the species counts (Golightly et al., 2012; Kügler, 2012), although, in general, the intensity can be an arbitrary function of the species counts. The random occurrences of reactions can be also described using the hazard function (Boys et al., 2008). Non-homogeneous Poisson processes can be simulated by the thinning algorithm of Lewis and Shedler (Sherlock et al., 2014).

The number of species in BRN and their molecule counts can be large, so the state space of the corresponding continuous time Markov chain (CTMC) model is huge (Angius and Horváth, 2011). The large state space can be truncated by considering only the states significantly contributing to the parameter likelihood (Singh and Hahn, 2005). The parameter likelihoods can be updated iteratively assuming the increments and decrements of the species counts (Lecca et al., 2009). The probabilistic state space representations of BRNs as dynamic systems were considered in Andreychenko et al. (2011), Gupta and Rawlings (2014), McGoff et al. (2015), and Schnoerr et al. (2017). An augmented state space representation of BRN derived from the ordinary differential equations (ODEs) is obtained in Baker et al. (2013).

More generally, mechanistic models of BRNs are obtained by assuming that biological systems are built up from the actual or perceived components which are governed by the physical laws (Hasenauer, 2013; Pullen and Morris, 2014; White et al., 2016; Fröhlich et al., 2017). It is a different strategy to empirical models which are reverse-engineered from observations (Geffen et al., 2008; Bronstein et al., 2015; Dattner, 2015). The black-box modeling can be assumed with some limitations when there is little knowledge about the underlying biological processes (Chou and Voit, 2009).

Many models containing multiple unknown parameters are often poorly constrained. Even though such models may be still fully identifiable, they are usually ill-conditioned, and often referred to as being sloppy (Toni and Stumpf, 2010; Erguler and Stumpf, 2011; White et al., 2016). The parameter estimation and experimental design for sloppy models are investigated in Mannakee et al. (2016) where it is shown that the dynamic properties of sloppy models usually depend only on several key parameters with the remaining parameters being largely unimportant. A sequence of hierarchical models with increasing complexity was proposed in White et al. (2016) to overcome the complexity and sloppiness of conventional models.

### 3.1. Modeling BRNs by Differential Equations

The time evolution of states with the probabilistic transitions is described by a chemical master equation (CME) (Andreychenko et al., 2011; Weber and Frey, 2017). The CME is a set of coupled first-order ODEs or partial differential equations (PDEs) (Fearnhead et al., 2014; Penas et al., 2017; Teijeiro et al., 2017) representing a continuous time approximation and describing the BRN quantitatively. The ODE model of a BRN can be also derived as a low-order moment approximation of the CME (Bogomolov et al., 2015). For the models with stochastic differential equations (SDEs), it is often difficult to find the transition probabilities (Karimi and Mcauley, 2013; Fearnhead et al., 2014; Sherlock et al., 2014). The PDE approximation can be obtained assuming a Taylor expansion of the CME (Schnoerr et al., 2017). The error bounds for the numerically obtained stationary distributions of the CME are obtained in Kuntz et al. (2017). The CME for a hierarchical BRN consisting of the dependent and independent sub-networks is solved analytically in Reis et al. (2018). A path integral form of the ODEs has been considered in Liu and Gunawan (2014) and Weber and Frey (2017). The BRN models with memory described by the delay differential equations (DDEs) are investigated in Zhan et al. (2014). The mixed-effect models assume multiple instances of the SDE based models to evaluate statistical variations between and within these models (Whitaker et al., 2017).

A comprehensive tutorial on the ODE modeling of biological systems is provided in Gratie et al. (2013). The ODE models can be solved numerically via discretization. For instance, the finite differences method (FDM) can be used to obtain difference equations (Fröhlich et al., 2016). However, the algorithms for numerically solving the deterministic ODE models or simulating the models with SDEs may not be easily parallelizable, and they may have problems with numerical stability. The ODE models are said to be stiff, if they are difficult to solve or simulate, for example, if they comprise multiple processes at largely different time scales (Sun et al., 2012; Cazzaniga et al., 2015; Kulikov and Kulikova, 2017). Alternatively, the BRN structure can be derived from its ODE representation (Fages et al., 2015). A similar strategy is assumed in Plesa et al. (2017) where the BRN is inferred from the deterministic ODE representation of the time series data.

A survey of methods for solving the CME of gene expression circuits is provided in Veerman et al. (2018). These methods involve propagators, time-scale separation, and the generating functions (Schnoerr et al., 2017). For instance, the time-scale separation can be used to robustly decompose the CME into a hierarchy of models (Radulescu et al., 2012). A reduced stochastic description of BRNs exploiting the time-scale separation is studied in Thomas et al. (2012).

If the deterministic ODEs cannot be solved analytically, one can use Langevin and Fokker-Planck equations as the stochastic diffusion approximations of the CME (Hasenauer, 2013; Schnoerr et al., 2017). The Fokker-Planck equation can be solved to obtain a deterministic time evolution of the system state distribution (Kügler, 2012; Liao et al., 2015; Schnoerr et al., 2017). The deterministic and stochastic diffusion approximations of stochastic kinetics are reviewed in Mozgunov et al. (2018). The chemical Langevin equation (CLE) is a SDE consisting of a deterministic part describing the slow macroscopic changes, and a stochastic part representing the fast microscopic changes which are dependent on the size of the deterministic part (Golightly et al., 2012; Cseke et al., 2016; Dey et al., 2018). In the limit, as the deterministic part increases, the random fluctuations can be neglected, and the deterministic kinetics described by the Langevin equation becomes the reaction rate equation (RRE) (Bronstein et al., 2015; Fröhlich et al., 2016;Loos et al., 2016).

### 3.2. Modeling BRNs by Approximations

A popular strategy to obtain computationally efficient models is to assume approximations, such as meta-heuristics and meta-modeling (Sun et al., 2012; Cedersund et al., 2016). The quasi-steady state (QSS) and quasi-equilibrium (QE) approximations of BRNs are investigated in Radulescu et al. (2012). The modifications of QSS models are investigated in Wong et al. (2015). It is also common to approximate the system dynamics assuming continuous ODEs or SDEs (Fearnhead et al., 2014). The SDE model is preferred when the number of molecules is small, since the deterministic ODE model may be inaccurate (Gillespie and Golightly, 2012). It is generally difficult to quantify the approximation errors in the diffusion-based models. The forward-reverse stochastic diffusion with the deterministic approximation of propensities by the observed data was considered in Bayer et al. (2016).

The mass action kinetics can be used to obtain a deterministic approximation of CME. The corresponding deterministic ODEs can accurately describe the system dynamics, provided that the molecule counts of all the species are sufficiently large (Sherlock et al., 2014; Yenkie et al., 2016). Other CME approximations assume the finite state projections, the system size expansion, and the moment closure methods (Chevaliera and Samadb, 2011; Schnoerr et al., 2017). These methods are attractive, since they are easy to implement and efficient computationally. They do not require the complete statistical description, and they achieve good accuracy if the species appear in large copy numbers (Schnoerr et al., 2017). The moment closure methods leading to the coupled ODEs can approach the CME solution with a low computational complexity (Bogomolov et al., 2015; Fröhlich et al., 2016; Schilling et al., 2016). Specifically, the *n*-th moment of the population size depends on its (*n*+1) moment, and to close the model, the (*n*+1)-th moment is approximated by a function of the lower moments (Ruess et al., 2011; Ghusinga et al., 2017). Only the first several moments can be used to approximate the deterministic solution of CME (Schnoerr et al., 2017). The limitations of the moment closure methods are analyzed in Bronstein and Koeppl (2018). A multivariate moment closure method is developed in Lakatos et al. (2015) to describe the non-linear dynamics of stochastic kinetics. The general moment expansion method for stochastic kinetics is derived in Ale et al. (2013). The approximations of the state probabilities by their statistical moments can be used to conduct efficient simulations of stochastic kinetics (Andreychenko et al., 2015).

The leading term of the CME approximation in the system size expansion (SSE) method corresponds to a linear noise approximation (LNA). It is the first order Taylor expansion of the deterministic CME with a stochastic component where the transition probabilities are additive Gaussian noises. Other terms of the Taylor expansion can be included in order to improve the modeling accuracy (Fröhlich et al., 2016). In Sherlock et al. (2014), the LNA is used to approximate the fast chemical reactions as a continuous time Markov process (CTMP) whereas the slow reactions are represented as a Markov jump process with the time-varying hazards. There are other variants of the LNA, such as a restarting LNA model (Fearnhead et al., 2014), the LNA with time integrated observations (Folia and Rattray, 2018), and the LNA with time-scale separation (Thomas et al., 2012). The LNA for the reaction-diffusion master equation (RDME) is computed in Lötstedt (2018). The impact of parameter values on the stochastic fluctuations in a LNA of BRN is investigated in Pahle et al. (2012).

The so-called S-system model is a set of decoupled non-linear ODEs in the form of product of power-law functions (Chou et al., 2006; Meskin et al., 2011; Liu et al., 2012; Iwata et al., 2014). Such models are justified by assuming a multivariate linearization in the logarithmic coordinates. These models provide a good trade-off between the flexibility and accuracy, and offer other properties which are particularly suitable for modeling complex non-linear systems. The S-system models with additional constraints are assumed in Sun et al. (2012). The S-system modeling of biological pathways is investigated in Mansouri et al. (2015). The S-system model with weighted kinetic orders is obtained in Liu and Wang (2008a). The Bayesian inference for S-system models is investigated in Mansouri et al. (2014).

Polynomial models of biological systems are investigated in Kuepfer et al. (2007), Vrettas et al. (2011), Fey and Bullinger (2010), and Dattner (2015). Rational models as fractions of polynomial functions are examined in Fey and Bullinger (2010), Eisenberg and Hayashi (2014), and Villaverde et al. (2016). The methods for validating polynomial and rational models of BRNs are studied in Rumschinski et al. (2010). The eigenvalues are used in Hori et al. (2013) to obtain a low order linear approximation of the time series data. More generally, the models with differential-algebraic equations (DAEs) are considered in Ashyraliyev et al. (2009), Michalik et al. (2009), Rodriguez-Fernandez et al. (2013), and Deng and Tian (2014). These models have different characteristics than the ODE based models, and they are also more difficult to solve. The review of autoregressive models for parameter inferences including the stability and causality issues is presented in Michailidis and dAlchéBuc (2013).

### 3.3. Other Models of BRNs

There are many other types of BRN models considered in the literature. The birth-death process is a special case of the CTMP having only two states (Daigle et al., 2012; Paul, 2014; Zechner, 2014). It is closely related to a telegraph process (Veerman et al., 2018). A computationally efficient tensor representation of BRNs to facilitate the parameter estimation and sensitivity analysis is devised in Liao et al. (2015). Other computational models for a qualitative description of interactions and behavioral logic in BRNs involve the Petri nets (Mazur, 2012; Sun et al., 2012; Schnoerr et al., 2017), the probabilistic Boolean networks (Liu et al., 2012; Mazur, 2012; Mizera et al., 2014), the continuous time recurrent neural networks (Berrones et al., 2016), and the agent based models (ABMs) (Hussain et al., 2015). The hardware description language (HDL) originally devised to describe the logic of electronic circuits is adopted in Rosati et al. (2018) to model spatially-dependent biological systems with the PDEs. The multi-parameter space was mapped onto a 1D manifold in Zimmer et al. (2014).

The hybrid models generally combine different modeling strategies in order to mitigate various drawbacks of specific strategies (Mikeev and Wolf, 2012; Sherlock et al., 2014; Babtie and Stumpf, 2017). For example, a hybrid model can assume deterministic description of large species populations with the stochastic variations of small populations (Mikeev and Wolf, 2012). The hybrid model consisting of the parametric and non-parametric sub-models can offer some advantages over mechanistic models (von Stosch et al., 2014).

The modeling strategies discussed in this section are summarized in Table 1. The models are loosely categorized as physical laws, random processes, mathematical models, interaction models and the CME based models. These models are mostly quantitative except the interaction based models which are qualitative. Note that the model properties, such as sloppiness, and the model structures which may be hierarchical, modular or sequential are not distinguished in Table 1.

**Table 1:** An overview of the main modeling strategies for BRNs.

**Strategy**	**Motivation and key papers**
**Physical laws**	Reaction rates in dynamic equilibrium are functions of reactant concentrations
• Kinetic rate laws	Joshia et al., 2006; Chou and Voit, 2009; Engl et al., 2009; Baker et al., 2011; Villaverde et al., 2012; Voit, 2013
• Mass action kinetics	Angius and Horváth, 2011; Lindera and Rempala, 2015; Wong et al., 2015; Smith and Grima, 2018
• Mechanistic models	Chou and Voit, 2009; Pullen and Morris, 2014; von Stosch et al., 2014; White et al., 2016
**Random processes**	Probabilistic behavioral description of chemical reactions
• Markov process	Andrieu et al., 2010; Goutsias and Jenkinson, 2013; Septier and Peters, 2016; Weber and Frey, 2017
• Poisson process	Daigle et al., 2012; Weber and Frey, 2017; Bronstein and Koeppl, 2018; Reis et al., 2018
• Birth-death process	Wang et al., 2010; Daigle et al., 2012; Mikelson and Khammash, 2016; Weber and Frey, 2017
• Telegraph process	Weber and Frey, 2017; Veerman et al., 2018
**Mathematical models**	Adopted models for dynamic systems
• Quasi-state models	Radulescu et al., 2012; Srivastava, 2012; Thomas et al., 2012; Wong et al., 2015; Liao, 2017; Schnoerr et al., 2017
• State space representation	Andrieu et al., 2010; Andreychenko et al., 2011; Brim et al., 2013; Weber and Frey, 2017
• ODEs, PDEs, SDEs, DDEs	J. O. Ramsay and Cao, 2007; Jia et al., 2011; Liu and Gunawan, 2014; Fages et al., 2015; Teijeiro et al., 2017; Weber and Frey, 2017
• Path integral form of ODEs	Weber and Frey, 2017
• Rational model	Sun et al., 2012; Vanlier et al., 2013; Hussain et al., 2015; Villaverde et al., 2016
• Differential algebraic eqns.	J. O. Ramsay and Cao, 2007; Ashyraliyev et al., 2009; Michalik et al., 2009; Deng and Tian, 2014
• Tensor representation	Liao et al., 2015; Wong et al., 2015; Smith and Grima, 2018
• S-system model	Kutalik et al., 2007; Chou and Voit, 2009; Meskin et al., 2011; Liu et al., 2012; Voit, 2013
• Polynomial model	Vrettas et al., 2011; Češka et al., 2017; Kuntz et al., 2017; Weber and Frey, 2017
• Manifold map	Radulescu et al., 2012; Mannakee et al., 2016; Septier and Peters, 2016; White et al., 2016
**Interaction models**	Qualitative modeling of chemical interactions
• Petri nets	Chou and Voit, 2009; Liu et al., 2012; Voit, 2013
• Boolean networks	Chou and Voit, 2009; Emmert-Streib et al., 2012
• Neural networks	Goutsias and Jenkinson, 2013; von Stosch et al., 2014; Ali et al., 2015; Camacho et al., 2018
• Agent based models	Carmi et al., 2013; Goutsias and Jenkinson, 2013; Hussain et al., 2015; Jagiella et al., 2017
**CME based models**	Stochastic and deterministic approximations of CME
• Langevin equation	Thomas et al., 2012; Goutsias and Jenkinson, 2013; Septier and Peters, 2016; Schnoerr et al., 2017; Weber and Frey, 2017; Smith and Grima, 2018
• Fokker-Planck equation	Liao et al., 2015; Schnoerr et al., 2017; Weber and Frey, 2017
• Reaction rate equation	Koeppl et al., 2012; Liu and Gunawan, 2014; Lindera and Rempala, 2015; Loos et al., 2016
• Moment closure	Ruess et al., 2011; Andreychenko et al., 2015; Lakatos et al., 2015; Schilling et al., 2016; Schnoerr et al., 2017; Bronstein and Koeppl, 2018
• Linear noise approximation	Golightly et al., 2012, 2015; Thomas et al., 2012; Fearnhead et al., 2014; Schnoerr et al., 2017; Whitaker et al., 2017
• System size expansion	Fröhlich et al., 2016; Schnoerr et al., 2017

**Figure 2:**
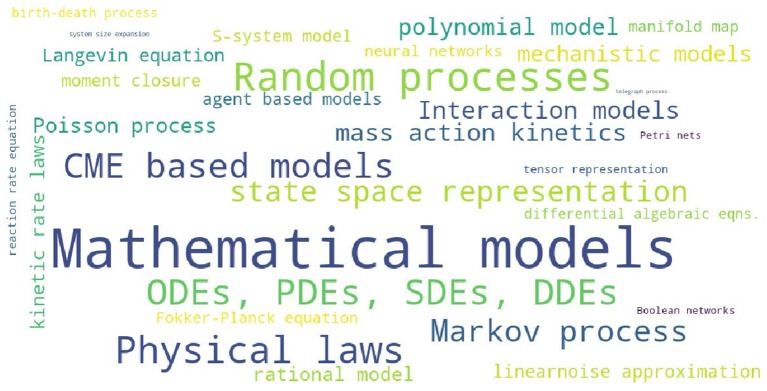
A word cloud visualizing the levels of interest in different models of BRNs.

In order to assess the level of interest in different BRN models in literature, Table S1 presents the number of occurrences for the 25 selected modeling strategies in all references cited in this review. The summary of Table S1 is reproduced in Table 2 with the inserted bar graph, and further visualized as a word cloud in Figure 2. We observe that differential equations are the most commonly assumed models of BRNs in the literature. About half of the papers cited consider the Markov chain models or their variants, since these models naturally and accurately represent the time sequences of randomly occurring reactions in BRNs. The state space representations are assumed in over one third of the cited papers. Other more common models of BRNs include the mass action kinetics, mechanistic models, and the models involving polynomial functions.

**Table 2:**
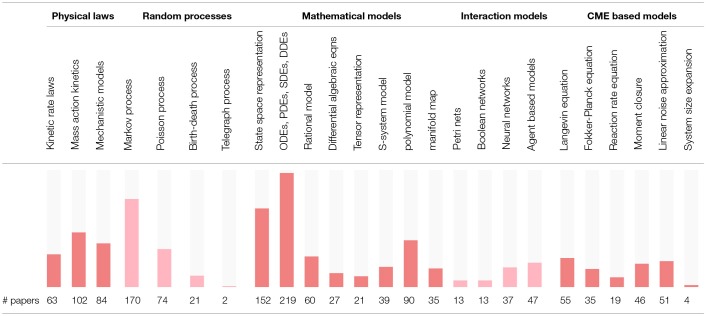
The coverage of modeling strategies for BRNs.

Another viewpoint on BRN models in literature is to consider the publication years of papers. Table 3 shows the number of papers for a given modeling strategy in a given year starting from the year 2005. The dot values in tables represent zero counts to improve the readability. We can observe that the interest in some modeling strategies remain stable over the whole decade, for example, for the models involving state space representations and the models involving differential equations. The number of cited papers is the largest in years 2013 and 2014. The paper counts in Table 3 indicate that the interest in computational modeling of BRNs has been increasing steadily over the past decade.

**Table 3:**
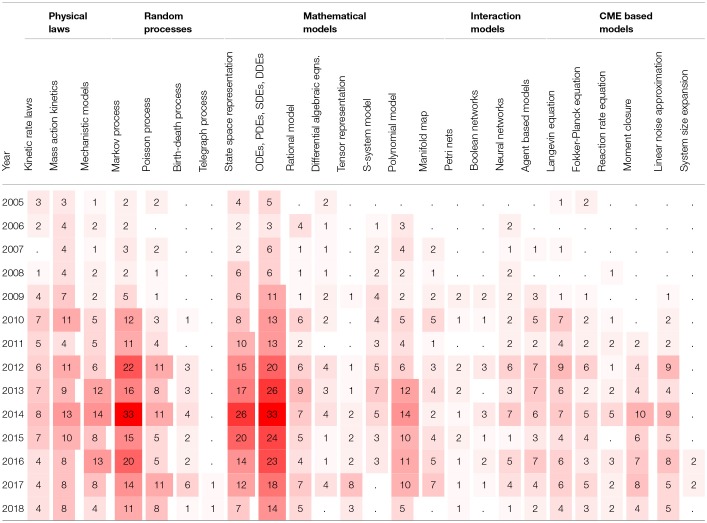
The number of papers concerning models of BRNs in given years.

## 4. Review of Parameter Estimation Strategies for BRNs

The parameter estimation or inference appears in many other computational problems including model identification (Banga and Canto, 2008), model calibration (Zechner et al., 2011), model discrimination (Kuepfer et al., 2007), model identifiability (Geffen et al., 2008), model checking (Hussain et al., 2015), sensitivity analysis (Erguler and Stumpf, 2011), optimum experiment design (Ruess and Lygeros, 2015), bifurcation analysis (Engl et al., 2009), reachability analysis (Tenazinha and Vinga, 2011), causality analysis (Carmi et al., 2013), stability analysis (Dochain, 2003), network inference (Smet and Marchal, 2010), and network control (Venayak et al., 2018). A chemical reaction optimization (CRO) can be used to maximize the production of a bio-reactor (Abdullah et al., 2013b). The surveys of parameter estimation methods for chemical reaction systems can be found, for example, in Chou and Voit (2009), Gupta (2013), Baker et al. (2015), and McGoff et al. (2015). Other review papers on parameter estimation in BRNs and dynamic systems are listed in Table 4.

**Table 4:** The review papers on the parameter estimation in BRNs and other dynamic systems.

**Reference**	**Focus**
Banga and Canto, 2008	Model calibration using global optimization methods supported by maximum information experiment design
Chou and Voit, 2009	Very comprehensive survey of available optimization methods for parameter estimation and model-free and model-based structure identification from data
Ashyraliyev et al., 2009	*A priori* and a posteriori model identifiability and survey of parameter space search strategies
Smet and Marchal, 2010	Methods for under-determined inferences of BRNs from data
Tenazinha and Vinga, 2011	Integrated models of BRNs reflecting availability of omics data assuming chemical organization theory, flux-balance analysis, logical discrete modeling, Petri nets, kinetic models, stochastic models, and hybrid models
Daigle et al., 2012	Survey of maximum-likelihood based methods
Emmert-Streib et al., 2012	Systematic and conceptual overview of methods for inferring gene regulatory networks from gene expression data; survey of strategies to compare performance of inference methods
Sun et al., 2012	Survey of metaheuristic methods applied to reliability and identifiability of biochemical model parameters including optimum experiment design
Goutsias and Jenkinson, 2013	Comprehensive review of analytical methods for evaluating dynamics of Markov reaction networks
Kuwahara et al., 2013	Scalable framework for parameter estimation in genetic circuits assuming mean time evolution of gene products
Voit, 2013	Review of biological system models and methods for their analysis as well as design
Baker et al., 2015	General framework to deal with non-identifiable parameters in BRNs using constrained parameter estimation
McGoff et al., 2015	Mathematical survey of statistical methods for parameter inference in general non-linear dynamical systems
Drovandi et al., 2016	Survey of approximate Bayesian computation methods
Kurt et al., 2016	Review of 27 estimators of association scores of data from gene networks
Weiss et al., 2016	Survey of transfer learning methods
Schnoerr et al., 2017	A comprehensive survey of deterministic and stochastic models of BRNs followed by introduction to Bayesian parameter inference from data
Camacho et al., 2018	Application of machine learning techniques to computational problems in biological networks
Smith and Grima, 2018	Review of spatial stochastic kinetics including reaction-diffusion master equation and models involving Brownian dynamics
Koblents et al., 2019	Bayesian inference methods with stochastic kinetic models

A survey of tasks concerning modeling and system identification is provided in Chou and Voit (2009). The model identifiability determines which parameters can be estimated from observations (Villaverde et al., 2016). It is inspired by the concept of system observability and known as a structural identifiability. It is useful to consider the structural identifiability prior to estimating the parameters. There is also a practical identifiability which accounts for the quality and quantity of observations, i.e., whether it is possible to obtain good parameter estimates from noisy and limited data. The theory and tools for the model identifiability and other closely related concepts, such as the sensitivity to parameter perturbations, the observability, the distinguishability and the optimum experiment design are reviewed in Villaverde and Barreiro (2016). The models which are not identifiable can be modified or simplified to make them identifiable (Baker et al., 2015; Villaverde and Barreiro, 2016; Villaverde et al., 2016). The model identifiability is formulated as the model observability in Geffen et al. (2008) by replacing traditional analytical approaches which often require model simplifications with other deterministic empirical methods.

The changes in the structural and practical identifiability of models when new knowledge and data become available is studied in Babtie and Stumpf (2017). The global observability and detectability of reaction systems was studied in Moreno and Denis (2005). The parameter identifiability of the power law models is investigated in Srinath and Gunawan (2010) and of the linear dynamic models in Li and Vu (2013). The parameter dependencies are considered in Li and Vu (2015) to determine the structural and practical identifiability. The intrinsic noise in the species counts can be exploited to overcome the structural non-identifiability within a deterministic framework as shown in Zimmer et al. (2014).

In general, many different parameter estimation methods have been devised in literature for BRNs and dynamic systems. However, many of these methods are often modifications of a few fundamental estimation strategies which are adopted for the specific models and the availability and quality of measurements. All parameter estimation problems lead to the minimization or maximization of some fitness function. Deriving the optimum value analytically is rarely possible whereas a numerical search for the optimum in high-dimensional parameter spaces can be ill-conditioned when the fitness function is multi-modal. The numerical strategies normally experience a trade-off between the efficiency and robustness. If there is a large flat surface about the minimum, the obtained solution cannot be trusted (Rodriguez-Fernandez et al., 2006a; Srinivas and Rangaiah, 2007). Moreover, the optimum values can change over an order of magnitude under different implicit or explicit constraints which is often the case for biological systems. The numerical algorithms for non-convex optimization problems need to be stable as well as provide the convergence guarantees. Other important aspects to consider include scalability, computational efficiency, numerical stability and robustness. All methods need to be also statistically validated.

The measurements can be produced from different heterogeneous sources (omics data), and from heterogeneous populations (Zechner et al., 2011). In literature, the deterministic methods appear to be assumed much more often than the stochastic methods (Daigle et al., 2012). The parameter estimation in deterministic models is often carried out by fitting the model to the data. The parameter uncertainty analysis can be used to assess how well the model explains the experimental data (Vanlier et al., 2013). The stochastic models require more sophisticated strategies to perform parameter estimation (Zimmer and Sahle, 2012), such as the multiple-shooting methods (Zimmer, 2016). Moreover, since the mean approximation of SDEs may differ from the solution obtained for deterministic ODEs, the parameter estimation assuming stochastic rather than deterministic models is preferable when some of the species counts are relatively small (Andreychenko et al., 2012).

The parameter estimations in the transient and at steady state are quite different (Ko et al., 2009). At steady state, small perturbations are sufficient to observe the system responses whereas at the transient state, the experiment design for model identification is more complicated. A fast transient response after the external perturbation limits the information content in measurements (Zechner et al., 2012). The sensitivity analysis can be used to improve the computational efficiency of parameter estimation (Fröhlich et al., 2017). The parameter value boundaries can be estimated by sampling (Fey and Bullinger, 2010). The confidence and credible intervals can be obtained also for the stiff and sloppy models assuming the inferability, sensitivity and sloppiness (Erguler and Stumpf, 2011). Furthermore, the observer design may be different for systems with and without inputs (Singh and Hahn, 2005).

The scalability of parameter estimation can be resolved by decoupling the rate equations and by assuming the mean-time evolution of the species counts (Kuwahara et al., 2013). However, exploring large parameter spaces can be complicated, if the estimation problems are ill-conditioned and multi-modal (Liu and Wang, 2009). The state-dependent Markov jump processes are difficult to estimate at large scale, especially when these processes are faster than the rate of observations (Fearnhead et al., 2014).

The model parameters can be mutually dependent (Fey et al., 2008). The parameter dependencies can be measured by correlations and other higher order moments. The parameter estimation can be facilitated by grouping the parameters, and then identifying which are uncorrelated (Gábor et al., 2017). The parameter estimation in groups can provide robustness against the noisy and incomplete data (Jia et al., 2011). Only the parameters which are consistent with the measured data can be selected and jointly estimated (Hasenauer et al., 2010). The parameter clustering can also improve the model tractability and identifiability, since the changes in some parameters could be compensated by changes in other parameters (Nienaltowski et al., 2015). The groupings of parameters to elucidate the dynamics of genetic circuits are assumed in Atitey et al. (2019). The parameters can be assumed hierarchically to gradually estimate their values starting from a suitably defined minimum set (Shacham and Brauner, 2014). A hybrid hierarchical parameter estimation method which is prone to parallel implementation is devised in He et al. (2004).

An incremental parameter estimation usually requires data smoothing which can create the estimation biases (Liu and Gunawan, 2014). Such biases can be mitigated by estimating the independent parameters before the dependent ones. The parameter inference can be paired with the hypothesis testing and model selection (Rodriguez-Fernandez et al., 2013). The joint model and parameter identification with incremental one-at-a-time parameter estimation and model building is performed in Gennemark and Wedelin (2007). The unobserved states, latent variables and other parameters in BRNs can be estimated jointly by sequentially processing the measurements (Zimmer and Sahle, 2012; Arnold et al., 2014), by using the sliding window observers (Liu et al., 2006), and by other numerical methods (Karnaukhov et al., 2007). The estimation of kinetic rates in BRNs is transformed into a problem of the state estimation in Fey and Bullinger (2010). The parameter estimation and the state reconstruction are linked via the extended models in Busetto and Buhmann (2009). The unobservable sub-spaces can be excluded, and only the model parts which are identified reliably can be considered (Singh and Hahn, 2005). Another strategy is to reconstruct the states prior to estimating the parameters (Fey et al., 2008). The unknown parameters which are not of interest can be margninalized (Bronstein et al., 2015).

The model overfitting leads to a poor generalization capability. In order to avoid the overfitting and to constrain the model complexity, a penalty can be assumed to minimize the number of estimated model parameters. The overfitting can be resolved by the model reduction techniques (Srivastava, 2012; Sadamoto et al., 2017). For instance, only essential chemical reactions can be considered in BRN model (Zamora-Sillero et al., 2011). A simplified modeling with the reduced number of parameters and the parameter subset selection is used in Eghtesadi and Mcauley (2014) to avoid overfitting the noisy data. On the other hand, the under-determined models may yield several or infinitely many solutions of fitting the data. In such cases, the models are not identifiable, and the data fitting can be performed subject to additional constraints. There are also cases where the measured data can be fit well by several models. However, the model with the best fit to the data may not necessarily provide a satisfactory biological explanation (Slezak et al., 2010).

The information theoretic metrics can be used to infer the structure of BRNs (Villaverde et al., 2014), and to perform the identifiability analysis of parameters (Nienaltowski et al., 2015). Akaike information is used to assess the quality of statistical models given observations, so the best model can be selected (Guillén-Gosálbez et al., 2013; Pullen and Morris, 2014). The simultaneous estimation of parameters and the structure of BRN formulated as a mixed binary dynamic optimization problem with Akaike information is assumed in Guillén-Gosálbez et al. (2013) to trade-off the estimation accuracy and the evaluation complexity. Fisher information is the mean amount of information gained from the observed data. It is often used when estimating the non-random parameters, for instance, using the maximum likelihood (ML) (Rodriguez-Fernandez et al., 2006b; Kyriakopoulos and Wolf, 2015). Fisher information can be exploited to perform the sensitivity, robustness and identifiability of parameters. It is especially useful when the measurements and parameters are correlated (Komorowski et al., 2011). Fisher information is also used to improve the parameter estimation (Transtrum and Qiu, 2012), to design the optimum experiments (Kyriakopoulos and Wolf, 2015; Zimmer, 2016), and to select the subsets of identifiable parameters (Eisenberg and Hayashi, 2014). Mutual information can be used as a similarity measure. It statistically outperforms correlations in the canonical correlation analysis (CCA) (Nienaltowski et al., 2015). Other uses of mutual information are outlined in Mazur (2012), and for the parameter estimation in Emmert-Streib et al. (2012).

The cross-entropy methods can be combined with stochastic simulations (Revell and Zuliani, 2018), and used to improve the computational efficiency of the parameter estimation (Daigle et al., 2012). The maximum entropy sampling (MES) methods for the experiment design and for the parameter estimation are discussed in Mazur and Kaderali (2013). The maximum entropy principle to reconstruct the probability distributions is described in Schnoerr et al. (2017). The relative entropy rate is assumed in Pantazis et al. (2013) to perform the sensitivity analysis of BRNs. The Kantorovich distance between two probability measures is used in Koeppl et al. (2010) to estimate the BRN model parameters.

The sum of squared errors (SSE) is often assumed to define the regression estimators (Chou et al., 2006), to evaluate the goodness of fit, and to assess the quality of estimators (Nim et al., 2013; Iwata et al., 2014; Kimura et al., 2015). The SSE acronym should not be confused with the system size expansion (SSE) which is a modeling strategy discussed previously (Fröhlich et al., 2016; Schnoerr et al., 2017).

Furthermore, the graduate research theses usually contain more or less comprehensive and up to date surveys of the relevant literature. The theses which are concerned with the parameter estimation in BRNs are summarized in Table 5. We can observe that the largest number of the research theses involving the parameter estimation problems in BRNs were produced in 2014.

**Table 5:** The selected research theses concerning the parameter estimation and related problems in BRNs.

**Thesis**	**Main research focus**
Dargatz, 2010	Bayesian inference for biochemical models involving diffusion
Mu, 2010	Rate and state estimation in S-system and linear fractional model (LFM)
Palmisano, 2010	Software tools for modeling and parameter estimation in BRNs
Mazur, 2012	Inference via stochastic sampling and Bayesian learning framework
Srivastava, 2012	Stochastic simulations of BRNs combined with likelihood based parameter estimation, confidence intervals, sensitivity analysis
Gupta, 2013	Parameter estimation in deterministic and stochastic BRNs, inference with model reduction, mostly MCMC methods
Hasenauer, 2013	Bayesian estimation and uncertainty analysis of population heterogeneity and proliferation dynamics
Linder, 2013	Penalized LS algorithm and diffusion and linear noise approximations and algebraic statistical models
Flassig, 2014	Model identification for large scale gene regulatory networks
Liu, 2014	Approximate Bayesian inference methods and sensitivity analysis
Moritz, 2014	Structural identification and parameter estimation for modular and layered type of modes
Paul, 2014	Analysis of MCMC based methods
Ruess, 2014	Optimum estimation and experiment design assuming ML and Bayesian inference and Fisher information
Schenkendorf, 2014	Quantification of parameter uncertainty, optimal experiment design for parameter estimation and model selection
Smadbeck, 2014	Moment closure methods, model reduction, stability and spectral analysis of BRNs
Zechner, 2014	Inference from heterogeneous snapshot and time-lapse data
Schnoerr, 2016	Langevin equation, moment closure approximations, representations of stochastic RDME
Galagali, 2016	Bayesian and non-Bayesian inference in BRNs, adaptive MCMC methods, network-aware inference, inference for approximated BRNs
Hussain, 2016	Sequential probability ratio test, Bayesian model checking, automated and formal verification, parameter discovery
Lakatos, 2017	Multivariate moment closure and reachability analysis
Liao, 2017	Tensor representation and analysis of BRNs

In the rest of this section, we will survey specific methods for the parameter estimation in BRNs. These methods are organized in the following four subsections: Bayesian methods, Monte Carlo methods, other statistical methods including Kalman filtering, and the model fitting methods.

### 4.1. Bayesian Methods

The fundamental premise of the Bayesian estimation methods is that the prior probabilities or distributions of parameters are known. The objective is then to evaluate the posterior distributions for the parameters of interest. It is often sufficient to find the maximum value of the posterior distribution as the maximum a posterior (MAP) estimate. The value of this maximum can be also used to select among several competing models (Andreychenko et al., 2012) and to design the optimum experiments (Mazur, 2012). The model checking via the time-bounded path properties is represented as the Bayesian inference problem in Milios et al. (2018). The conjugate priors are often assumed in biological models to perform the Bayesian inferences (Boys et al., 2008; Mazur, 2012; Murakami, 2014; Galagali, 2016). The Bayesian inference for the low copy counts can be improved by separating the intrinsic and extrinsic noises (Koeppl et al., 2012). The Bayesian analysis is facilitated by separating the slow and fast reactions in Sherlock et al. (2014). The Bayesian inference strategies for biological models involving diffusion processes are investigated in Dargatz (2010).

In many cases, determining the exact posterior distribution in the Bayesian analysis is analytically intractable. The approximate Bayesian computation (ABC) is a computational strategy for estimating the posterior distribution or the likelihood function (Tanevski et al., 2010). The survey of ABC approaches is provided in Drovandi et al. (2016). The basic idea is to find the parameter values which can generate the same statistics as the observed data. The ABC can be performed sequentially, and used for the sensitivity analysis (Liu, 2014). The parameter estimation and the model selection using the ABC framework is studied in Liepe et al. (2014) and Murakami (2014). The non-identifiability of parameters due to the flat-shaped posterior can be resolved by the ABC approach as shown in Murakami (2014). The efficient generation of summary statistics for the ABC is presented in Fearnhead and Prangle (2012). The piece-wise ABC to estimate the posterior density for Markov models is proposed in White et al. (2015). The parallel implementations of the ABC and SMC methods are introduced in Jagiella et al. (2017).

The expectation-maximization (EM) is a popular implementation of the MAP estimators where there are some other unobserved or unknown parameters (Daigle et al., 2012; Karimi and Mcauley, 2014a; Bayer et al., 2016). The EM can be combined with the Monte Carlo (MC) sampling, and such methods are known as the MC expectation-maximization (MCEM) (Angius and Horváth, 2011). The computationally efficient method for obtaining the ML estimates by the MCEM with a modified cross-entropy method (MCEM2) is developed in Daigle et al. (2012). The approximate EM algorithm is devised in Karimi and Mcauley (2013) which is robust against the unknown initial estimates, and which is useful for the online state estimation during the process monitoring.

Another parameter estimation strategy having the same structure as the EM is known as the variational Bayesian inference (Vrettas et al., 2011; Weber and Frey, 2017). It is more general than the EM method, and it exploits the analytical approximations of the posterior density to obtain the parameter estimates and their likelihoods. The analytical approximations are usually computationally faster than the sampling based methods, but the approximation methods are still less well-understood (Blei et al., 2017). For instance, the posterior density is approximated by radial basis functions (RBFs) in Fröhlich et al. (2014) to reduce the number of model evaluations. The variational inference with stochastic approximations for Gaussian mixture models and massive data is considered in Blei et al. (2017). The variational approximate inference with the continuous time constraints is investigated in Cseke et al. (2016).

The ML estimation is a popular parameter estimation strategy, provided that the likelihoods of the observed data can be computed efficiently for the given model. The survey of ML based methods for the parameter estimation in BRNs is provided in Daigle et al. (2012). The likelihood function can be approximated analytically using the Laplace and the B-spline approximations (Karimi and Mcauley, 2014b), or numerically by assuming the derivatives (Mikeev and Wolf, 2012). The likelihood function is obtained by simulations in Tian et al. (2007). The moment closure is used for the fast approximations of the parameter likelihoods in Milner et al. (2013). Stochastic simulations can be avoided by approximating the transition distributions by the Gaussian distribution in the parameter likelihood calculations (Zimmer and Sahle, 2015). In Chen et al. (2017), the transition probabilities are used in the ML calculations to devise the new estimation algorithm which can improve the variational Bayesian inference. The ML estimation combined with regularization to penalize the complexity is investigated in Jang et al. (2016). The ML estimation for BRN models with the concentration increments and decrements is studied in Lecca et al. (2009).

### 4.2. Monte Carlo Methods

The motivation behind the MC methods is to represent the probabilities and density functions as the relative frequencies of samples or particles in order to overcome mathematical intractability of the Bayesian inference. However, even the sampling methods can be computationally overwhelming due to frequent model evaluations. The Markov chain Monte Carlo (MCMC) methods are the most often used sampling strategies to generate conditional trajectories of the system states. The MCMC sampling having good mixing properties requires a carefully chosen proposal distribution and also a good selection of the initial samples in order to avoid the sample degeneracy and instability problems. The most well-known sampling MCMC procedures are the Metropolis and the Metropolis-Hastings algorithms (Golightly and Wilkinson, 2011; Zamora-Sillero et al., 2011; Mazur, 2012; Galagali, 2016). An overview of the particle filtering and the MCMC methods for the spatial objects tracking is presented in Mihaylova et al. (2014). The MCMC methods for causality reasoning are introduced in Carmi et al. (2013). The design of proposal distributions for the MCMC and the SMC methods assuming a large number of correlated variables is studied in Andrieu et al. (2010).

Since the convergence rate of the MCMC sampling can be rather slow for heavy tail distributions, the factorization and approximations of the posterior can be used to improve the performance (Fröhlich et al., 2014). The MCMC methods can be made adaptive to improve their convergence properties as shown in Mazur (2012); Müller et al. (2012); Hasenauer (2013); Galagali (2016). The interpolation of the observed data via the MCMC sampling is assumed in Golightly and Wilkinson (2005) to jointly estimate the unobserved states and reaction rates. The MCMC sampling can be combined with the importance sampling to reduce the computational complexity and simulation times (Golightly et al., 2015). The conditional density importance sampling (CDIS) is introduced in Gupta and Rawlings (2014) as an alternative to the MCMC parameter estimation.

A strategy for dealing with high-dimensional sampling problems is to combine the particle filters with the MCMC methods to obtain the sequential MCMC (SMCMC) algorithms (Septier and Peters, 2016). The MCMC methods for high-dimensional systems are compared in Septier and Peters (2016). The population MC (PMC) sampling framework to perform the Bayesian inference in high-dimensional models is developed in Koblents and Míguez (2011).

The Bayesian inference via the MC sampling utilizing the stochastic gradient descent is studied in Wang et al. (2010). The parameter likelihoods are calculated by combining the MC global sampling with the locally optimum gradient methods in Kimura et al. (2015). The nested Bayesian sampling is used in Pullen and Morris (2014) to compute the marginal likelihoods, and to compare or rank several competing models. The MCMC sampling for the mixed-effects SDE models is considered in Whitaker et al. (2017). In order to overcome the ill-conditioned least squares (LS) data fitting and the associated numerical instability problems, the bootstrapped MC procedure based on the diffusion and the LNA was proposed in Lindera and Rempala (2015).

The sequential MC (SMC) methods represent the posterior distribution by a set of samples referred to as particles (Gordon et al., 1993; Doucet et al., 2001; Tanevski et al., 2010; Yang et al., 2014), so these methods are also known as particle filters (Gordon et al., 1993; Doucet et al., 2001; Lillacci and Khammash, 2012; Golightly et al., 2015). The particle filters assume specific types of random processes to identify the posterior while bounding the computational complexity for the models with large number of parameters (Mikelson and Khammash, 2016). The particle filters are shown to be more robust than the LS data fitting, if the data statistics are exploited (Lillacci and Khammash, 2012). The SMC methods for the joint estimation of states and parameters are developed in Nemeth et al. (2014). The degeneracy phenomenon commonly occurring in particle filters can be mitigated by more efficient sampling strategies (Golightly and Kypraios, 2017). A parallelization of the SMC computations is devised in Mihaylova et al. (2012). More efficient generation and processing of particles to improve the computational efficiency of particle filters is investigated in Golightly et al. (2019). The computationally efficient particle MCMC (pMCMC) method is devised in Koblents and Míguez (2014) and Koblents et al. (2019). The pMCMC method can be combined with the diffusion approximation (Golightly and Wilkinson, 2011), and further refined to improve its scalability (Golightly and Kypraios, 2017). The proposal distribution for the Bayesian analysis is obtained using the pMCMC sampling in Sherlock et al. (2014). The proposal samples for calculating the marginal likelihoods are obtained for the CLE and the LNA approximations in Golightly et al. (2015).

### 4.3. Other Statistical Methods

The key assumption for using the standard Kalman filter is the linearity of measurements. The Kalman filter is used with the CME approximation and the noise covariance estimation in Dey et al. (2018) while allowing for the dependency of the noise statistics on the states and parameter values. The Kalman filter is used to obtain the initial guess of the parameter values for the subsequent parameter estimation by data fitting in Lillacci and Khammash (2010). The Kalman filter can be merged with the particle filters to perform the inferences in stochastic (Vrettas et al., 2011) as well as deterministic systems (Arnold et al., 2014). The Kalman filter for the time integrated observations is assumed in Folia and Rattray (2018).

Since the BRNs are generally highly non-linear, the extended and unscented Kalman filters (EKFs and UKFs) must be assumed (Baker et al., 2011). The EKF was modified for stiff ODEs in Kulikov and Kulikova (2015a) and Kulikov and Kulikova (2017). The joint estimation of parameters and states by the EKF is investigated in Sun et al. (2008) and Ji and Brown (2009). The EKF is combined with the moment closure methods in Ruess et al. (2011), and it is modified for the parameter estimation in the S-system models in Meskin et al. (2011). A hybrid method combining the EKF and the particle swarm optimization (PSO) for the joint estimation of parameters and states is developed in Zeng et al. (2012). A modified EKF to penalize the modeling uncertainty due to linearization errors is proposed in Xiong and Zhou (2013) which improves the estimation accuracy. The square-root UKF achieves good numerical stability, and it can also assume the state constraints (Baker et al., 2013, 2015). For infrequent sampling and sparse observations, the UKF and the cubature Kalman filter outperform the EKF (Kulikov and Kulikova, 2015b, 2017).

The classical bootstrapping with data replication and resampling to enable the repeated estimations is described in Vanlier et al. (2013). The bootstrapping can be also used to obtain the confidence intervals of the parameter estimates (Joshia et al., 2006; Srivastavaa and Rawlingsb, 2014), and to improve the computational efficiency in recomputed model trajectories (Lindera and Rempala, 2015). The bootstrap filter can outperform the EKF (Gordon et al., 1993).

There are also many other less commonly used inference strategies which have not been mentioned so far. For instance, the Gaussian smoothing to compensate for the missing and noisy data is used in Sun et al. (2012). The parameter estimation assuming a non-linear ODE model combined with the data smoothing was investigated in J. O. Ramsay and Cao (2007). The inference of the state distribution via the optimized histograms and statistical fitting is performed in Atitey et al. (2018b). A formal verification and the sequential probability ratio test for the parameter estimation are considered in Hussain (2016). The moment closure modeling is combined with stochastic simulations for the parameter estimation in Bogomolov et al. (2015). A generalized method of moments incorporating the empirical sample moments is performed in Kügler (2012); Lück and Wolf (2016) whereas the moment based methods for the parameter estimation and the optimum experiment design are considered in Ruess and Lygeros (2015). The expectation propagation (EP) for the approximate Bayesian inference is studied in Cseke et al. (2016). The Lyapunov exponent can be used to infer the level of predictability of the dynamic systems including BRNs (Barnes et al., 2011; McGoff et al., 2015).

### 4.4. Model Fitting Methods

The parameter estimation by fitting the measured data appears to be by far the most commonly used method in literature. The main reason is that, unlike other estimation strategies, the data fitting problem is relatively easy to formulate with minimum knowledge and assumptions. It is possible to consider multiple fitness functions. Various continuous and discrete fitness functions are explored in Deng and Tian (2014). The fitness function can be derived from the likelihood function (Rodriguez-Fernandez et al., 2006a), or the approximated likelihood function (Srivastavaa and Rawlingsb, 2014).

Even though the derivative free methods are easier to implement, the gradient based methods have faster albeit only local convergence. For instance, the gradient based optimization with sensitivity analysis assuming finite differences is investigated in Loos et al. (2016). The derivative free methods are necessary for the combinatorial and the integer constrained problems (Cedersund et al., 2016; Gábor et al., 2017).

The challenge is to develop numerically efficient methods to solve high-dimensional problems with possibly many constraints. The observations are interpolated with the spline functions in Nim et al. (2013), so that the derivatives can be used to estimate the production and consumption of molecules in BRNs. It decomposes a high-dimensional problem into the product of low-dimensional factors. The fitness function is interpolated with the spline functions in Zhan and Yeung (2011).

The data fitting is generally more computationally demanding for stochastic than for deterministic models, but the former is more likely to find a global solution (Rodriguez-Fernandez et al., 2006b). Since many practical optimization problems are non-convex, the global optimization methods are generally preferred. They can be implemented as multi-start or multi-shooting local methods, or by selecting a subset of parameters to be estimated. The sensitivity to initial values can be reduced by tracking multiple solutions. Many of these methods can be readily parallelized to overcome the computational burden (Mancini et al., 2015; Teijeiro et al., 2017). The parallel implementations of data fitting algorithms including Spark, MapReduce, and MPI messaging are considered in Teijeiro et al. (2017). Recently, the implementations exploiting the affordable graphical processing units (GPUs) have become popular (Nobile et al., 2012). The computational complexity of global methods can be mitigated by the incremental identification strategies (Michalik et al., 2009). The global methods also require to properly set the search parameters which can be done via multiple initial exploratory runs (Penas et al., 2017). Another global search strategy assumes a model transformation followed by the non-uniform sampling (Kleinstein et al., 2006). There are also hybrid strategies switching between the global and local searches (Rodriguez-Fernandez et al., 2006a,b; Ashyraliyev et al., 2009).

The majority of data fitting methods are rooted in the simple LS regression, or assume the non-linear least squares (NLSQ) (Baker et al., 2011). The alternating regression (AR) reformulates the non-linear fitting as an iterative linear regression problem (Chou et al., 2006). The non-linear regression is converted into a non-linear programming problem which is solved by the random drift PSO in Sun et al. (2014). The asymptotic properties of the LS estimation were evaluated in Rempala (2012). The iterative linear LS for systems described by a ratio of linear functions is considered in Tian et al. (2010).

The regularization is a strategy to deal with the ill-conditioned optimization problems due to insufficient or noisy data (Gábor and Banga, 2014; Gábor et al., 2017). The regularization introduces additional constraints to penalize the complexity, or it uses prior knowledge to constrain the parameter values to trade-off the estimator bias with its variance in order to avoid the model overfitting (Liu et al., 2012; Kravaris et al., 2013; Jang et al., 2016). Alternatively, the perturbation method has been developed for fitting the data in Shiang (2009).

The evolutionary algorithms (EAs) are the most frequently used methods for solving the high-dimensional constrained optimization problems. They do not require any particular assumptions, and they are not limited by the dimensionality of the problem. The EAs adopt various heuristic strategies to find the optimum assuming the population of candidate solutions which are iteratively improved by reproduction, mutation, crossover or recombination, selection and other operations until the fitness or loss function reaches the desired value. The specific EAs commonly used in literature for the identification of BRNs and other dynamic systems are summarized in Table 6. Several EAs and the PSO methods are compared in Nobile et al. (2018b). Different EAs are compared with other deterministic search methods in Mendes and Kell (1998).

**Table 6:** Common evolutionary algorithms for the parameter estimation in BRNs and dynamic systems.

**Algorithm**	**Motivation and selected papers**
Genetic algorithms (GAs)	Largest class of EAs, inspired by evolution and natural selection, often near optimum solution
	Matsubara et al., 2006; Tian et al., 2007; Besozzi et al., 2009; Chou and Voit, 2009; Liu et al., 2012; Sun et al., 2012
Genetic programming (GP)	Evolution of computer programs toward improving their fitness to solve a given task
	Chou and Voit, 2009; Sun et al., 2012; Nobile et al., 2013
Evolutionary programming (EP)	Parameters of computer program evolve toward improving its fitness to solve a given task
	Baker et al., 2010; Sun et al., 2012; Revell and Zuliani, 2018
Simulated annealing (SA)	Probabilistic search combining sampling with random but controlled acceptance of candidate solutions
	Ashyraliyev et al., 2009; Chou and Voit, 2009; Dai and Lai, 2010; Sun et al., 2012; Hussain et al., 2015; Cedersund et al., 2016
Differential evolution (DE)	Derivative free method, linearly combining randomly selected candidate solutions to obtain iterative improvements
	Srinivas and Rangaiah, 2007; Liu and Wang, 2009; Chong et al., 2012, 2014; Sun et al., 2012; Teijeiro et al., 2017
Scatter search (SS)	Often combined with tabu search, it is local search with temporarily accepting worse solutions and avoiding already visited regions
	Rodriguez-Fernandez et al., 2006a; Villaverde et al., 2012; Cedersund et al., 2016; Penas et al., 2017; Remlia et al., 2017
Particle swarm optimiz. (PSO)	Derivative free method, moving particles (i.e., samples or candidate solutions) toward better solution
	Besozzi et al., 2009; Abdullah et al., 2013c; Sun et al., 2014; Cazzaniga et al., 2015; Nobile et al., 2016; Tangherloni et al., 2016

The cuckoo search utilizes random sub-populations which can be discarded to improve the solution (Rakhshania et al., 2016). The optimization programs include non-linear simplex method (Cazzaniga et al., 2015), non-linear programming (NLP) (Moles et al., 2003; Zhan and Yeung, 2011; Sun et al., 2012; Rodriguez-Fernandez et al., 2013), semi-definite programming (Kuepfer et al., 2007; Rumschinski et al., 2010), and quadratic programming (Gupta, 2013). The Nelder-Mead method (also known as the downhill simplex method) maintains a simplex of the test points which evolve until the data fit is found (Abdullah et al., 2013a). The quantifier elimination (QE) is used to simplify the constrained optimization problems (Anai et al., 2006). Other examples of the nature inspired algorithms include the firefly algorithm (FA) (Abdullah et al., 2013a,b) and the artificial bee colony (ABC) algorithm (Chong et al., 2014). Neural networks are becoming popular especially due to multi-layer deep learning methods. Other tasks encountered in traditional neural networks involve training, overfitting, smoothing, and the mean value approximations (Matsubara et al., 2006; Chou and Voit, 2009; Ali et al., 2015; Berrones et al., 2016). The parallel implementation of the scatter search for large-scale systems is devised in Villaverde et al. (2012) and Penas et al. (2017).

The benefits of individual optimization methods can be utilized by adaptively combining different algorithms. For instance, the DE is combined with the tabu search in Srinath and Gunawan (2010), and another hybrid DE method is considered in Liu and Wang (2008b). The genetic programming and the PSO are combined in Nobile et al. (2013), the multi-swarm PSO is considered in Nobile et al. (2012), and the fuzzy logic based PSO is developed in Nobile et al. (2015), Nobile et al. (2016), and Nobile et al. (2018a). The regularization, pruning and the continuous genetic algorithm (CGA) are combined in Liu et al. (2012).

Machine learning (MLR) methods can be very effective provided that there is enough training data drawn from some fixed distribution (Pan and Yang, 2010). If there are not enough labeled data, or the generating distribution changes in time, it may be better to employ transfer learning (TLR) methods which exploit data from multiple domains (Pan and Yang, 2010; Weiss et al., 2016; Azab et al., 2018). A primer on the MLR and the deep learning (DLR) methods for biological networks is provided in Camacho et al. (2018).

**Figure 3:**
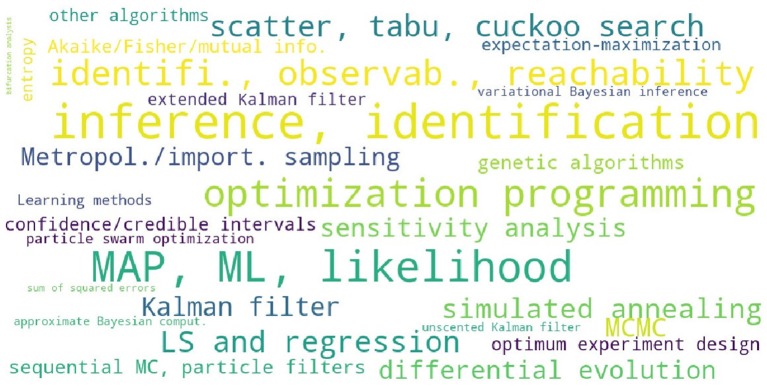
A word cloud visualizing the levels of interest in different parameter estimation methods and tasks for models of BRNs.

The survey of 5 estimation tasks and 23 estimation methods for BRNs identified in the references listed at the end of this paper is provided in Table S2. This table is summarized in Table 7 for convenience, and the corresponding word cloud is shown in Figure 3. Other tasks related to the parameter estimation which are commonly used in literature are the model identifiability, the parameter observability, and the reachability analysis. The information theoretic measures are assumed relatively often as an alternative to the probabilistic measures to define the rigorous inference problems. The parameter identification by model fitting appears to be the most common strategy in literature. The Bayesian analysis which accounts for the prior distribution of parameters is often performed numerically by adopting the MCMC and other statisticali sampling methods.

**Table 7:**
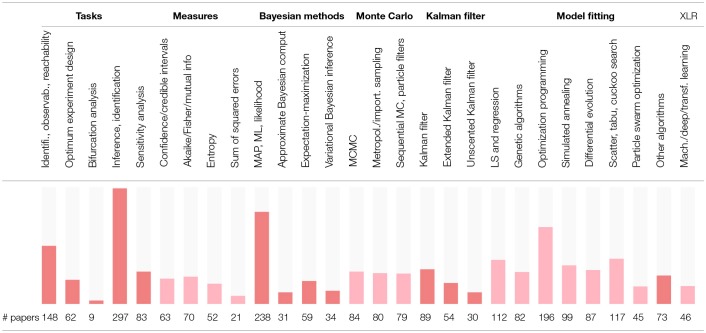
The coverage of the parameter estimation methods for BRNs.

In order to visualize a timeline of interest in different parameter estimation methods, Table 8 contains the numbers of cited papers concerning the specific estimation methods and tasks in given years. As for the methods in Table 3, we can observe that the general interest appears to have peaked in 2014, although the considerable interest has remained strong over the past decade. This indicates that the parameter estimation strategies are closely related to the modeling strategies as discussed previously.

**Table 8:**
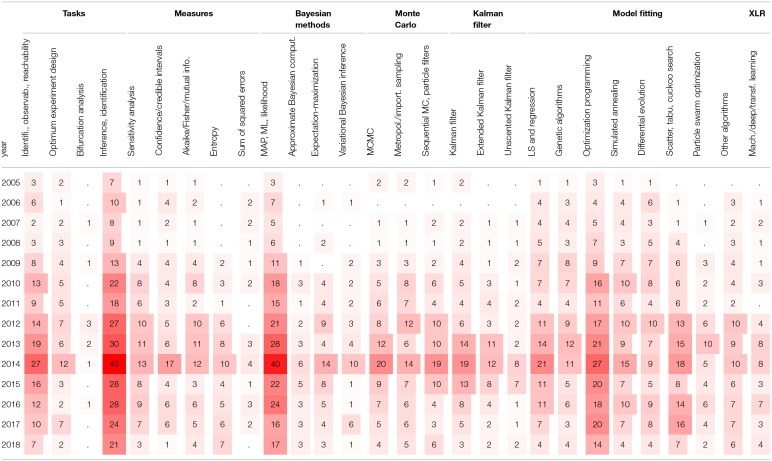
The number of papers concerning the estimation tasks and methods for BRNs in given years.

## 5. Choices of Models and Methods for Inferences in BRNs

We now evaluate what BRN models are preferred with the different parameter estimation strategies, and also explore what parameter estimation methods are assumed in different parameter estimation tasks. The models and the estimation tasks and methods are the same as those considered in Tables 2, 7, respectively.

Table 9 shows the number of papers concerning given BRN models and given estimation strategies. The paper counts were adjusted to exclude papers which were deemed to only marginally consider a given combination of the BRN model and the estimation task or method. In particular, the papers containing <5 occurrences of the search keywords for either a given model, task or method were excluded. We can observe that the parameter inference tasks have been considered for all the BRN models, however, some models have been investigated much more than the others. The most popular models for the parameter inferences and other related tasks are the models involving differential equations, Markov processes, and state space representations. The second most popular group of models considered for the parameter estimation include the S-system and polynomial models, and the moment closure and the LNA models.

**Table 9:**
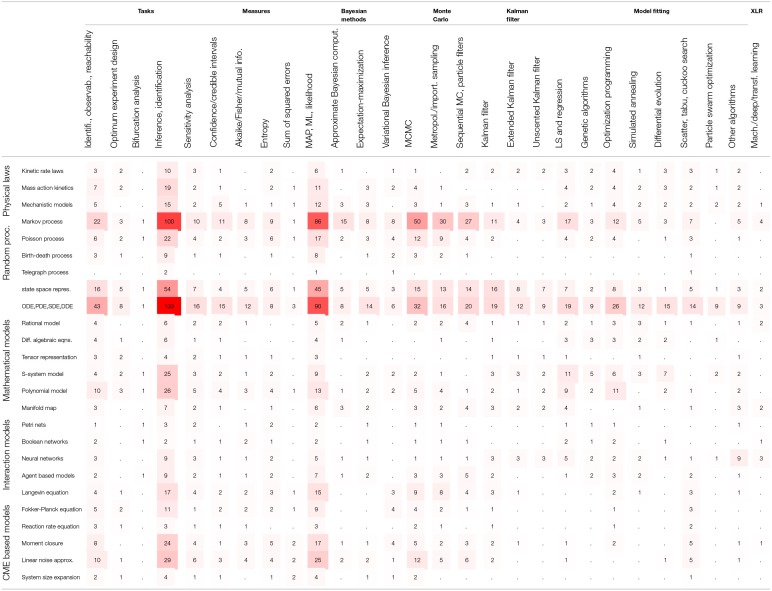
The adjusted number of papers concerning given estimation methods and given BRN models.

The sensitivity analysis using the information theoretic measures and evaluation of the confidence and credible intervals have been considered for most BRN models. The sensitivity analysis has somewhat similar use of models as the parameter inference, except the level of interest in the former is about ten times smaller. Moreover, the sensitivity analysis is often combined with the bifurcation analysis, so the latter may not be referred to explicitly in many papers. The optimum experiment design has been assumed for several models, but there seems to be no clear model preference. The sum of squares measure is likely quite underestimated in Table 9, since it is often assumed without being explicitly referred to.

The probabilistic MAP and ML measures have been assumed for all model types. In many cases, the corresponding inference tasks involve the prior and posterior distributions and probabilities, and the parameter likelihoods. The variational Bayesian and the ABC methods are mostly used with the Markov processes, since this is where they were originally developed for whereas the Markov processes can be derived from differential equations. The EM method is mostly used with the differential equations. The MC based sampling methods including particle filters are important for practical implementation of the Bayesian inference strategies. However, these methods seem to be rarely used with less popular BRN models. Similar comments can be made about the Kalman filtering, the LS regression, and most of the data fitting methods considered. The PSO method has been mainly considered with the models involving differential equations, and to some extent also with several other models. There are several BRN models which are not assumed with other inference algorithms, such as neural networks.

The statistical learning methods including MLR, DLR and TLR are still used sporadically compared to the other methods discussed so far. Consequently, it is still difficult to identify which BRN models in literature are preferred for statistical learning. The statistical learning requires enough training data as well as some level of time invariance in order to find generalized descriptions of systems, and to make predictions from the data. However, as the interest in applications of the MLR techniques continues to grow, and the efficiency of learning from data improves, it will also affect suitability of the MLR techniques for use with the different BRN models.

Another interesting viewpoint is to evaluate what inference methods are used for different inference tasks. The numbers of papers for given combinations of the inference tasks and the inference methods are provided in Table 10. With one exception, there is at least one paper for each such combination, however, the level of interest varies considerably. In particular, the largest number of papers for all the inference tasks considered assume the Bayesian analysis and the methods for the model fitting to data. On the other hand, the sum of squared errors, the UKF, and the PSO methods are generally the least assumed. As discussed previously, the sum of squared errors is used often, but rarely mentioned explicitly whereas the UKF and the PSO methods are usually rather difficult to implement.

**Table 10:**
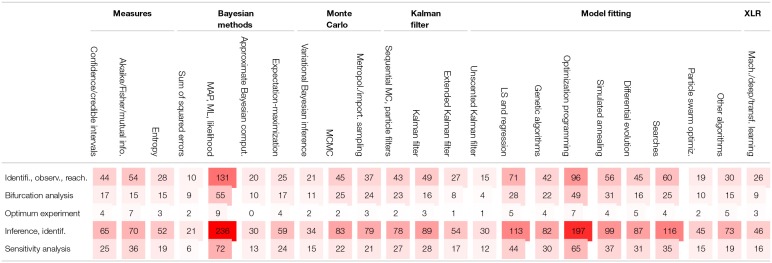
The number of papers concerning given tasks and given estimation methods for BRNs.

Assuming Table 10, we can compare the levels of interest for two or more methods and the given inference task. For example, the EM and the MCMC methods are used equally often for the sensitivity analysis whereas the MCMC method is preferred over the EM method for the identifiability task. The LS and the regression methods seem to be always preferred over Kalman filtering due to its implementation complexity. Interestingly, the MLR methods appear to be considered more often than the ABC, the variational Bayesian inference, the UKF, and the PSO methods, but comparably often to the EKF.

### 5.1. Future Research Directions

Tables 9, 10 together with the data in Tables 2, 7 can be used as guidelines to define new research problems which have not been sufficiently investigated in literature. We can separate the models, tasks and methods into the groups according to their levels of interest. Due to sparsity of data in Table 9, it is easier to enumerate the problems which have already been well-investigated in the literature. Such cases are highlighted in Table 9, and they include:

- The identification and inference tasks with the Markov processes, state space representations, differential equations, polynomial function, S-system, Langevin and Fokker-Planck equations, and the CME approximation models;
- Most of the inference methods with the Markov processes, state space representations, and the differential equation models;
- Some inference methods assuming the Poisson process, S-system, polynomial function, Langevin equation, and the CME approximation models;
- The Bayesian methods with the MAP and ML inferences with most of the models considered;
- The LS regression and the optimization programming mainly with the Markov processes, state space representations, differential equations, S-system and the polynomial models; and
- The search methods with the Markov processes, state space representations, differential equations, and the CME approximation models.

The bifurcation analysis appears to be the least considered task for all models. However, in many papers, the bifurcation analysis may not be referred to explicitly as it is performed as part of the sensitivity analysis. Similar comments can be made about the sum of squared errors. From Table 9, we observe that also machine learning methods have been considered sporadically and only for some BRN models to solve the inference problems. Comparing machine learning methods with the conventional methods of statistical inference may be one of the most interesting research avenues in near future. It is likely that machine learning is more beneficial for some models, depending on the availability of observations and training data. In addition, we can observe from Table 10 that the optimum experiment design did not receive as much attention in literature as other inference tasks.

There are likely other research opportunities which are not immediately apparent from the tables in previous sections. For instance, the minimum mean square error (MMSE) estimator is only discussed in the reference (Koeppl et al., 2012). Since the estimation errors may have different distributions depending on the BRN model considered, the generalized linear regression (GLR) can be assumed as a simple to implement, universal and yet powerful statistical learning technique. The GLR method has not been investigated for the inferences in BRNs. It is also useful to estimate other quantities in addition to inferring the parameter values. For example, the distributions of species counts are estimated in Atitey et al. (2018b). Knowledge of the parameter distributions greatly affects the available choices of estimators and their performance. Another unexplored strategy is the compressive sensing (CS) which exploits the sparsity in parameter spaces. Among machine learning methods, the transfer learning has not been used for inferences in BRNs in order to exploit the increasing production of omics data (Weiss et al., 2016).

Furthermore, the vast majority of inference problems in literature assume the well-stirred models of BRNs with the reactions dependent solely on the species concentrations, but not on the species spatial distributions. Assuming the spatially resolved models of BRNs with the diffusion and other phenomena of the molecular transport through complex fluids is much more realistic. Such models are usually described by the RDME (Lötstedt, 2018). Moreover, in many BRNs, the reaction rates are time varying. The inferences of time varying parameters in BRN models have not been explicitly considered in literature.

Most inference problems in literature assume simple models of measurements, such as obtaining the noisy concentrations of species at discrete time instances. In order to increase the sensitivity of measurements, the observations are often accumulated in time (Folia and Rattray, 2018). The transformations, such as the time integration of measurements must be incorporated into the BRN models when devising the interference strategies. Since the measurements may affect the biological processes, the number and duration of the measurements should be minimized in space and in time. In addition, the measurement noise is often (but not always) assumed to be independent of the species concentrations and Gaussian distributed. In realistic *in vivo* and *in vitro* experiments, the measurement noises are correlated in time and with other measurements, and also dependent on the reaction rates and the species concentrations. It would be very useful to report the statistical properties of measurements from the different laboratory experiments. Having such statistical description of measurements can considerably improve the efficiency and accuracy of the inference methods in BRNs.

More generally, the performance of various inference strategies is greatly dependent on the structure, parameter values and the initial state of the BRN considered. These aspects were considered mostly to optimize the data fitting methods, but much less for the other inference methods. There is a trade-off in mechanistically employing the universal inference methods, and adopting these methods to specific scenarios of the BRNs. The latter approach may improve the performance and efficiency of the parameter inference at the cost of increased implementation complexity. More research is needed to jointly explore the model simplification strategies and the parameter estimation strategies as in Eghtesadi and Mcauley (2014). However, it is always important to test and validate all the inference algorithms devised. In some papers, the inference algorithms are tested on multiple data sets, but a general methodology for testing and validating the inference algorithms for BRNs have not been presented in literature.

Many papers on the inferences in BRNs are concerned with the implementation aspects rather than the concepts. It would be useful to separate the inference concepts and strategies form their implementation. For example, the Bayesian inference can be implemented using the stochastic sampling, the ABC, the variational inference, the EM and several other methods.

Finally, let's not forget that the ultimate goal of performing the statistical inferences in BRNs is to improve our understanding of the *in vivo* and *in vitro* biological systems and phenomena. It is primarily dependent on having the sufficiently accurate models of these systems including knowing the values of their parameters. As the experimental techniques improve, the new data from the experiments will likely stimulate the developments of new biological models, and thus, there will also be the need for new inference methods and strategies.

## 6. Conclusions

The aim of this review paper was to explore how various inference tasks and methods are used with different models of BRNs. The key concepts of modeling and the parameter inferences for BRNs were discussed. The dependency between tasks, methods and models were captured in tables containing the paper counts. More detailed information is provided in Supplementary Tables including the links for selected papers to their citations in Google Scholar.

The common models and inference tasks and methods for BRNs were identified by text mining the cited references. The text mining was partly automated using text processing scripts. Such automation is indispensable when dealing with a large number of references as is the case in this paper. For convenience, the identified models and methods were presented under several loosely defined categories. The most common models of BRNs in literature are the mass action kinetics, Markov processes, state space representations, and differential equations. Somewhat less common, but still popular models include the kinetic rate law, mechanistic models, Poisson processes, polynomial and rational functions, the S-system model, the Langevin equation, and the CME based approximation models.

Several previously published review papers concerning the inferences in BRNs were listed. The relevant graduate research theses from the past decade were also outlined, since they tend to contain comprehensive literature surveys and tutorial style explanations. We observed that the most common inference tasks are concerned with the model identifiability, the parameter inference and the sensitivity analysis. The most common inference methods are the Bayesian analysis using the MAP and ML estimators, the MC sampling techniques, the LS regression, and the evolutionary algorithms for data fitting including the optimization programming, the simulated annealing, and the scatter and other searches.

In the last part of the paper, the levels of interest in different inference tasks and methods for given BRN models were assessed. This allowed us to identify the inference problems for BRNs which were less explored in the literature previously. Our study revealed that the interest in the inference problems in BRNs peaked in 2014. This may indicate that development of the traditional statistical methods has saturated, and the current focus is more on their efficient implementation, especially to process the massive amounts of data. The new developments will likely be driven by the machine learning methods and the continuing progress experimental techniques. The results presented in this review can be used to develop a coherent theory comprising the models and methods for the statistical inferences in BRNs.

## Author Contributions

All authors: substantial contributions to the conception and design of the work. PL: drafting the work. All authors: revising the work critically for important intellectual content, final approval of the version to be published, and agreement to be accountable for all aspects of the work in ensuring that questions related to the accuracy or integrity of any part of the work are appropriately investigated and resolved.

### Conflict of Interest Statement

The authors declare that the research was conducted in the absence of any commercial or financial relationships that could be construed as a potential conflict of interest.

## Abbreviations

ABC: approximate Bayesian computation, artificial bee colony
ABM: agent based model
AR: alternating regression
CCA: canonical correlation analysis
CDIS: conditional density importance sampling
CGA: continuous genetic algorithm
CLE: chemical Langevin equation
CME: chemical master equation
CRO: chemical reaction optimization
CS: compressive sensing
CTMC: continuous time Markov chain
CTMP: continuous time Markov process
DE: differential evolution
DLR: deep learning
EKF: extended Kalman filter
EM: expectation-maximization
EP: expectation propagation
FA: firefly algorithm
FDM: finite differences method
GLR: generalized linear regression
GLR: generalized linear regression
GP: genetic programming
HDL: hardware description language
KF: Kalman filter
LFM: linear fractional model
LNA: linear noise approximation
LS: least squares
MAP: maximum a posterior
MC: Monte Carlo
MCEM: MC expectation-maximization
MCMC: MC Markov Chain
MES: maximum entropy sampling
ML: maximum likelihood
MLR: machine learning
MM: method of moments
MMSE: minimum mean square error
NLP: non-linear programming
NLR: natural language processing narrative literature review
NLSQ: non-linear least squares
ODE: ordinary differential equation
PDF: portable document format, probability density function
PMC: population Monte Carlo
PSO: particle swarm optimization
QE: quasi-equilibrium
QSS: quasi-steady state
RDME: reaction-diffusion master equation
RRE: reaction rate equation
SA: simulated annealing
SMC: sequential Monte Carlo
SMCMC: sequential Markov chain Monte Carlo
SS: scatter search
SSE: sum of squared errors
SLR: system size expansion systematic literature review
TLR: transfer learning
UKF: unscented Kalman filter.
