# Supplementary material for: Comprehensive Review of Models and Methods for Inferences in Bio-Chemical Reaction Networks
Source: Front Genet. 2019 Jun 14;10:549. doi: 10.3389/fgene.2019.00549 (PMC6588029; doi:10.3389/fgene.2019.00549)
Supplement: Supplementary file 1 [file Data_Sheet_1.PDF]

# Supplementary Material

## SUPPLEMENTARY TABLES

THE COVERAGE OF MODELING STRATEGIES FOR BRNS. . . . . . . . . . . . . . . . . . . . . . . . . . . . . . . . . . 50
THE COVERAGE OF PARAMETER ESTIMATION STRATEGIES FOR BRNS. . . . . . . . . . . . . . . . . . . 56
THE REFERENCES WITH CITATION LINKS TO GOOGLE SCHOLAR. . . . . . . . . . . . . . . . . . . . . . . . 62

Table S1 lists all the references cited in the main text indicating how many times the given model was mentioned in each reference.

Table S2 lists all the references cited in the main text indicating how many times the given task or method was mentioned in each reference.

Table S3 provides the links to citing papers on Google Scholar for the selected references.

**Table S1.** Coverage of modeling strategies of BRNs.

| | Physical laws | | | Random processes | | | | | Mathematical models | | | | | | | Interaction models | | | | | CME based models | | | | |
|---|---|---|---|---|---|---|---|---|---|---|---|---|---|---|---|---|---|---|---|---|---|---|---|---|---|
| Reference | kinetic rate laws | mass action kinetics | mechanistic models | Markov process | Poisson process | birth-death process | telegraph process | state space representation | ODEs, PDEs, SDEs, DDEs | rational model | differential algebraic eqns. | tensor representation | S-system model | polynomial model | manifold map | Petri nets | Boolean networks | neural networks | agent based models | Langevin equation | Fokker-Planck equation | reaction rate equation | moment closure | linear noise approximation | system size expansion |
| Abdullah et al. (2013c) | . | . | . | . | . | . | . | 1 | 1 | 1 | 1 | . | . | . | . | . | . | . | . | . | . | . | . | . | . |
| Abdullah et al. (2013b) | . | . | . | . | . | . | . | 2 | 4 | 4 | . | . | . | 1 | . | . | . | . | 1 | . | . | . | . | . | . |
| Abdullah et al. (2013a) | . | . | . | . | . | . | . | . | 7 | 1 | . | . | . | . | . | . | . | . | 3 | . | . | . | . | . | . |
| Alberton et al. (2013) | . | . | 1 | . | . | . | . | . | . | . | . | . | . | . | . | . | . | 1 | . | . | . | . | . | . | . |
| Ale et al. (2013) | 1 | . | . | . | . | . | . | . | 10 | . | . | . | . | 3 | . | . | . | . | . | . | . | . | 7 | 15 | . |
| Ali et al. (2015) | . | . | 2 | . | . | . | . | 4 | 1 | . | . | . | . | 3 | . | . | . | 21 | . | . | . | . | . | . | . |
| Amrein and Künsch (2012) | . | 2 | . | 33 | 3 | . | . | 9 | 1 | . | . | . | . | . | . | . | . | . | . | . | . | . | . | . | . |
| Anai et al. (2006) | . | . | . | . | . | . | . | . | 1 | . | . | . | . | 2 | . | . | . | . | . | . | . | . | . | . | . |
| Andreychenko et al. (2011) | . | . | . | 21 | . | . | . | 20 | 7 | . | . | . | . | . | . | . | . | . | . | . | . | . | . | . | . |
| Andreychenko et al. (2012) | . | . | . | 10 | . | . | . | 13 | 6 | . | . | . | . | . | . | . | . | . | . | . | . | . | . | 2 | . |
| Andreychenko et al. (2015) | . | . | . | 10 | . | . | . | 10 | 3 | . | . | . | . | 1 | . | . | . | . | . | . | . | . | 29 | . | . |
| Andrieu et al. (2010) | . | . | 2 | 99 | 3 | . | . | 49 | . | . | . | . | . | . | 1 | . | . | . | . | 2 | . | . | . | . | . |
| Angius and Horváth (2011) | . | 9 | . | 4 | . | . | . | 5 | 9 | . | . | . | . | . | . | . | . | . | 9 | . | . | . | . | . | . |
| Arnold et al. (2014) | . | . | 1 | 3 | . | . | . | . | 5 | . | . | . | . | . | . | . | . | . | . | . | . | . | . | . | . |
| Ashyraliyev et al. (2009) | . | . | . | 5 | . | . | . | 1 | 1 | . | 10 | . | . | . | . | . | . | . | . | . | . | . | . | . | . |
| Atitey et al. (2018b) | . | . | . | 3 | . | . | . | . | . | . | . | 1 | . | . | . | . | . | . | . | . | . | . | . | . | . |
| Atitey et al. (2018a) | . | . | . | . | . | . | . | . | . | 1 | . | . | . | . | . | . | . | . | . | . | . | . | . | . | . |
| Atitey et al. (2019) | . | . | . | . | 2 | . | . | . | 1 | 1 | . | . | . | . | . | . | . | . | . | . | . | . | . | . | . |
| Azab et al. (2018) | . | 1 | . | . | . | . | . | . | . | . | . | . | . | . | . | . | . | . | . | . | . | . | . | . | . |
| Babtie and Stumpf (2017) | . | . | 7 | 1 | . | . | . | . | 4 | 1 | . | . | . | . | 1 | . | . | . | . | . | . | . | . | . | . |
| Backenköhler et al. (2016) | . | 6 | . | 3 | . | . | . | 4 | 1 | . | . | . | . | 2 | . | . | . | . | . | . | . | . | 7 | . | . |
| Backenköhler et al. (2018) | . | 7 | . | 3 | 1 | 1 | . | 4 | 1 | 1 | . | . | . | 2 | . | . | . | . | . | . | . | . | 8 | . | . |
| Baker et al. (133, 2010) | 2 | . | . | . | . | . | . | . | 2 | 1 | 1 | . | . | . | . | . | . | . | . | . | . | . | . | . | . |
| Baker et al. (2011) | 8 | . | 1 | . | . | . | . | 6 | 4 | . | . | . | . | . | . | . | . | . | . | . | . | . | . | . | . |
| Baker et al. (2013) | 5 | . | 2 | 4 | . | . | . | 14 | 10 | . | . | . | . | . | . | . | . | . | . | . | . | . | . | . | . |
| Baker et al. (2015) | 4 | . | 2 | . | . | . | . | 10 | 8 | 1 | . | . | . | . | . | . | . | . | . | . | . | . | . | . | . |
| Banga and Canto (2008) | . | . | 2 | . | . | . | . | 1 | 3 | . | 1 | . | . | . | . | . | . | . | . | . | . | . | . | . | . |
| Barnes et al. (2011) | . | . | 2 | 2 | . | . | . | 1 | 1 | . | . | . | . | . | . | . | . | . | . | . | . | . | . | . | . |
| Bayer et al. (2016) | . | 2 | . | 20 | . | . | . | 7 | 19 | . | . | . | . | 2 | 3 | . | . | . | . | 1 | 3 | . | 1 | . | . |
| Berrones et al. (2016) | . | . | . | 2 | . | . | . | . | . | . | . | . | 3 | . | . | . | . | 2 | . | . | . | . | . | . | . |
| Besozzi et al. (2009) | . | . | . | . | . | . | . | . | 3 | . | . | . | . | . | . | . | . | . | 4 | . | . | . | . | . | . |
| Bhaskar et al. (2010) | . | . | . | 3 | . | . | . | . | 2 | . | . | . | . | . | . | . | . | . | . | . | . | . | . | . | . |
| Blei et al. (2017) | . | . | . | 14 | 3 | . | . | . | . | . | . | 1 | . | . | 1 | . | . | 1 | 1 | 1 | . | . | . | . | . |
| Bogomolov et al. (2015) | 1 | 1 | . | 6 | . | . | . | 2 | 10 | . | . | . | . | 1 | . | . | . | . | . | . | . | . | 27 | 1 | . |
| Bouraoui et al. (2015) | . | . | . | . | . | . | . | 1 | . | . | . | . | . | . | . | . | . | . | . | . | . | . | . | . | . |
| Farza et al. (2016) | . | . | . | . | . | . | . | 1 | 4 | . | . | . | . | . | . | . | . | . | . | . | . | . | . | . | . |
| Boys et al. (2008) | 3 | 1 | . | 7 | 6 | . | . | 2 | . | . | . | . | . | . | . | . | . | . | . | . | . | . | . | . | . |
| Brim et al. (2013) | . | 7 | . | 13 | 9 | 2 | . | 25 | 12 | . | . | . | . | 4 | . | . | . | . | . | . | . | . | 2 | . | . |
| Bronstein et al. (2015) | . | 1 | . | 11 | 2 | . | . | 2 | 13 | . | . | . | . | . | 1 | . | . | . | . | 2 | 4 | . | 2 | 18 | . |
| Bronstein and Koeppl (2018) | . | . | . | 8 | 46 | . | . | 3 | 2 | . | . | . | . | 4 | . | . | . | . | . | . | 5 | 1 | 62 | 2 | . |
| Busetto and Buhmann (2009) | 1 | 2 | . | 8 | . | . | . | 4 | 2 | . | . | 2 | . | . | . | . | . | . | . | 2 | 2 | . | . | . | . |
| Camacho et al. (2018) | . | . | 1 | . | . | . | . | . | 1 | 2 | . | . | . | . | . | . | . | 23 | . | . | . | . | . | . | . |
| Balsa-Canto et al. (2008) | . | . | . | . | . | . | . | . | 1 | . | . | . | . | . | . | . | . | . | . | . | . | . | . | . | . |
| Carmi et al. (2013) | . | . | . | 21 | 4 | . | . | 2 | 3 | . | . | . | . | . | . | . | . | . | 63 | . | . | . | . | . | . |
| Cazzaniga et al. (2015) | . | . | 1 | . | . | . | . | . | 1 | . | . | . | . | . | . | . | . | . | . | . | . | . | . | . | . |
| Cedersund et al. (2016) | . | . | 1 | . | . | . | . | 1 | 8 | 1 | . | . | . | . | . | . | . | 3 | 1 | . | . | . | . | . | . |
| Česka et al. (2014) | . | 6 | . | 10 | . | 2 | . | 19 | 13 | . | . | . | . | 2 | . | . | . | . | . | . | . | . | 1 | . | . |
| Češka et al. (2017) | . | 2 | . | 13 | 5 | 2 | . | 3 | 8 | . | . | . | . | 28 | . | . | 1 | . | . | . | . | . | . | . | . |
| Chen et al. (2017) | . | . | . | . | . | . | . | . | 1 | . | . | . | . | . | . | . | . | . | . | . | . | . | 2 | . | . |

**Table S1.** Coverage of modeling strategies of BRNs. (cont.)

| | Physical laws | | | Random processes | | | | | Mathematical models | | | | | | Interaction models | | | | CME based models | | | | | | |
|---|---|---|---|---|---|---|---|---|---|---|---|---|---|---|---|---|---|---|---|---|---|---|---|---|---|
| Reference | kinetic rate laws | mass action kinetics | mechanistic models | Markov process | Poisson process | birth-death process | telegraph process | state space representation | ODEs, PDEs, SDEs, DDEs | rational model | differential algebraic eqns. | tensor representation | S-system model | polynomial model | manifold map | Petri nets | Boolean networks | neural networks | agent based models | Langevin equation | Fokker-Planck equation | reaction rate equation | moment closure | linear noise approximation | system size expansion |
| Chevaliera and Samadb (2011) | . | . | . | 3 | . | . | . | . | 1 | 3 | . | . | . | 4 | . | . | . | . | . | . | . | 1 | 25 | . | . |
| Chong et al. (2012) | . | . | . | . | . | . | . | . | 4 | . | . | . | 1 | . | . | . | . | 1 | . | . | . | . | . | . | . |
| Chong et al. (2014) | . | . | . | . | . | . | . | . | 7 | . | . | . | . | . | . | . | . | . | . | . | . | . | . | . | . |
| Chou et al. (2006) | 1 | . | . | . | . | . | . | . | . | . | . | . | 23 | . | . | . | . | 1 | . | . | . | . | . | . | . |
| Chou and Voit (2009) | 9 | 6 | 10 | . | . | . | . | . | 5 | . | . | . | 82 | . | 2 | 1 | 2 | 6 | 1 | . | . | . | . | . | . |
| Cseke et al. (2016) | . | . | . | 22 | . | . | . | 4 | 17 | . | . | . | . | 2 | . | . | . | . | . | 6 | 5 | . | 12 | . | . |
| Dai and Lai (2010) | . | . | . | . | . | . | . | . | 7 | 1 | . | . | . | . | 1 | . | . | . | . | . | . | . | . | . | . |
| Daigle et al. (2012) | . | 2 | 5 | 2 | 47 | 17 | . | . | . | . | . | . | . | . | . | . | . | . | . | . | . | . | . | . | . |
| Dargatz (2010) | . | 1 | . | 195 | 25 | . | . | 83 | 144 | . | . | . | . | 9 | 1 | . | . | . | 1 | 33 | 13 | . | . | 2 | . |
| Dattner (2015) | . | . | . | . | . | . | . | 1 | 28 | . | . | . | . | 15 | . | . | . | . | 1 | . | . | . | . | . | . |
| Deng and Tian (2014) | . | . | 1 | 1 | . | . | . | . | 3 | . | 10 | . | 2 | 1 | . | . | . | . | . | . | . | . | . | 1 | . |
| Dey et al. (2018) | . | 1 | . | . | . | . | . | 1 | . | . | . | . | . | . | . | . | . | . | . | 8 | 1 | . | . | . | . |
| Dinh and Sidje (2017) | . | . | . | 2 | . | . | . | 9 | 18 | . | . | 1 | . | . | . | . | . | . | . | . | . | . | . | . | . |
| Dochain (2003) | . | . | . | . | . | . | . | 2 | . | . | . | . | . | 1 | . | . | . | . | . | . | . | . | . | . | . |
| Drovandi et al. (2016) | . | 1 | . | 36 | 4 | . | . | 2 | . | . | . | . | . | . | . | . | . | . | . | . | . | . | . | 1 | . |
| Eghtesadi and Mcauley (2014) | . | . | 3 | . | . | . | . | . | . | . | 2 | . | . | . | . | . | . | . | 1 | . | . | . | . | . | . |
| Eisenberg and Hayashi (2014) | . | . | . | . | . | . | . | 1 | 10 | . | . | . | . | . | . | . | . | . | . | . | . | . | . | . | . |
| Engl et al. (2009) | 20 | 7 | . | . | . | . | . | 1 | 44 | . | . | . | 6 | . | 3 | . | 1 | . | . | . | . | . | . | . | . |
| Erguler and Stumpf (2011) | . | . | 4 | . | . | . | . | . | 8 | . | . | . | . | . | . | . | . | . | . | . | . | . | . | . | . |
| Fages et al. (2015) | 1 | 4 | . | 1 | . | . | . | . | 91 | . | . | . | . | 8 | . | 8 | . | . | . | . | . | . | . | . | . |
| Famili et al. (2005) | 6 | 2 | 2 | . | . | . | . | . | . | . | . | . | . | . | . | . | . | . | . | . | . | . | . | . | . |
| Farina et al. (2006) | . | 9 | . | . | . | . | . | 1 | . | 1 | . | . | . | 1 | . | . | . | . | . | . | . | . | . | . | . |
| Fearnhead and Prangle (2012) | . | . | 1 | 39 | 1 | . | . | 3 | . | . | . | . | . | 4 | . | . | . | 4 | . | . | . | . | . | . | . |
| Fearnhead et al. (2014) | . | . | . | 7 | 3 | . | . | 4 | 86 | . | . | . | . | . | . | . | . | . | . | . | . | . | . | 125 | . |
| Rodriguez-Fernandez et al. (2006b) | . | . | . | . | . | . | . | 2 | 7 | . | 2 | . | . | . | . | . | . | . | . | . | . | . | . | . | . |
| Rodriguez-Fernandez et al. (2006a) | . | . | 1 | . | . | . | . | . | 3 | . | 2 | . | . | . | . | . | . | . | . | . | . | . | . | . | . |
| Rodriguez-Fernandez et al. (2013) | . | . | . | 1 | . | . | . | . | 2 | . | 6 | . | . | . | . | . | . | . | . | . | . | . | . | . | . |
| Fey et al. (2008) | . | 2 | . | . | . | . | . | 1 | 1 | . | . | . | . | . | 1 | . | . | . | . | . | . | . | . | . | . |
| Fey and Bullinger (2010) | . | 4 | . | . | . | . | . | . | 1 | . | . | . | . | 18 | . | . | . | . | . | . | . | . | . | . | . |
| Flassig (2014) | 2 | . | 5 | 5 | . | . | . | 1 | 71 | 16 | . | . | . | 4 | 1 | . | 7 | . | . | . | . | . | . | . | . |
| Folia and Rattray (2018) | . | . | . | 8 | . | . | . | . | 31 | . | . | . | . | . | . | . | . | . | . | . | . | . | . | 29 | . |
| Fröhlich et al. (2014) | . | . | . | 7 | . | . | . | . | 10 | . | . | . | . | . | . | . | . | . | . | . | . | . | . | . | . |
| Fröhlich et al. (2016) | . | . | 6 | 3 | . | . | . | . | 9 | . | . | 3 | . | . | 1 | . | . | . | 2 | 1 | 1 | 2 | 5 | 41 | 50 |
| Fröhlich et al. (2017) | 1 | 1 | 11 | 1 | . | . | . | . | 16 | 3 | 1 | 1 | . | 1 | 3 | . | . | . | . | 2 | . | . | . | . | . |
| Gábor and Banga (2014) | . | . | . | . | . | . | . | . | 3 | . | . | . | . | . | . | . | . | . | . | . | . | . | . | . | . |
| Gábor et al. (2017) | . | . | 2 | . | . | . | . | . | 3 | 1 | . | . | . | . | . | . | . | . | . | . | . | . | . | . | . |
| Galagali (2016) | . | 11 | 1 | 46 | 3 | . | . | 7 | 20 | . | . | . | . | . | . | . | 2 | . | . | 1 | . | . | . | 1 | . |
| Geffen et al. (2008) | . | 1 | . | . | . | . | . | 2 | . | . | . | . | . | . | . | . | . | . | . | . | . | . | . | . | . |
| Gennemark and Wedelin (2007) | . | . | . | . | . | . | . | . | 20 | . | . | . | 10 | 3 | . | . | . | . | . | . | . | . | . | . | . |
| Ghusinga et al. (2017) | 1 | . | . | . | 1 | . | . | 1 | . | . | . | . | . | 5 | . | . | . | 1 | . | . | . | . | 19 | . | . |
| Gillespie and Golightly (2012) | . | 1 | . | 5 | 1 | . | . | . | 24 | . | . | . | . | . | . | . | . | . | . | . | . | . | 1 | . | . |
| Golightly and Wilkinson (2006) | 1 | 2 | . | 6 | 1 | . | . | . | 3 | . | . | . | . | . | . | . | . | . | . | . | 2 | . | . | . | . |
| Golightly and Wilkinson (2005) | 1 | 4 | . | 12 | 2 | . | . | . | 9 | . | . | . | . | . | . | . | . | . | . | 1 | 4 | . | . | . | . |
| Golightly and Wilkinson (2011) | 2 | 1 | 1 | 24 | 1 | . | . | 4 | 9 | . | . | . | . | . | . | . | . | . | . | 4 | 2 | . | . | . | . |
| Golightly et al. (2012) | . | . | . | 23 | 2 | . | . | 2 | 15 | . | . | . | . | . | . | . | . | . | . | 8 | . | . | . | 76 | . |
| Golightly et al. (2015) | 1 | 1 | . | 26 | 4 | . | . | 3 | 16 | . | . | . | . | . | . | . | . | . | . | 8 | . | . | . | 109 | . |
| Golightly and Kypraios (2017) | . | 1 | . | 20 | 1 | . | . | 3 | . | . | . | . | . | . | . | . | . | . | . | . | . | . | . | . | . |
| Golightly et al. (2019) | 1 | . | . | 17 | 29 | . | . | 2 | 4 | . | . | . | . | . | . | . | . | . | . | 7 | . | . | . | . | . |
| González et al. (2013) | . | . | . | . | . | . | . | 2 | 27 | . | . | . | 5 | . | 2 | . | . | 1 | . | 1 | . | . | . | . | . |

**Table S1.** Coverage of modeling strategies of BRNs. (cont.)

| | Physical laws | | | Random processes | | | | | | Mathematical models | | | | | | Interaction models | | | | | CME based models | | | | |
|---|---|---|---|---|---|---|---|---|---|---|---|---|---|---|---|---|---|---|---|---|---|---|---|---|---|
| Reference | kinetic rate laws | mass action kinetics | mechanistic models | Markov process | Poisson process | birth-death process | telegraph process | state space representation | ODEs, PDEs, SDEs, DDEs | rational model | differential algebraic eqns. | tensor representation | S-system model | polynomial model | manifold map | Petri nets | Boolean networks | neural networks | agent based models | Langevin equation | Fokker-Planck equation | reaction rate equation | moment closure | linear noise approximation | system size expansion |
| Gordon et al. (1993) | . | . | . | 1 | . | . | . | 9 | . | . | . | . | . | . | . | . | . | . | . | . | . | . | . | . | . |
| Guillén-Gosálbez et al. (2013) | 3 | . | 1 | . | . | . | . | . | 1 | . | . | . | 2 | . | . | . | . | . | . | . | . | . | . | . | . |
| Goutsias and Jenkinson (2013) | 1 | 2 | . | 146 | 41 | . | . | 14 | 1 | . | . | . | . | 2 | . | 9 | . | 20 | 10 | 12 | 3 | . | 11 | 26 | . |
| Gratie et al. (2013) | . | 5 | 4 | 8 | . | . | . | 1 | 55 | 1 | . | . | . | 1 | . | 2 | . | . | 1 | . | . | . | . | . | . |
| Gupta (2013) | . | 2 | 1 | 72 | 6 | . | . | 17 | 48 | . | . | . | . | 1 | . | . | . | . | . | . | . | . | . | . | . |
| Gupta and Rawlings (2014) | . | 2 | 1 | 17 | . | . | . | 11 | 4 | 1 | . | . | . | . | . | . | . | . | . | . | . | . | . | . | . |
| Hagen et al. (2013) | . | 1 | 4 | . | . | . | . | . | 6 | 4 | . | . | . | . | . | . | . | . | . | . | . | . | . | 2 | . |
| Hasenauer et al. (2010) | . | 2 | . | . | . | . | . | . | . | 1 | . | . | . | 9 | . | . | . | . | . | . | . | . | . | . | . |
| Hasenauer (2013) | 3 | 3 | 12 | 43 | 1 | 4 | . | 2 | 99 | 3 | . | . | . | 6 | 2 | . | . | . | 2 | 21 | 30 | 10 | 1 | . | . |
| Mustafa et al. (2013) | . | . | . | 1 | . | . | . | . | . | . | . | . | . | . | . | . | . | . | . | . | . | . | . | . | . |
| Th and Manini (2008) | . | . | . | 2 | . | . | . | 1 | 12 | . | . | . | . | . | . | . | . | . | . | . | . | . | . | . | . |
| Hussain et al. (2015) | . | . | . | 8 | . | . | . | 2 | 3 | 11 | . | . | 1 | 3 | . | 1 | . | . | 49 | . | . | . | . | . | . |
| Hussain (2016) | . | . | . | 16 | . | . | . | 8 | 13 | 20 | . | . | 4 | 2 | . | 2 | 2 | . | 45 | . | . | . | . | . | . |
| Iwata et al. (2014) | 1 | . | . | . | . | . | . | . | . | . | . | . | 34 | 1 | . | . | . | . | . | . | . | . | . | . | . |
| Jagiella et al. (2017) | . | . | 7 | 5 | . | . | . | . | 13 | 3 | . | . | . | . | . | . | . | . | 15 | . | . | . | . | . | . |
| Jang et al. (2016) | . | . | . | 2 | . | . | . | 2 | 1 | . | . | . | . | . | . | . | . | . | . | . | . | . | . | . | . |
| Jaqaman and Danuser (2006) | . | . | 2 | 3 | . | . | . | . | . | 1 | . | . | . | 3 | . | . | . | . | . | . | . | . | . | . | . |
| Ji and Brown (2009) | . | . | . | . | . | . | . | 2 | 19 | . | . | . | . | . | . | . | . | . | . | . | . | . | . | . | . |
| Jia et al. (2011) | 1 | . | . | . | . | . | . | . | 65 | . | . | . | 10 | 5 | . | . | . | 1 | . | . | . | . | . | . | . |
| Joshia et al. (2006) | 6 | . | . | . | . | . | . | . | . | . | . | . | . | . | . | . | . | . | . | . | . | . | . | . | . |
| Karnaukhov et al. (2007) | . | 1 | . | . | . | . | . | . | . | . | . | . | . | . | . | . | . | . | . | . | . | . | . | . | . |
| Karimi and Mcauley (2013) | . | . | . | 3 | . | . | . | 1 | 36 | . | . | . | . | 2 | . | . | . | . | . | . | . | . | . | . | . |
| Karimi and Mcauley (2014b) | . | . | . | 5 | . | . | . | 4 | 31 | . | . | . | . | 2 | . | . | . | . | . | . | 1 | . | . | . | . |
| Karimi and Mcauley (2014a) | . | . | . | 5 | . | . | . | . | 32 | . | . | . | . | 3 | . | . | . | . | . | . | 1 | . | . | . | . |
| Kimura et al. (2015) | . | . | 1 | 3 | 2 | . | . | . | 2 | . | . | . | . | 5 | . | . | . | . | . | . | . | . | . | . | . |
| Kleinstein et al. (2006) | . | 1 | . | . | . | . | . | . | 2 | 1 | . | . | . | . | . | . | . | . | . | . | . | . | . | . | . |
| Ko et al. (2009) | 1 | 2 | . | . | . | . | . | . | . | . | . | . | 5 | . | . | . | . | . | . | . | . | . | . | . | . |
| Koblents and Míguez (2011) | . | . | . | . | . | . | . | . | 2 | . | . | . | . | . | . | . | . | . | . | . | . | . | . | . | . |
| Koblents and Míguez (2014) | . | . | . | 29 | 1 | . | . | 4 | . | . | . | . | . | . | . | . | . | . | . | 1 | . | . | 2 | . | . |
| **?** | . | 1 | . | 18 | 1 | . | . | 5 | . | . | . | . | . | . | . | . | . | . | . | . | . | . | 1 | 3 | . |
| Koeppl et al. (2010) | . | . | . | 11 | . | . | . | 2 | 1 | . | . | . | . | . | . | . | . | . | . | 1 | . | . | . | . | . |
| Koeppl et al. (2012) | 1 | 4 | 4 | 13 | 1 | . | . | 6 | 1 | 2 | . | . | . | . | . | . | . | . | . | . | . | 2 | . | . | . |
| Komorowski et al. (2009) | . | . | . | 4 | 4 | . | . | . | 6 | . | . | . | . | . | . | . | . | . | . | . | . | . | . | 24 | . |
| Komorowski et al. (2011) | . | . | . | 2 | 2 | . | . | . | 13 | . | . | . | . | . | . | . | . | . | . | . | . | . | . | 12 | . |
| Kravaris et al. (2013) | . | . | 1 | . | . | . | . | 2 | . | . | . | . | . | . | . | . | . | . | . | . | . | . | . | . | . |
| Kuepfer et al. (2007) | . | 7 | . | . | . | . | . | . | 5 | 1 | . | . | . | 10 | . | . | . | . | . | . | . | . | . | . | . |
| Kügler (2012) | 2 | 1 | . | 1 | . | 5 | . | 6 | 10 | . | . | 1 | . | . | . | . | . | . | 1 | 3 | 7 | . | 8 | 8 | . |
| Kulikov and Kulikova (2015a) | . | . | . | . | . | . | . | 4 | 35 | . | . | . | . | 1 | . | . | . | . | . | . | 1 | . | . | . | . |
| Kulikov and Kulikova (2015b) | . | . | . | . | . | . | . | 1 | 12 | . | . | . | . | . | . | . | . | . | . | . | . | . | . | . | . |
| Kulikov and Kulikova (2017) | . | . | . | . | . | . | . | 3 | 54 | . | . | . | . | . | . | . | . | . | . | . | . | . | . | . | . |
| Kuntz et al. (2017) | . | 1 | . | 21 | 1 | 1 | . | 14 | . | . | . | 1 | . | 19 | . | . | . | . | . | . | . | . | 2 | . | . |
| Kurt et al. (2016) | . | . | . | 2 | . | . | . | . | . | . | . | . | . | 6 | . | . | . | 1 | . | . | . | . | . | . | . |
| Kutalik et al. (2007) | . | 1 | . | . | . | . | . | . | 5 | . | . | . | 45 | 1 | 1 | . | . | . | . | . | . | . | . | . | . |
| Kuwahara et al. (2013) | . | . | 1 | . | . | . | . | 2 | 10 | 1 | . | . | . | 3 | . | . | . | . | . | . | . | . | . | . | . |
| Kyriakopoulos and Wolf (2015) | . | 1 | . | 7 | . | . | . | 8 | 1 | . | . | . | . | . | . | . | . | . | . | . | . | . | 1 | 2 | . |
| Lakatos et al. (2015) | 5 | . | . | 2 | 2 | . | . | . | 11 | . | . | . | . | . | . | . | . | . | . | . | . | . | 29 | 7 | . |
| Lakatos (2017) | 6 | . | 3 | 2 | 4 | . | . | 12 | 64 | 1 | 1 | . | . | 2 | . | . | . | . | . | . | . | . | 41 | 37 | . |
| Lang and Stelling (2016) | . | . | 1 | . | . | . | . | . | 4 | . | . | . | . | 2 | . | . | . | . | . | . | . | . | . | . | . |
| Lecca et al. (2009) | . | 1 | . | 1 | . | . | . | 1 | 1 | . | . | . | . | 1 | . | . | . | 1 | 1 | . | . | . | . | . | . |

**Table S1.** Coverage of modeling strategies of BRNs. (cont.)

| | Physical laws | | | Random processes | | | | | | Mathematical models | | | | | | Interaction models | | | | CME based models | | | | | |
|---|---|---|---|---|---|---|---|---|---|---|---|---|---|---|---|---|---|---|---|---|---|---|---|---|---|
| Reference | kinetic rate laws | mass action kinetics | mechanistic models | Markov process | Poisson process | birth-death process | telegraph process | state space representation | ODEs, PDEs, SDEs, DDEs | rational model | differential algebraic eqns. | tensor representation | S-system model | polynomial model | manifold map | Petri nets | Boolean networks | neural networks | agent based models | Langevin equation | Fokker-Planck equation | reaction rate equation | moment closure | linear noise approximation | system size expansion |
| Li and Vu (2013) | . | . | 1 | . | . | . | . | . | 6 | . | 1 | . | 2 | . | . | . | . | . | . | . | . | . | . | . | . |
| Li and Vu (2015) | . | 1 | 1 | . | . | . | . | 1 | 2 | . | . | . | . | 7 | 1 | . | . | . | . | . | . | . | . | . | . |
| Liao et al. (2015a) | . | . | . | 3 | . | . | . | 7 | 17 | . | . | 67 | . | . | . | . | . | . | . | 2 | 7 | . | . | . | . |
| Liao (2017) | . | . | 1 | 10 | 18 | 19 | . | 10 | 35 | . | . | 535 | . | 46 | 1 | . | . | . | . | 13 | 42 | 13 | . | 14 | 19 |
| Liepe et al. (2014) | . | . | 3 | 5 | . | . | . | . | 19 | . | . | . | . | . | . | . | . | . | . | . | . | . | . | 2 | . |
| Lillacci and Khammash (2010b) | . | 1 | . | 2 | . | . | . | 2 | 2 | 2 | . | . | . | . | . | . | . | . | 1 | . | . | . | . | . | . |
| Lillacci and Khammash (2012) | . | 1 | . | 2 | . | . | . | 4 | 4 | 1 | . | . | . | . | . | . | . | . | . | . | . | . | . | . | . |
| Linder (2013) | . | 23 | . | 6 | 16 | . | . | . | 56 | . | . | . | . | . | . | . | . | . | 1 | 2 | . | 4 | . | 53 | . |
| Lindera and Rempala (2015) | . | 10 | . | 9 | 2 | . | . | . | 13 | . | . | . | . | . | . | . | . | . | . | 1 | . | 2 | . | 18 | . |
| Liu et al. (2006) | . | . | . | . | . | . | . | . | . | . | . | . | . | . | . | . | . | . | . | . | . | . | . | . | . |
| Liu and Wang (2008a) | . | . | . | . | . | . | . | . | 1 | . | . | . | 42 | 2 | . | . | . | 1 | . | . | . | . | . | . | . |
| Liu and Wang (2008b) | . | 1 | . | . | . | . | . | . | . | . | . | . | 10 | . | . | . | . | 2 | . | . | . | 1 | . | . | . |
| Liu and Wang (2009) | . | 1 | . | . | . | . | . | . | . | . | . | . | 4 | 3 | . | . | . | . | . | . | . | . | . | . | . |
| Liu et al. (2012) | . | . | . | 2 | . | . | . | . | 3 | . | . | . | 52 | . | . | . | 1 | 1 | . | . | . | . | . | . | . |
| Liu and Gunawan (2014) | . | 2 | . | . | . | . | . | . | 61 | . | . | . | . | 4 | . | . | . | . | . | . | . | 4 | . | . | . |
| Liu (2014) | 1 | . | . | 28 | 4 | . | . | 50 | 26 | 7 | . | 3 | . | . | 14 | . | . | 6 | . | 1 | . | . | . | . | . |
| Loos et al. (2016) | . | . | 3 | . | . | . | . | . | 7 | . | . | . | . | . | . | . | . | . | . | . | . | 2 | . | . | . |
| Lötstedt (2018) | . | . | . | 7 | 4 | . | . | . | 12 | . | . | . | . | 1 | . | . | . | . | . | . | 1 | 1 | . | 31 | . |
| Lück and Wolf (2016) | . | 2 | . | 7 | . | . | . | 3 | . | . | . | . | . | 1 | . | . | . | . | . | . | . | . | 14 | 3 | . |
| Mancini et al. (2015) | . | . | . | . | . | . | . | 1 | 11 | 1 | 1 | . | . | . | . | . | . | . | . | . | . | . | . | . | . |
| Mannakee et al. (2016) | . | . | 3 | 7 | . | . | . | . | 4 | . | . | . | . | 1 | 50 | . | . | . | . | 1 | . | 1 | . | . | . |
| Mansouri et al. (2014) | . | 2 | . | 1 | . | . | . | 4 | . | 1 | . | . | 25 | . | . | . | . | . | . | . | . | . | . | . | . |
| Mansouri et al. (2015) | . | 2 | . | 1 | . | . | . | 3 | . | 1 | . | . | 20 | . | . | . | . | . | . | . | . | . | . | . | . |
| Matsubara et al. (2006) | . | 1 | . | . | . | . | . | . | . | 1 | . | . | . | . | . | . | . | 7 | . | . | . | . | . | . | . |
| Mazur (2012) | . | . | . | 140 | 5 | . | . | 35 | 123 | . | 1 | . | 9 | 11 | . | 43 | 42 | 2 | 7 | 2 | . | . | . | . | . |
| Mazur and Kaderali (2013) | . | . | . | 7 | . | . | . | 3 | 4 | . | . | . | . | . | . | . | . | . | . | . | . | . | . | . | . |
| McGoff et al. (2015) | . | . | . | 30 | . | . | . | 22 | 18 | . | . | . | . | . | 9 | . | . | . | . | . | . | . | . | . | . |
| Mendes and Kell (1998) | 1 | 1 | 1 | . | . | . | . | . | 5 | . | . | 3 | . | . | . | . | . | . | . | . | . | . | . | . | . |
| Meskin et al. (2011) | . | . | . | . | . | . | . | 1 | . | . | . | . | 50 | 1 | . | . | . | . | . | . | . | . | . | . | . |
| Meskin et al. (2013) | . | . | . | . | . | . | . | 2 | 1 | . | . | . | 31 | . | . | . | . | . | . | . | . | . | . | . | . |
| Michailidis and d'Alché Buc (2013) | . | . | . | 10 | . | . | . | 1 | 5 | . | . | . | 1 | 1 | . | . | . | . | . | . | . | . | . | . | . |
| Michalik et al. (2009) | . | . | . | . | . | . | . | . | 3 | . | 12 | . | . | . | . | . | . | . | . | . | . | . | . | . | . |
| Mihaylova et al. (2012) | . | . | . | . | . | . | . | 11 | . | . | . | . | . | . | . | . | . | . | . | . | . | . | . | . | . |
| Mihaylova et al. (2014) | . | . | . | 14 | 20 | . | . | 5 | . | . | . | . | . | . | . | . | . | . | 7 | . | . | . | . | . | . |
| Mikeev and Wolf (2012) | . | . | . | 32 | . | . | . | 12 | 22 | . | . | . | . | 2 | . | . | . | . | . | . | . | . | 3 | . | . |
| Mikelson and Khammash (2016) | . | 1 | 2 | 6 | . | 5 | . | . | 2 | . | . | . | . | . | . | . | . | . | . | . | . | . | . | 2 | . |
| Milios et al. (2018) | . | 1 | . | 25 | . | . | . | 18 | 7 | . | . | . | . | 2 | . | . | . | . | 4 | . | . | . | 16 | 5 | . |
| Milner et al. (2013) | 3 | 1 | . | 1 | 3 | . | . | . | 20 | . | . | . | . | . | . | . | . | . | . | 1 | . | . | 30 | 1 | . |
| Mizera et al. (2014) | . | . | . | 32 | . | . | . | 2 | . | . | . | . | . | . | . | . | 1 | . | . | . | . | . | 2 | . | . |
| Moles et al. (2003) | . | . | . | . | . | . | . | . | 3 | . | 2 | . | . | 1 | . | . | . | . | . | . | . | . | . | . | . |
| Jaime and Denis (2015) | . | . | . | . | . | . | . | 2 | 1 | . | 1 | . | . | . | . | . | . | . | . | . | . | . | . | . | . |
| Moritz (2014) | 1 | 1 | 4 | . | . | . | . | 10 | 29 | 2 | 4 | . | . | 11 | . | . | 1 | . | . | . | . | 2 | . | . | . |
| Mozgunov et al. (2018) | . | . | . | 13 | 25 | . | . | 5 | 5 | . | . | . | . | . | . | 2 | . | . | . | 2 | . | . | . | . | . |
| Mu (2010) | 4 | 2 | 1 | . | . | . | . | 2 | 20 | 1 | . | . | 59 | . | . | . | . | 1 | . | . | . | . | . | . | . |
| Müller et al. (2012) | . | . | 1 | 15 | 2 | . | . | . | . | . | . | . | . | . | . | . | . | . | . | 1 | 1 | 1 | . | . | . |
| Murakami (2014) | . | . | . | 7 | . | . | . | . | . | 1 | . | . | . | . | . | . | . | . | 1 | . | . | . | . | . | . |
| Nemeth et al. (2014) | . | . | . | 6 | . | . | . | 4 | . | . | . | . | . | . | . | . | . | . | . | . | . | . | . | . | . |
| Nienaltowski et al. (2015) | . | . | 1 | . | . | . | . | 1 | 5 | . | . | . | . | . | . | . | . | . | . | . | . | . | . | . | . |
| Nim et al. (2013) | . | . | . | . | . | . | . | . | 19 | 1 | . | . | . | 3 | . | . | . | . | . | . | . | . | . | . | . |

**Table S1.** Coverage of modeling strategies of BRNs. (cont.)

| | Physical laws | | | Random processes | | | | | Mathematical models | | | | | | | Interaction models | | | | CME based models | | | | | |
|---|---|---|---|---|---|---|---|---|---|---|---|---|---|---|---|---|---|---|---|---|---|---|---|---|---|
| Reference | kinetic rate laws | mass action kinetics | mechanistic models | Markov process | Poisson process | birth-death process | telegraph process | state space representation | ODEs, PDEs, SDEs, DDEs | rational model | differential algebraic eqns. | tensor representation | S-system model | polynomial model | manifold map | Petri nets | Boolean networks | neural networks | agent based models | Langevin equation | Fokker-Planck equation | reaction rate equation | moment closure | linear noise approximation | system size expansion |
| Nobile et al. (2012) | . | . | . | . | . | . | . | . | . | 1 | . | . | . | . | . | . | . | . | 2 | . | . | . | . | . | . |
| Nobile et al. (2013) | 1 | 1 | 5 | . | . | . | . | . | 6 | . | . | . | 1 | . | . | . | . | . | 3 | . | . | . | . | . | . |
| Nobile et al. (2015) | 1 | . | . | . | . | . | . | . | . | . | . | . | . | . | . | . | . | . | 3 | . | . | . | . | . | . |
| Nobile et al. (2016) | 1 | . | 2 | . | . | . | . | . | 6 | . | . | . | . | . | . | . | . | . | 1 | . | . | . | . | . | . |
| Nobile et al. (2018a) | 1 | . | . | . | . | . | . | . | . | . | . | . | . | . | . | . | . | . | 2 | . | . | . | . | . | . |
| Nobile et al. (2018b) | 1 | . | 1 | . | . | . | . | . | 1 | . | . | . | . | . | . | . | . | . | . | . | . | . | . | . | . |
| Pahle et al. (2012) | . | . | 1 | 1 | . | . | . | . | 4 | 1 | . | . | . | . | . | . | . | . | . | . | 2 | . | . | 53 | . |
| Palmisano (2010) | 2 | 19 | 1 | 20 | . | . | . | 5 | 111 | 9 | . | . | 3 | 1 | . | 3 | 2 | . | 10 | 1 | . | . | . | . | . |
| Pan and Yang (2010) | . | . | . | 6 | . | . | . | . | . | . | . | . | . | . | 4 | . | . | . | . | . | . | . | . | . | . |
| Pantazis et al. (2013) | . | 2 | . | 15 | 2 | 3 | . | . | 8 | . | . | . | . | 1 | . | . | . | . | . | 6 | 1 | . | . | 15 | . |
| Paul (2014) | . | 1 | . | 12 | 16 | 26 | . | 2 | 12 | . | . | . | . | . | . | . | . | . | . | 1 | . | . | 2 | . | . |
| Penas et al. (2017) | . | . | . | . | . | . | . | . | 10 | 1 | 4 | . | . | . | . | . | . | . | . | . | . | . | . | . | . |
| Plesa et al. (2017) | . | . | . | . | . | . | . | 5 | 35 | . | . | 1 | . | 9 | 2 | . | . | . | . | . | . | . | . | . | . |
| Poovathingal and Gunawan (2010) | . | . | . | 1 | . | . | . | . | 1 | . | . | . | . | . | . | . | . | . | . | 1 | . | . | . | . | . |
| Pullen and Morris (2014) | . | . | 7 | 6 | . | . | . | 1 | 6 | . | . | . | . | . | . | . | . | 1 | 1 | . | . | . | . | . | . |
| Quach et al. (2007) | . | 3 | . | 3 | . | . | . | 16 | 23 | . | . | . | . | . | . | . | . | . | . | . | . | . | . | . | . |
| Radulescu et al. (2012) | . | 2 | 6 | 1 | . | . | . | . | 6 | . | . | . | 3 | 17 | 31 | . | . | . | 1 | 1 | 1 | . | . | . | . |
| Rakhshania et al. (2016) | . | . | 1 | 2 | . | . | . | . | 3 | . | . | . | . | . | . | . | . | 2 | . | . | . | . | . | . | . |
| J. O. Ramsay and Cao (2007) | . | . | 3 | 13 | 2 | . | . | 13 | 81 | . | 9 | . | . | 2 | 2 | . | . | 1 | 1 | . | . | . | . | . | . |
| Rapaport and Dochain (2005) | . | . | . | . | . | . | . | 1 | . | . | . | . | . | . | . | . | . | . | . | . | . | . | . | . | . |
| Reinker et al. (2006) | . | 2 | . | 10 | . | . | . | 6 | . | . | . | . | . | . | . | . | . | . | . | . | . | . | . | . | . |
| Reis et al. (2018) | . | . | . | 2 | 65 | . | . | . | 40 | . | . | . | . | 11 | . | . | . | . | . | . | . | 1 | 1 | . | . |
| Remlia et al. (2017) | . | . | 1 | . | . | . | . | . | 6 | . | . | . | . | . | . | . | . | . | 1 | . | . | . | . | . | . |
| Rempala (2012) | . | 5 | . | 10 | 4 | . | . | . | 15 | . | . | . | . | . | . | . | . | . | . | 1 | . | . | . | 1 | . |
| Revell and Zuliani (2018) | . | 2 | . | 4 | 1 | . | . | 2 | 3 | . | . | 1 | . | . | . | . | . | . | . | . | 1 | . | . | 5 | . |
| Rosati et al. (2018) | . | . | . | . | . | . | . | . | 29 | . | . | . | . | 1 | . | . | . | . | . | . | . | . | . | . | . |
| Ruess et al. (2011) | . | . | . | 1 | . | . | . | 3 | . | . | . | . | . | . | . | . | . | . | . | . | . | . | 33 | . | . |
| Ruess (2014) | 1 | . | 4 | 18 | 10 | 1 | . | 8 | 13 | . | . | . | . | 3 | . | . | . | . | 4 | 5 | 5 | 6 | 34 | 20 | . |
| Ruess and Lygeros (2015) | . | 1 | 1 | 8 | 2 | . | . | 3 | 1 | . | . | . | . | 1 | . | . | . | . | . | . | . | . | 21 | . | . |
| Rumschinski et al. (2010) | 1 | 3 | . | . | . | . | . | 1 | . | . | . | . | 1 | 8 | . | . | . | . | 1 | . | . | . | . | . | . |
| Ruttor and Opper (2009) | 2 | . | . | 3 | . | . | . | . | . | . | . | . | . | . | . | . | . | . | . | . | 2 | . | . | . | . |
| Sadamoto et al. (2017) | . | . | . | . | . | . | . | . | . | . | . | . | . | . | . | . | . | . | . | . | . | . | . | . | . |
| Sagar et al. (2017) | 1 | 1 | . | . | . | . | . | . | . | . | . | . | . | . | . | . | . | . | . | . | . | . | . | . | . |
| Schenkendorf (2014) | . | . | 2 | . | . | . | . | 1 | 102 | . | . | 5 | . | 25 | . | . | . | 6 | . | . | . | . | . | . | . |
| Schilling et al. (2016) | 1 | 2 | . | 7 | . | . | . | 4 | 10 | . | . | . | . | 2 | . | . | . | . | . | . | . | . | 44 | 1 | . |
| Schnoerr (2016) | . | . | 1 | 19 | 143 | 5 | . | 16 | 73 | . | . | . | 5 | 19 | . | . | . | . | 1 | 80 | 23 | . | 108 | 28 | 14 |
| Schnoerr et al. (2017) | . | 7 | . | 23 | 20 | 4 | . | 15 | 5 | . | 1 | 2 | . | 3 | . | 2 | . | 1 | 1 | 23 | 7 | . | 76 | 65 | 68 |
| Septier and Peters (2016) | . | . | . | 50 | 1 | . | . | 10 | 7 | . | . | 1 | . | . | 15 | . | . | . | . | 25 | . | . | . | . | . |
| Shacham and Brauner (2014) | . | . | 4 | . | . | . | . | . | 3 | . | 1 | . | . | 11 | . | . | . | 2 | . | . | . | . | . | . | . |
| Sherlock et al. (2014) | . | . | . | 13 | 8 | . | . | 2 | 37 | . | . | . | . | . | . | . | . | . | . | 2 | . | . | . | 44 | . |
| Shiang (2009) | . | . | . | . | . | . | . | . | 39 | 3 | . | . | . | . | . | . | . | . | . | . | . | . | . | . | . |
| Zamora-Sillero et al. (2011) | . | 1 | . | 2 | . | . | . | 2 | . | . | . | . | . | . | . | . | . | . | 2 | . | . | . | . | . | . |
| Singh and Hahn (2005) | . | . | . | . | . | . | . | 1 | 3 | . | . | . | . | . | . | . | . | . | . | . | . | . | . | . | . |
| Slezak et al. (2010) | 1 | . | 3 | . | . | . | . | . | . | . | . | . | . | . | . | . | . | . | 1 | . | . | . | . | . | . |
| Smadbeck (2014) | 32 | 1 | 1 | 6 | 13 | . | . | 10 | 22 | . | . | . | . | 39 | . | . | . | . | . | 1 | . | 1 | 50 | . | . |
| Smet and Marchal (2010) | . | . | 1 | . | . | . | . | . | . | . | . | . | . | . | . | . | . | . | . | . | . | . | . | . | . |
| Smith and Grima (2018) | 1 | 14 | . | 2 | 1 | . | . | . | 4 | 1 | . | 5 | . | . | . | . | . | . | . | 10 | . | . | 1 | . | . |
| He et al. (2004) | . | . | . | . | . | . | . | . | 3 | . | . | . | . | . | . | . | . | . | . | . | . | . | . | . | . |
| Srinath and Gunawan (2010) | . | 1 | . | . | . | . | . | . | 11 | . | . | . | 8 | . | . | . | . | . | . | . | . | . | . | . | . |

**Table S1.** Coverage of modeling strategies of BRNs. (cont.)

| | Physical laws | | | Random processes | | | | | Mathematical models | | | | | | | Interaction models | | | | | CME based models | | | | |
|---|---|---|---|---|---|---|---|---|---|---|---|---|---|---|---|---|---|---|---|---|---|---|---|---|---|
| Reference | kinetic rate laws | mass action kinetics | mechanistic models | Markov process | Poisson process | birth-death process | telegraph process | state space representation | ODEs, PDEs, SDEs, DDEs | rational model | differential algebraic eqns. | tensor representation | S-system model | polynomial model | manifold map | Petri nets | Boolean networks | neural networks | agent based models | Langevin equation | Fokker-Planck equation | reaction rate equation | moment closure | linear noise approximation | system size expansion |
| Srinivas and Rangaiah (2007) | . | . | . | . | . | . | . | . | . | . | . | . | . | . | . | . | . | . | . | . | . | . | . | . | . |
| Srivastava (2012) | . | 1 | . | 13 | 2 | . | . | 5 | 3 | . | . | . | . | . | . | . | . | . | . | 6 | 3 | . | . | 1 | . |
| Srivastavaa and Rawlingsb (2014) | . | 1 | . | 5 | . | . | . | 1 | 1 | . | . | . | . | . | . | . | . | . | . | . | 1 | . | . | . | . |
| von Stosch et al. (2014) | . | . | 31 | . | . | . | . | . | 5 | . | . | . | . | . | . | . | . | 51 | . | . | . | . | . | . | . |
| Emmert-Streib et al. (2012) | . | . | . | 3 | . | . | . | . | . | . | . | . | . | . | . | . | 2 | . | . | . | . | . | . | . | . |
| Sun et al. (2008) | . | . | 2 | . | . | . | . | 22 | 1 | 1 | . | . | . | 1 | . | . | . | . | . | . | . | . | . | . | . |
| Sun et al. (2012) | . | 1 | . | 2 | . | . | . | . | 11 | 4 | 5 | . | 34 | 1 | . | . | . | 2 | 1 | 1 | . | . | . | . | . |
| Sun et al. (2014) | 2 | . | . | 2 | . | . | . | . | . | . | . | . | 4 | . | . | . | . | 1 | . | . | . | . | . | . | . |
| Swaminathan and Murray (2014) | . | . | . | 21 | . | . | . | 1 | . | . | . | . | . | . | . | . | . | . | . | . | . | . | . | . | . |
| Tanevski et al. (2010) | . | 2 | . | 6 | 5 | . | . | 1 | 1 | . | . | . | . | . | . | . | . | . | . | . | . | 1 | . | . | . |
| Tangherloni et al. (2016) | 1 | . | 1 | 1 | . | . | . | . | 6 | . | . | . | . | . | . | . | . | . | . | . | . | . | . | . | . |
| Teijeiro et al. (2017) | . | . | . | . | . | . | . | . | 48 | 2 | . | . | . | . | . | . | . | . | . | . | . | . | . | . | . |
| Tenazinha and Vinga (2011) | 1 | 1 | 3 | . | . | . | . | 1 | 39 | . | . | . | . | . | . | . | . | . | . | 1 | . | . | . | . | . |
| Thomas et al. (2012) | 5 | . | . | . | 4 | . | . | . | . | . | . | . | . | . | . | . | . | . | . | 35 | 4 | . | . | 175 | . |
| Tian et al. (2007) | . | . | . | 1 | 7 | . | . | . | 29 | . | . | . | . | . | . | . | . | . | . | 1 | . | . | . | . | . |
| Tian et al. (2010) | . | . | . | . | . | . | . | . | . | . | . | . | . | . | . | . | . | . | . | . | . | . | . | . | . |
| Toni and Stumpf (2010) | . | 2 | 3 | 2 | . | . | . | 1 | 9 | 1 | . | . | . | . | . | 1 | . | . | . | . | . | . | . | . | . |
| Transtrum and Qiu (2012) | . | . | . | 1 | . | . | . | . | . | . | . | . | . | . | 3 | . | . | . | 2 | . | . | . | . | . | . |
| Siegal-Gaskins et al. (2015) | . | 5 | . | 1 | . | . | . | . | 12 | . | . | . | . | . | . | . | . | . | . | . | . | . | . | . | . |
| Vanlier et al. (2013) | . | . | 1 | 8 | . | . | . | 1 | 3 | 7 | . | . | . | . | 2 | . | . | . | . | 1 | . | . | . | . | . |
| Vargas et al. (2014) | . | . | . | . | . | . | . | . | . | . | . | . | . | . | . | . | . | . | . | . | . | . | . | . | . |
| Veerman et al. (2018) | . | . | . | . | 3 | . | 12 | . | . | . | . | . | . | . | . | . | . | . | . | . | . | . | . | . | . |
| Venayak et al. (2018) | . | . | . | . | . | . | . | . | . | . | . | . | . | . | . | . | . | . | . | . | . | . | . | . | . |
| Villaverde et al. (2012) | 8 | 2 | 1 | . | . | . | . | . | 5 | . | 1 | . | . | . | . | . | . | . | . | . | . | . | . | . | . |
| Villaverde et al. (2014) | . | . | . | 1 | . | . | . | . | . | . | . | . | . | . | . | . | . | 1 | 1 | . | . | . | . | . | . |
| Villaverde et al. (2016) | . | . | . | . | . | . | . | . | 3 | 4 | . | . | . | 3 | . | . | . | . | . | . | . | . | . | . | . |
| Villaverde and Barreiro (2016) | . | . | 1 | . | . | . | . | 1 | 13 | . | . | . | . | 1 | . | . | . | . | . | . | . | . | . | . | . |
| Voit (2013) | 6 | 5 | 3 | . | 1 | . | . | . | 13 | 3 | . | 1 | 200 | 9 | 1 | 4 | . | 4 | . | . | . | . | . | . | . |
| Vrettas et al. (2011) | . | . | . | 14 | . | . | . | 1 | 19 | . | . | . | . | 53 | . | . | . | . | . | 3 | . | . | . | . | . |
| Wang et al. (2010) | 3 | 1 | . | 12 | . | 5 | . | . | . | . | . | . | . | . | . | . | . | . | . | 2 | . | . | . | . | . |
| Weber and Frey (2017) | . | 1 | . | 44 | 109 | 8 | 5 | 48 | 84 | . | . | 1 | . | 20 | 2 | . | . | 4 | . | 8 | 58 | . | 4 | . | . |
| Weiss et al. (2016) | . | . | . | 1 | . | . | . | . | . | . | . | . | . | . | 14 | . | . | 5 | . | . | . | . | . | . | . |
| Whitaker et al. (2017) | . | . | 1 | 13 | 1 | 1 | . | . | 50 | . | . | . | . | . | . | . | . | . | . | . | 2 | . | 1 | 75 | . |
| White et al. (2015) | . | . | . | 12 | . | . | . | 1 | 3 | . | . | . | . | . | . | . | . | . | . | . | . | . | . | . | . |
| White et al. (2016) | . | 2 | 37 | . | . | . | . | . | 2 | . | . | . | . | . | 22 | . | . | . | 1 | . | . | . | . | . | . |
| Wong et al. (2015) | . | 31 | 7 | . | . | . | . | . | 5 | . | . | 16 | . | . | . | . | . | . | . | . | . | . | . | . | . |
| Woodcock et al. (2011) | 1 | . | . | 5 | . | . | . | . | 12 | 1 | . | . | . | . | . | . | . | . | . | . | . | . | . | 17 | . |
| Xiong and Zhou (2013) | . | . | . | 1 | . | . | . | 12 | . | . | . | . | . | . | . | . | . | . | . | . | . | . | . | . | . |
| Yang et al. (2014) | . | . | 5 | 2 | . | . | . | 2 | 2 | . | . | . | . | . | . | . | . | . | . | . | . | . | . | . | . |
| Yang et al. (2012) | . | . | . | . | . | . | . | . | . | . | . | . | 16 | . | . | . | 1 | . | . | . | . | . | . | . | . |
| Yenkie et al. (2016) | . | 2 | . | 2 | 4 | . | . | . | 67 | . | . | . | . | . | . | . | . | . | . | . | . | . | . | . | . |
| Zechner et al. (2011) | . | . | . | 6 | 1 | . | . | 5 | . | . | . | . | . | . | . | . | . | . | . | . | . | . | . | . | . |
| Zechner et al. (2012) | . | . | . | 5 | 1 | 3 | . | 1 | 4 | . | . | . | . | . | . | . | . | . | . | . | . | . | 1 | . | . |
| Zechner (2014) | . | . | . | 56 | 20 | 10 | . | 12 | 38 | . | . | . | . | 3 | . | . | . | . | . | . | 2 | . | 5 | 4 | . |
| Zeng et al. (2012) | . | . | . | 4 | . | . | . | 5 | 3 | . | . | . | . | . | . | . | . | . | . | . | . | . | . | . | . |
| Zhan and Yeung (2011) | . | . | . | . | . | . | . | . | 16 | . | . | . | 6 | . | 1 | . | . | 1 | . | . | . | . | . | . | . |
| Zhan et al. (2014) | . | . | . | . | . | . | . | . | 52 | . | . | . | 7 | . | . | 3 | . | . | . | . | . | . | . | . | . |
| Zimmer et al. (2014) | . | . | . | 3 | . | . | . | . | 14 | . | 1 | . | . | . | 4 | . | . | . | . | 1 | . | . | . | 4 | . |
| Zimmer and Sahle (2012) | 1 | . | . | 4 | . | . | . | 5 | 51 | 1 | 1 | . | . | . | 1 | . | . | . | . | . | . | . | . | 2 | . |

**Table S1.** Coverage of modeling strategies of BRNs. (cont.)

| | Physical laws | | | Random processes | | | | Mathematical models | | | | | | | | Interaction models | | | | CME based models | | | | | |
|---|---|---|---|---|---|---|---|---|---|---|---|---|---|---|---|---|---|---|---|---|---|---|---|---|---|
| Reference | kinetic rate laws | mass action kinetics | mechanistic models | Markov process | Poisson process | birth-death process | telegraph process | state space representation | ODEs, PDEs, SDEs, DDEs | rational model | differential algebraic eqns. | tensor representation | S-system model | polynomial model | manifold map | Petri nets | Boolean networks | neural networks | agent based models | Langevin equation | Fokker-Planck equation | reaction rate equation | moment closure | linear noise approximation | system size expansion |
| Zimmer and Sahle (2015) | 4 | 1 | . | 5 | . | . | . | 2 | 17 | 1 | . | . | . | . | . | . | . | . | . | . | . | . | . | 23 | . |
| Zimmer (2015) | 3 | . | . | 13 | . | 1 | . | 5 | 12 | 3 | . | . | . | . | . | . | . | . | . | . | . | . | 1 | 13 | . |
| Zimmer (2016) | 4 | . | . | 1 | . | . | . | 5 | 15 | 3 | 2 | . | . | . | . | . | . | . | 1 | . | . | . | 4 | 47 | . |
| **# papers** | 63 | 102 | 84 | 170 | 74 | 21 | 2 | 152 | 219 | 60 | 27 | 21 | 39 | 90 | 35 | 13 | 13 | 37 | 47 | 55 | 35 | 19 | 46 | 51 | 4 |

**Table S2.** Coverage of parameter estimation strategies for BRNs.

| | Tasks | | | | Measures | | | | | Bayesian methods | | | | Monte Carlo | | | Kalman filter | | | Model fitting | | | | | | | XLR |
|---|---|---|---|---|---|---|---|---|---|---|---|---|---|---|---|---|---|---|---|---|---|---|---|---|---|---|---|
| Reference | identifi., observab., reachability | optimum experiment design | bifurcation analysis | inference, identification | sensitivity analysis | confidence/credible intervals | Akaike/Fisher/mutual info. | entropy | sum of squared errors | MAP, ML, likelihood | approximate Bayesian comput. | expectation-maximization | variational Bayesian inference | MCMC | Metropol./import. sampling | sequential MC, particle filters | Kalman filter | extended Kalman filter | unscented Kalman filter | LS and regression | genetic algorithms | optimization programming | simulated annealing | differential evolution | scatter, tabu, cuckoo search | particle swarm optimization | other algorithms | mach./deep/transf. learning |
| Abdullah et al. (2013c) | . | . | . | 37 | . | . | . | . | . | 2 | . | . | . | . | . | . | 1 | 4 | . | . | 5 | . | . | 35 | 2 | 59 | . | . |
| Abdullah et al. (2013b) | 11 | . | . | 60 | . | . | 2 | . | . | 2 | . | . | . | . | . | . | 4 | 4 | . | . | 4 | . | 1 | 27 | 2 | 6 | 9 | 1 |
| Abdullah et al. (2013a) | 15 | . | . | 57 | . | . | 1 | . | . | 4 | . | . | . | . | . | . | . | . | . | 1 | 3 | . | 2 | 12 | 2 | 27 | 12 | 1 |
| Alberton et al. (2013) | 60 | . | 1 | 66 | 5 | . | 2 | . | . | 1 | . | . | . | . | . | . | . | . | . | . | 2 | 2 | . | . | . | 2 | 1 | 1 |
| Ale et al. (2013) | 1 | 1 | . | 20 | 6 | . | . | 2 | . | 4 | . | . | . | . | . | . | . | . | . | . | . | . | . | . | 2 | . | . | . |
| Ali et al. (2015) | 45 | . | . | 119 | . | . | . | . | . | 1 | . | 1 | . | . | . | 1 | 25 | 23 | 7 | . | 4 | 2 | 1 | . | 1 | . | 25 | . |
| Amrein and Künsch (2012) | . | 1 | . | 18 | . | . | . | . | . | 16 | . | . | . | 9 | 4 | 1 | . | . | . | . | . | . | . | . | . | . | . | . |
| Anai et al. (2006) | . | . | . | 33 | . | 3 | . | . | . | . | . | . | . | . | . | . | . | . | . | 1 | . | 2 | . | 1 | . | . | . | . |
| Andreychenko et al. (2011) | . | . | . | 43 | . | . | . | . | . | 76 | . | . | . | . | . | . | . | . | . | . | . | . | . | . | . | . | . | . |
| Andreychenko et al. (2012) | 5 | . | . | 50 | . | . | 5 | . | . | 48 | . | . | . | . | . | . | . | . | . | . | . | 1 | . | . | 2 | . | . | . |
| Andreychenko et al. (2015) | . | . | . | 7 | . | . | . | 54 | . | 3 | . | . | . | . | . | . | . | . | . | . | . | 1 | . | . | 1 | . | . | . |
| Andrieu et al. (2010) | 2 | . | . | 85 | . | . | . | 1 | . | 127 | 12 | . | . | 210 | 80 | 278 | 6 | . | . | . | . | 3 | 1 | . | . | . | . | . |
| Angius and Horváth (2011) | . | . | . | 27 | . | . | . | . | . | 22 | . | 12 | . | . | . | . | . | . | . | . | . | 1 | . | . | . | . | . | . |
| Arnold et al. (2014) | 1 | . | . | 35 | 3 | . | . | . | . | 16 | . | . | . | 3 | . | 4 | 26 | . | . | . | . | . | 1 | . | . | . | . | . |
| Ashyraliyev et al. (2009) | 24 | 1 | . | 56 | . | 8 | . | . | . | 22 | . | . | 2 | . | 5 | . | . | . | . | 5 | 6 | 6 | 17 | 2 | 3 | 1 | . | . |
| Atitey et al. (2018b) | . | . | . | 5 | . | . | . | . | . | 12 | . | . | . | 1 | 1 | . | . | . | . | . | . | 1 | . | . | 8 | . | . | . |
| Atitey et al. (2018a) | . | . | . | 2 | . | . | . | . | . | 1 | . | . | . | . | . | . | . | . | . | . | . | 3 | 1 | . | . | . | . | . |
| Atitey et al. (2019) | . | . | . | 2 | . | . | . | . | . | . | . | . | . | . | . | . | . | . | . | . | . | . | . | . | 20 | . | . | . |
| Azab et al. (2018) | . | . | . | 28 | . | . | . | 3 | . | 1 | . | . | . | . | 3 | 3 | . | . | . | 1 | . | . | . | . | . | . | 3 | 77 |
| Babtie and Stumpf (2017) | 11 | . | . | 54 | 4 | 3 | 1 | . | . | 36 | 5 | 2 | . | . | . | . | . | . | . | . | . | . | . | . | 1 | . | . | . |
| Backenköhler et al. (2016) | . | . | . | 52 | . | . | . | . | . | 19 | 2 | . | . | . | . | . | . | . | . | . | . | . | . | . | 1 | . | . | . |
| Backenköhler et al. (2018) | 5 | . | . | 81 | . | . | 1 | 1 | . | 20 | 2 | . | . | . | . | . | . | . | . | 3 | 2 | 2 | . | . | 1 | . | . | 1 |
| Baker et al. (133, 2010) | . | . | . | 17 | . | . | . | . | . | . | . | . | . | . | . | . | . | . | . | . | 8 | 10 | 16 | . | . | 15 | . | . |
| Baker et al. (2011) | 54 | . | . | 46 | 4 | . | . | . | . | 5 | . | . | . | . | . | . | 22 | 10 | 47 | 2 | 2 | . | 3 | . | . | . | . | . |
| Baker et al. (2013) | 1 | . | . | 79 | . | . | . | . | . | 7 | . | . | . | 2 | . | 9 | 30 | 13 | 69 | . | 1 | 1 | 4 | . | . | 1 | . | . |
| Baker et al. (2015) | 130 | . | . | 151 | 1 | 21 | 1 | . | . | 77 | . | . | . | 1 | . | 7 | 26 | 4 | 69 | 4 | . | 1 | . | . | 1 | . | . | . |
| Banga and Canto (2008) | 17 | . | . | 50 | 1 | 8 | 3 | . | . | 4 | . | . | . | . | . | . | . | . | . | 2 | . | 2 | . | 2 | 2 | . | . | 1 |
| Barnes et al. (2011) | 1 | 9 | . | 41 | 3 | . | 1 | . | . | 45 | 27 | . | . | 3 | . | 11 | 16 | 1 | 25 | . | . | 1 | . | 1 | 1 | . | 25 | . |
| Bayer et al. (2016) | . | . | . | 36 | . | . | . | . | . | 38 | . | 34 | . | 5 | . | . | . | . | . | . | . | . | . | . | . | . | . | . |
| Berrones et al. (2016) | . | . | . | 29 | . | . | . | 1 | . | 19 | . | . | . | . | . | . | 1 | 1 | . | . | 10 | 5 | . | 3 | . | 2 | 9 | . |
| Besozzi et al. (2009) | . | . | . | 10 | . | . | . | . | . | . | . | . | . | . | . | . | . | . | . | . | 15 | . | . | . | . | 104 | 1 | . |
| Bhaskar et al. (2010) | . | . | . | 11 | . | . | . | . | . | 1 | . | . | . | . | . | . | . | . | . | . | . | . | . | 2 | . | . | . | . |
| Blei et al. (2017) | . | . | . | 209 | . | . | 2 | 1 | . | 67 | . | 14 | 319 | 32 | 4 | . | 1 | . | . | 14 | . | 2 | . | . | 3 | . | 6 | 4 |
| Bogomolov et al. (2015) | . | . | . | 34 | . | . | . | . | . | 25 | . | . | . | . | 1 | . | 1 | 1 | . | . | . | 2 | . | . | . | . | . | . |
| Bouraoui et al. (2015) | . | . | . | 42 | . | . | . | . | . | . | . | . | . | . | . | . | . | . | . | . | . | . | . | . | . | . | . | . |
| Farza et al. (2016) | 2 | . | . | 28 | . | . | . | . | . | . | . | . | . | . | . | . | . | . | . | . | . | . | 5 | . | . | . | . | . |
| Boys et al. (2008) | . | 1 | . | 27 | . | . | . | . | . | 25 | . | . | . | 15 | 5 | . | . | . | . | . | 1 | 1 | . | . | 1 | . | . | . |
| Brim et al. (2013) | 2 | 3 | . | 10 | 1 | . | . | . | . | 4 | . | . | . | . | . | . | . | . | . | . | . | 2 | . | . | . | . | . | . |
| Bronstein et al. (2015) | 5 | . | . | 76 | . | . | . | 4 | . | 55 | 5 | 1 | 1 | 10 | 2 | 11 | 1 | . | . | . | . | 2 | . | . | 10 | . | . | 1 |
| Bronstein and Koeppl (2018) | . | . | . | 2 | . | . | 7 | 30 | . | 1 | . | . | 44 | . | . | . | . | . | . | . | . | 1 | . | . | 2 | . | . | . |
| Busetto and Buhmann (2009) | 4 | . | . | 54 | . | . | . | . | . | 26 | . | . | . | 5 | 3 | 11 | . | . | . | . | . | 1 | . | . | . | . | . | . |
| Camacho et al. (2018) | . | . | . | 22 | . | . | . | 1 | . | . | . | . | . | . | . | . | . | . | . | 10 | . | 4 | . | . | 1 | . | 24 | 126 |
| Balsa-Canto et al. (2008) | . | 1 | . | 28 | . | . | . | . | . | 2 | . | . | . | . | . | . | . | . | . | . | 2 | 4 | 1 | 8 | 2 | . | . | . |
| Carmi et al. (2013) | 2 | . | . | 47 | . | 1 | 1 | 3 | . | 24 | . | . | . | 27 | 10 | 12 | 6 | . | . | . | . | 1 | . | . | . | 1 | . | . |
| Cazzaniga et al. (2015) | . | . | . | 11 | . | . | . | . | . | . | . | . | . | . | . | . | . | . | . | . | 2 | . | . | 3 | . | 54 | 1 | . |
| Cedersund et al. (2016) | 2 | . | . | 39 | . | . | . | . | . | 7 | . | . | . | . | . | . | . | . | . | . | 5 | 5 | 7 | . | 9 | 12 | 4 | 2 |
| Česka et al. (2014) | 3 | 3 | . | 10 | 1 | . | . | . | . | 4 | . | 1 | . | . | . | . | . | . | . | . | . | 2 | . | . | . | . | . | . |
| Češka et al. (2017) | 8 | 2 | . | 8 | 1 | . | . | . | . | . | . | . | . | . | . | . | . | . | . | 1 | . | 1 | . | . | . | . | . | . |
| Chen et al. (2017) | . | . | . | 13 | . | . | . | . | . | 5 | . | . | . | . | . | . | . | . | . | . | . | . | . | . | . | . | . | . |

**Table S2.** Coverage of parameter estimation strategies for BRNs. (cont.)

| | Tasks | | | | Measures | | | | | Bayesian methods | | | | Monte Carlo | | | Kalman filter | | | Model fitting | | | | | | | | XLR |
|---|---|---|---|---|---|---|---|---|---|---|---|---|---|---|---|---|---|---|---|---|---|---|---|---|---|---|---|---|
| Reference | identifi., observab., reachability | optimum experiment design | bifurcation analysis | inference, identification | sensitivity analysis | confidence/credible intervals | Akaike/Fisher/mutual info. | entropy | sum of squared errors | MAP, ML, likelihood | approximate Bayesian comput. | expectation-maximization | variational Bayesian inference | MCMC | Metropol./import. sampling | sequential MC, particle filters | Kalman filter | extended Kalman filter | unscented Kalman filter | LS and regression | genetic algorithms | optimization programming | simulated annealing | differential evolution | scatter, tabu, cuckoo search | particle swarm optimization | other algorithms | mach./deep/transf. learning |
| Chevaliera and Samadb (2011) | . | . | . | 6 | . | . | . | . | . | . | . | . | . | . | . | . | . | . | . | . | . | . | . | . | . | . | . | . |
| Chong et al. (2012) | . | 1 | . | 38 | . | . | . | . | . | . | . | . | . | . | . | . | 10 | . | 1 | . | 5 | 3 | 9 | 38 | . | 1 | 1 | . |
| Chong et al. (2014) | . | . | . | 65 | . | . | . | . | . | . | . | . | . | . | . | 1 | 6 | . | 1 | . | 6 | . | 15 | 61 | 3 | 7 | 14 | . |
| Chou et al. (2006) | . | . | . | 43 | . | . | . | . | 3 | 1 | . | . | . | . | . | . | . | . | . | 42 | 2 | . | . | . | . | . | 1 | . |
| Chou and Voit (2009) | 3 | 1 | . | 232 | . | . | . | 3 | 2 | 6 | . | . | . | . | . | . | 1 | . | . | 25 | 15 | 26 | 10 | 8 | . | 10 | 7 | . |
| Cseke et al. (2016) | . | . | . | 43 | . | . | . | . | . | 50 | . | . | 23 | 1 | . | . | 1 | . | . | 1 | . | . | . | . | 2 | . | . | 1 |
| Dai and Lai (2010) | 1 | . | . | 25 | . | . | 1 | . | 1 | 1 | . | . | . | . | 3 | . | . | . | . | . | 1 | 4 | 18 | 10 | 2 | . | . | . |
| Daigle et al. (2012) | . | . | . | 32 | 1 | 6 | . | 15 | . | 89 | 4 | 16 | . | . | 3 | 1 | . | . | . | . | 3 | 1 | . | . | 2 | . | . | . |
| Dargatz (2010) | 1 | 2 | . | 527 | 1 | 17 | 3 | . | . | 251 | . | 3 | 1 | 100 | 22 | 11 | 1 | . | . | 1 | . | 1 | . | . | . | . | . | . |
| Dattner (2015) | 19 | . | . | 76 | . | . | . | . | . | . | . | . | . | . | . | . | . | . | . | 5 | . | 1 | . | . | . | . | . | . |
| Deng and Tian (2014) | 2 | 1 | . | 51 | 1 | . | . | . | . | 22 | 5 | . | . | 1 | . | 3 | . | . | . | . | 13 | 1 | 2 | . | . | . | . | . |
| Dey et al. (2018) | 1 | . | . | 35 | . | . | . | . | . | 5 | . | . | . | . | . | 3 | 39 | 26 | 1 | . | . | . | . | . | . | . | . | . |
| Dinh and Sidje (2017) | 1 | . | . | 22 | . | . | . | . | . | 71 | . | . | . | . | 1 | . | . | . | . | . | . | 2 | 6 | . | 4 | . | . | . |
| Dochain (2003) | . | . | . | 28 | . | . | . | . | . | . | . | . | . | . | . | . | 7 | 2 | . | . | . | 1 | . | . | . | . | . | . |
| Drovandi et al. (2016) | . | . | . | 48 | . | . | . | . | . | 111 | 38 | . | . | 47 | 9 | 3 | . | . | . | . | . | . | . | . | . | . | . | . |
| Eghtesadi and Mcauley (2014) | 6 | . | . | 47 | 3 | 3 | 5 | . | . | 2 | . | 12 | . | . | . | . | . | . | . | 16 | 1 | . | . | . | . | . | . | . |
| Eisenberg and Hayashi (2014) | 77 | . | . | 15 | . | 1 | 6 | . | . | 39 | . | . | . | . | . | . | . | . | . | . | . | . | . | . | . | . | . | . |
| Engl et al. (2009) | . | 109 | . | 49 | . | 2 | 1 | 5 | . | 9 | . | . | 5 | . | . | . | . | . | . | 4 | 3 | 3 | . | 1 | 1 | . | . | . |
| Erguler and Stumpf (2011) | . | 14 | . | 16 | 9 | 3 | 2 | . | . | 36 | . | . | . | . | . | . | . | . | . | . | . | . | . | . | 2 | . | . | . |
| Fages et al. (2015) | 1 | 2 | . | 21 | 2 | . | . | . | . | 29 | . | . | . | . | . | . | . | . | . | . | . | 1 | . | . | 1 | . | . | 1 |
| Famili et al. (2005) | . | . | . | 5 | . | . | . | . | . | . | . | . | . | . | . | . | . | . | . | . | . | 3 | . | . | . | . | . | . |
| Farina et al. (2006) | 38 | . | . | 39 | . | . | . | . | . | 1 | . | . | . | . | . | . | . | . | . | . | . | . | . | 2 | . | . | . | . |
| Fearnhead and Prangle (2012) | . | . | . | 148 | . | 3 | 2 | . | . | 235 | 524 | . | . | 18 | 22 | 26 | . | . | . | 84 | . | 2 | 2 | . | . | . | 449 | 1 |
| Fearnhead et al. (2014) | . | . | . | 52 | . | 11 | . | . | 1 | 35 | . | . | . | 15 | 3 | 22 | 1 | . | . | . | . | . | . | . | . | . | . | . |
| Rodriguez-Fernandez et al. (2006b) | 60 | 1 | . | 58 | 2 | 8 | 13 | . | . | 11 | . | . | . | . | . | . | . | . | . | 1 | 2 | 4 | 4 | . | . | . | . | . |
| Rodriguez-Fernandez et al. (2006a) | 31 | . | . | 58 | 2 | 9 | 4 | . | . | 16 | . | . | 1 | . | . | . | . | . | . | 1 | 4 | 7 | 2 | 8 | 16 | . | . | 1 |
| Rodriguez-Fernandez et al. (2013) | 22 | . | . | 47 | 10 | 2 | 3 | . | . | 11 | . | . | . | 1 | . | . | 1 | . | . | 1 | . | 12 | . | . | 3 | . | . | . |
| Fey et al. (2008) | 23 | . | . | 24 | . | . | . | . | 1 | 1 | . | . | . | . | . | . | 1 | . | . | 2 | . | . | . | . | . | . | 1 | . |
| Fey and Bullinger (2010) | 5 | . | . | 25 | 1 | . | . | . | . | 1 | . | . | . | . | . | . | . | . | . | . | . | 21 | . | . | . | . | . | . |
| Flassig (2014) | 62 | . | . | 185 | 1 | 23 | 10 | 3 | . | 168 | . | . | 1 | 3 | . | 5 | 2 | . | . | 42 | 2 | 6 | . | 18 | 1 | . | . | 5 |
| Folia and Rattray (2018) | 2 | . | . | 45 | . | . | . | . | . | 37 | . | . | . | 34 | 3 | . | 14 | . | . | . | . | . | . | . | 1 | . | . | . |
| Fröhlich et al. (2014) | 1 | . | . | 23 | . | 3 | . | . | . | 49 | . | . | 1 | 15 | . | . | . | . | . | 17 | . | . | . | . | . | . | . | . |
| Fröhlich et al. (2016) | 15 | 1 | . | 145 | 2 | 7 | . | . | 38 | 40 | . | 45 | . | 5 | . | . | . | . | . | 1 | . | 1 | 1 | 1 | . | . | . | . |
| Fröhlich et al. (2017) | 2 | . | . | 53 | 98 | . | 3 | . | . | 20 | . | . | 1 | 1 | . | . | . | . | . | 2 | 2 | 3 | 4 | 1 | 2 | 1 | . | . |
| Gábor and Banga (2014) | 1 | . | . | 79 | . | . | 1 | . | . | 3 | . | . | . | . | . | . | . | . | . | 11 | . | . | . | . | . | . | . | 1 |
| Gábor et al. (2017) | 51 | . | . | 44 | 5 | 1 | 1 | . | . | 1 | . | . | . | . | . | . | . | . | . | 3 | 1 | 2 | 1 | . | 5 | . | . | . |
| Galagali (2016) | . | . | . | 287 | 1 | . | 1 | 3 | . | 414 | . | 32 | . | 155 | 51 | . | . | . | . | 5 | . | 1 | . | 4 | . | . | . | 1 |
| Geffen et al. (2008) | 94 | . | . | 11 | . | . | . | . | . | . | . | . | . | . | . | . | . | . | . | . | . | . | . | 2 | . | . | . | . |
| Gennemark and Wedelin (2007) | . | . | . | 86 | . | . | . | . | . | 9 | . | . | . | . | . | . | . | . | . | . | 3 | 3 | . | . | . | . | . | 1 |
| Ghusinga et al. (2017) | . | . | . | 10 | . | . | . | 1 | . | . | . | . | . | . | . | . | . | . | . | . | . | 7 | . | 2 | 2 | . | 1 | . |
| Gillespie and Golightly (2012) | . | . | . | 29 | . | . | . | . | . | 11 | . | . | . | 3 | . | 3 | . | . | . | . | . | . | . | . | . | . | . | . |
| Golightly and Wilkinson (2006) | . | 1 | . | 30 | . | . | . | . | . | 28 | . | . | . | 20 | 1 | 4 | . | . | . | . | . | . | . | . | . | . | . | . |
| Golightly and Wilkinson (2005) | . | . | . | 31 | . | . | . | . | . | 17 | . | . | . | 19 | 4 | . | . | . | . | . | . | . | . | . | . | . | . | . |
| Golightly and Wilkinson (2011) | . | 1 | . | 43 | . | . | . | . | . | 60 | . | . | . | 21 | 10 | 39 | . | . | . | . | . | 1 | . | . | . | . | . | . |
| Golightly et al. (2012) | 1 | . | . | 30 | . | . | . | . | . | 98 | . | . | . | 18 | 9 | 37 | . | . | . | . | . | . | . | . | . | . | . | . |
| Golightly et al. (2015) | 1 | . | . | 32 | . | . | . | . | . | 129 | . | . | . | 13 | 12 | 34 | 1 | . | 1 | . | . | . | . | . | . | . | . | . |
| Golightly and Kypraios (2017) | . | 1 | . | 25 | . | 2 | . | . | . | 64 | . | . | . | 67 | 16 | 93 | . | . | . | . | . | . | 1 | . | . | . | . | . |
| Golightly et al. (2019) | . | . | . | 39 | . | . | . | . | . | 48 | . | . | . | 8 | 14 | 3 | . | . | . | . | . | . | . | . | . | . | . | . |
| González et al. (2013) | . | . | . | 43 | . | 9 | . | . | . | 42 | . | 9 | . | . | . | . | 1 | 1 | . | 1 | . | . | . | . | 4 | . | 2 | 1 |

**Table S2.** Coverage of parameter estimation strategies for BRNs. (cont.)

| | Tasks | | | | Measures | | | | | Bayesian methods | | | | Monte Carlo | | | Kalman filter | | | Model fitting | | | | | | | | XLR |
|---|---|---|---|---|---|---|---|---|---|---|---|---|---|---|---|---|---|---|---|---|---|---|---|---|---|---|---|---|
| Reference | identifi., observab., reachability | optimum experiment design | bifurcation analysis | inference, identification | sensitivity analysis | confidence/credible intervals | Akaike/Fisher/mutual info. | entropy | sum of squared errors | MAP, ML, likelihood | approximate Bayesian comput. | expectation-maximization | variational Bayesian inference | MCMC | Metropol./import. sampling | sequential MC, particle filters | Kalman filter | extended Kalman filter | unscented Kalman filter | LS and regression | genetic algorithms | optimization programming | simulated annealing | differential evolution | scatter, tabu, cuckoo search | particle swarm optimization | other algorithms | mach./deep/transf. learning |
| Gordon et al. (1993) | . | . | . | 16 | . | . | . | . | . | 39 | . | . | . | . | 1 | . | 7 | 29 | . | . | . | 1 | . | . | . | . | . | . |
| Guillén-Gosálbez et al. (2013) | 4 | . | . | 43 | . | . | . | . | . | 1 | . | . | . | . | . | . | . | . | . | 1 | . | 5 | . | . | . | . | . | . |
| Goutsias and Jenkinson (2013) | . | 2 | . | 33 | 11 | . | . | 45 | . | 2 | . | . | . | . | 2 | . | 1 | 1 | . | . | . | 2 | 2 | . | . | . | 20 | . |
| Gratie et al. (2013) | 9 | . | . | 23 | 16 | . | . | . | . | . | . | . | . | . | . | . | . | . | . | . | . | . | 1 | . | . | . | . | . |
| Gupta (2013) | . | 8 | . | 156 | . | . | . | 1 | 1 | 280 | 6 | . | . | 174 | 64 | . | . | . | . | 10 | . | 6 | . | . | 1 | . | . | 1 |
| Gupta and Rawlings (2014) | . | 2 | . | 56 | . | . | . | . | . | 93 | . | . | . | 54 | 10 | . | . | . | . | . | . | . | . | . | . | . | . | . |
| Hagen et al. (2013) | 1 | . | . | 7 | . | . | 15 | 1 | . | 10 | . | . | . | . | . | . | . | . | . | . | . | 2 | . | . | . | . | . | . |
| Hasenauer et al. (2010) | 4 | . | . | 38 | . | 3 | 4 | . | . | 26 | . | . | . | . | . | . | . | . | . | . | . | 10 | 1 | 2 | . | . | . | . |
| Hasenauer (2013) | 16 | 4 | . | 210 | 1 | 49 | 1 | 2 | . | 256 | . | . | 1 | 37 | 10 | 2 | 2 | 2 | . | 1 | . | 7 | . | . | 10 | 1 | 1 | . |
| Mustafa et al. (2013) | . | . | . | 49 | . | . | . | . | . | 1 | . | . | . | . | . | 1 | 3 | . | . | . | . | 2 | . | . | 1 | . | 7 | . |
| Th and Manini (2008) | . | . | . | 19 | . | . | . | . | . | 25 | . | 17 | . | . | . | . | . | . | . | . | . | 1 | . | . | . | . | . | . |
| Hussain et al. (2015) | . | . | . | 48 | 3 | . | . | . | . | 4 | . | 2 | . | . | . | 6 | 1 | . | . | . | . | 3 | 13 | . | . | . | . | 1 |
| Hussain (2016) | 1 | . | . | 94 | 3 | . | . | . | . | 5 | . | . | . | . | 1 | 5 | 2 | . | . | 4 | 6 | 19 | 30 | . | . | . | . | 2 |
| Iwata et al. (2014) | 1 | . | . | 32 | . | . | . | . | 3 | . | . | . | . | . | . | . | . | . | . | 4 | 1 | . | . | . | . | . | . | . |
| Jagiella et al. (2017) | 2 | . | . | 53 | . | 11 | . | . | . | 18 | 86 | . | . | . | 1 | 65 | . | . | . | 1 | . | 1 | . | 4 | 6 | . | . | . |
| Jang et al. (2016) | 2 | . | . | 69 | . | . | . | . | . | 82 | . | . | . | . | . | . | . | . | . | 1 | . | 1 | . | . | . | . | . | . |
| Jaqaman and Danuser (2006) | 15 | . | . | 38 | . | . | . | . | . | 21 | . | . | . | . | . | . | . | . | . | 68 | . | . | . | . | . | . | . | . |
| Ji and Brown (2009) | 1 | . | . | 130 | 1 | . | . | . | . | 4 | . | . | . | . | . | . | 36 | 109 | 3 | . | . | . | . | 16 | . | . | 1 | . |
| Jia et al. (2011) | 8 | . | . | 77 | . | . | . | . | . | 2 | . | . | . | . | . | . | . | . | . | . | . | 1 | . | . | 2 | . | 1 | . |
| Joshia et al. (2006) | 1 | . | . | 23 | . | 33 | . | . | . | . | . | . | . | . | . | . | . | . | . | . | . | . | . | . | . | . | . | . |
| Karnaukhov et al. (2007) | . | . | . | 38 | . | . | . | . | . | . | . | . | . | . | . | . | . | . | . | . | . | . | . | . | . | . | . | . |
| Karimi and Mcauley (2013) | . | . | . | 58 | . | 4 | . | . | 1 | 127 | . | 30 | . | 13 | . | 4 | 2 | 9 | . | . | . | 4 | 2 | 2 | . | . | . | 1 |
| Karimi and Mcauley (2014b) | . | . | . | 64 | . | 9 | . | . | . | 214 | . | 7 | . | 8 | . | 4 | 4 | 5 | . | . | . | 2 | 1 | . | . | . | . | . |
| Karimi and Mcauley (2014a) | . | . | . | 40 | . | 7 | . | . | . | 115 | . | 20 | . | 17 | . | 1 | 7 | 8 | 1 | . | . | 2 | . | 2 | . | . | 1 | . |
| Kimura et al. (2015) | . | 2 | . | 33 | . | . | . | . | 21 | 123 | 5 | . | . | . | 9 | 1 | 1 | . | . | 13 | 2 | . | 3 | . | . | . | . | . |
| Kleinstein et al. (2006) | . | . | . | 14 | . | . | . | . | 3 | 3 | . | . | . | . | . | . | . | . | . | . | . | 1 | 3 | 29 | . | . | . | . |
| Ko et al. (2009) | 1 | . | . | 71 | 6 | . | 1 | . | . | 1 | . | . | . | . | . | . | . | . | . | 3 | 2 | . | 1 | 5 | . | . | . | . |
| Koblents and Míguez (2011) | . | . | . | 13 | . | . | . | . | . | 42 | . | . | . | . | 4 | . | . | . | . | . | . | 3 | . | 1 | 1 | . | . | . |
| Koblents and Míguez (2014) | . | . | . | 27 | . | . | . | . | . | 48 | . | . | . | 99 | 9 | 21 | . | . | . | . | . | 2 | . | . | 1 | . | . | . |
| **?** | . | . | . | 30 | . | . | . | . | . | 56 | 6 | . | . | 109 | 10 | 22 | . | . | . | . | . | . | . | . | 1 | . | . | . |
| Koeppl et al. (2010) | 3 | . | . | 9 | . | . | . | . | . | 3 | . | 1 | 3 | . | . | . | . | . | . | . | . | 1 | . | . | 4 | . | . | . |
| Koeppl et al. (2012) | . | . | . | 28 | . | . | . | . | . | 30 | . | 1 | 3 | 6 | 6 | . | . | . | . | 2 | . | 1 | . | . | 4 | . | . | . |
| Komorowski et al. (2009) | 1 | . | . | 49 | . | . | . | . | . | 29 | . | . | . | 1 | 1 | . | 1 | . | . | . | . | 1 | 1 | 1 | 1 | . | . | . |
| Komorowski et al. (2011) | 12 | . | . | 6 | 5 | 1 | 6 | . | . | 11 | . | . | . | . | . | . | . | . | . | . | . | . | . | . | . | . | . | . |
| Kravaris et al. (2013) | 10 | . | . | 120 | 13 | . | 4 | . | . | 4 | . | . | . | . | . | 2 | 4 | 3 | . | 16 | 3 | 2 | . | . | . | . | . | . |
| Kuepfer et al. (2007) | . | . | . | 22 | . | . | . | . | . | 3 | . | . | . | . | . | . | . | . | . | 1 | . | 9 | . | . | . | . | . | . |
| Kügler (2012) | 11 | . | . | 62 | 1 | . | . | . | . | 14 | . | . | . | 4 | 3 | . | 2 | . | . | 3 | . | . | . | 1 | . | . | . | . |
| Kulikov and Kulikova (2015a) | . | . | . | 67 | 1 | . | . | . | . | 3 | . | . | . | . | . | . | 38 | 171 | 2 | . | . | 1 | . | . | . | . | . | . |
| Kulikov and Kulikova (2015b) | . | . | . | 32 | . | . | . | . | . | . | . | . | . | . | . | . | 34 | 78 | 25 | . | . | 1 | . | . | . | . | 1 | . |
| Kulikov and Kulikova (2017) | . | . | . | 61 | 1 | . | . | . | . | 2 | . | . | . | . | . | . | 36 | 77 | 28 | . | . | 1 | . | . | . | . | 8 | . |
| Kuntz et al. (2017) | . | 1 | . | 5 | . | . | . | . | . | 1 | . | . | . | . | . | . | . | . | . | . | . | 17 | . | . | 2 | . | . | . |
| Kurt et al. (2016) | . | . | . | 148 | . | 2 | 34 | 103 | . | 73 | . | 1 | . | . | . | . | . | . | . | 6 | . | 1 | 1 | 2 | 1 | . | 1 | . |
| Kutalik et al. (2007) | 1 | . | . | 34 | . | . | . | . | . | . | . | . | . | . | . | . | . | . | . | 2 | 1 | . | 2 | 1 | . | . | . | . |
| Kuwahara et al. (2013) | . | 1 | . | 68 | . | . | . | . | . | 2 | . | 1 | . | . | . | 1 | 6 | 20 | . | . | 1 | 1 | 5 | . | . | . | . | . |
| Kyriakopoulos and Wolf (2015) | 2 | . | . | 14 | . | 2 | 19 | . | . | 7 | . | . | . | . | . | . | . | . | . | . | . | . | . | . | 1 | . | . | . |
| Lakatos et al. (2015) | 1 | . | . | 19 | 1 | . | . | 1 | . | 3 | 2 | . | . | . | . | . | . | . | . | . | . | 1 | . | . | 6 | . | . | . |
| Lakatos (2017) | 30 | 2 | . | 61 | 7 | . | . | 2 | . | 14 | 24 | 3 | . | . | . | 7 | . | . | . | . | . | 3 | . | . | 58 | . | . | . |
| Lang and Stelling (2016) | 4 | . | . | 56 | . | . | . | . | . | 4 | . | . | . | . | . | . | . | . | . | . | . | 4 | . | . | 1 | . | . | . |
| Lecca et al. (2009) | . | . | . | 24 | . | . | . | . | . | 10 | . | . | . | . | . | . | . | . | . | 4 | 3 | 2 | . | . | 1 | . | 1 | 1 |

**Table S2.** Coverage of parameter estimation strategies for BRNs. (cont.)

| | Tasks | | | | Measures | | | | | Bayesian methods | | | | Monte Carlo | | | Kalman filter | | | Model fitting | | | | | | | | XLR |
|---|---|---|---|---|---|---|---|---|---|---|---|---|---|---|---|---|---|---|---|---|---|---|---|---|---|---|---|---|
| Reference | identifi., observab., reachability | optimum experiment design | bifurcation analysis | inference, identification | sensitivity analysis | confidence/credible intervals | Akaike/Fisher/mutual info. | entropy | sum of squared errors | MAP, ML, likelihood | approximate Bayesian comput. | expectation-maximization | variational Bayesian inference | MCMC | Metropol./import. sampling | sequential MC, particle filters | Kalman filter | extended Kalman filter | unscented Kalman filter | LS and regression | genetic algorithms | optimization programming | simulated annealing | differential evolution | scatter, tabu, cuckoo search | particle swarm optimization | other algorithms | mach./deep/transf. learning |
| Li and Vu (2013) | 37 | . | . | 48 | 2 | . | 1 | . | . | 7 | . | . | . | . | . | . | . | . | . | . | 2 | 2 | 2 | . | . | . | . | . |
| Li and Vu (2015) | 72 | . | . | 40 | . | . | . | . | . | 4 | . | . | . | . | . | . | . | . | . | . | . | 1 | . | . | . | . | . | . |
| Liao et al. (2015a) | 9 | 29 | . | 29 | 1 | . | . | . | . | 7 | . | 2 | . | . | . | . | . | . | . | 1 | . | 3 | . | . | . | . | . | . |
| Liao (2017) | 13 | 40 | . | 34 | 7 | . | . | . | 3 | 8 | . | . | 1 | . | . | . | . | . | . | 1 | . | 2 | . | . | . | . | 2 | . |
| Liepe et al. (2014) | 2 | 1 | . | 67 | 1 | . | . | 1 | . | 78 | 129 | . | . | 4 | 1 | 30 | . | . | . | 1 | . | 5 | 1 | . | 1 | . | . | . |
| Lillacci and Khammash (2010b) | 11 | . | . | 93 | . | . | . | . | . | 20 | . | . | . | . | . | 2 | 46 | 41 | 1 | . | 8 | 1 | 2 | . | 1 | . | 1 | . |
| Lillacci and Khammash (2012) | 20 | . | . | 56 | . | . | . | . | . | 21 | . | . | . | . | 1 | 5 | 9 | 6 | . | . | 2 | . | 3 | . | . | . | . | . |
| Linder (2013) | 7 | . | . | 73 | . | . | . | . | . | 40 | . | 9 | . | 6 | . | . | . | . | . | 6 | . | . | . | 5 | 1 | . | . | . |
| Lindera and Rempala (2015) | 3 | . | . | 12 | . | . | . | . | . | 8 | . | 4 | . | . | . | . | . | . | . | 4 | . | . | . | . | . | . | . | . |
| Liu et al. (2006) | 2 | . | . | 14 | . | . | . | . | . | . | . | . | . | . | . | . | . | . | . | . | . | . | 1 | 1 | . | . | 1 | . |
| Liu and Wang (2008a) | . | . | . | 81 | . | . | . | . | . | . | . | . | . | . | . | . | . | . | . | 5 | 3 | 2 | 2 | 6 | 1 | . | 1 | . |
| Liu and Wang (2008b) | . | . | . | 58 | . | . | . | . | . | . | . | . | . | . | . | . | . | . | . | 4 | . | 1 | . | 6 | . | . | 2 | . |
| Liu and Wang (2009) | . | . | . | 28 | 2 | . | . | . | . | . | . | . | . | . | . | . | . | . | . | 3 | 1 | 2 | 2 | 45 | 3 | . | . | . |
| Liu et al. (2012) | . | . | . | 96 | . | . | . | . | . | . | . | 1 | . | . | . | . | . | . | . | 7 | 14 | 2 | 1 | 1 | . | 3 | 4 | . |
| Liu and Gunawan (2014) | 2 | . | . | 80 | . | . | . | . | . | . | . | . | . | . | . | . | . | . | . | 1 | . | 1 | . | 1 | 4 | . | . | . |
| Liu (2014) | 6 | . | . | 477 | 121 | . | 3 | . | . | 293 | 525 | 46 | 2 | 97 | 22 | 148 | 64 | 169 | 155 | 42 | . | . | 7 | . | 4 | . | 460 | 5 |
| Loos et al. (2016) | . | . | . | 18 | 5 | . | . | . | . | 50 | . | 3 | . | . | . | . | . | . | . | . | . | . | . | . | 1 | 2 | . | 1 |
| Lötstedt (2018) | . | . | . | 6 | . | . | . | . | . | 1 | . | 3 | . | . | . | . | . | . | . | 1 | . | . | 1 | . | . | . | 2 | . |
| Lück and Wolf (2016) | . | . | . | 63 | . | . | . | . | . | 25 | . | . | . | . | . | . | . | . | . | . | . | . | . | . | 1 | . | . | . |
| Mancini et al. (2015) | 5 | . | . | 20 | . | . | . | . | . | 1 | . | . | . | . | . | 11 | . | . | . | . | . | 1 | 1 | . | 1 | . | . | . |
| Mannakee et al. (2016) | 1 | . | . | 21 | 16 | 7 | 3 | . | . | 28 | 9 | . | . | 17 | 3 | 5 | . | . | . | 7 | . | 2 | 1 | 1 | . | . | . | . |
| Mansouri et al. (2014) | . | . | . | 163 | 1 | . | . | . | . | 35 | . | 2 | 21 | . | 4 | 4 | 22 | 53 | 46 | 2 | . | 2 | 1 | . | 1 | 2 | . | 1 |
| Mansouri et al. (2015) | . | . | . | 58 | 1 | . | . | . | . | 9 | . | 1 | . | . | 3 | 3 | 3 | 6 | 3 | 3 | . | 2 | 2 | . | 1 | 2 | . | . |
| Matsubara et al. (2006) | . | . | . | 32 | . | . | . | . | . | 2 | . | 1 | . | . | . | . | . | . | . | . | 13 | 5 | 1 | 3 | . | . | 7 | . |
| Mazur (2012) | 24 | 1 | 11 | 234 | 10 | . | 25 | 185 | . | 137 | . | 9 | . | 44 | 31 | 4 | . | . | . | 5 | 18 | 67 | 1 | 2 | 4 | . | 2 | 2 |
| Mazur and Kaderali (2013) | 5 | . | 1 | 29 | . | . | 1 | 7 | . | 22 | . | . | 1 | 16 | 2 | . | . | . | . | . | . | . | . | . | 1 | . | . | . |
| McGoff et al. (2015) | 15 | . | . | 200 | . | 1 | . | 6 | . | 83 | 14 | . | . | 4 | 1 | 16 | 16 | 7 | 7 | 15 | . | . | . | . | . | . | 11 | . |
| Mendes and Kell (1998) | . | 1 | . | 17 | . | 1 | . | . | . | . | . | . | . | . | . | . | . | . | . | 3 | 7 | 21 | 10 | . | . | . | . | . |
| Meskin et al. (2011) | 1 | . | . | 43 | . | . | . | . | . | . | . | . | . | . | . | . | 12 | 46 | . | 5 | 2 | 3 | 2 | . | 1 | . | . | . |
| Meskin et al. (2013) | 4 | . | . | 60 | . | . | . | . | . | . | . | . | . | . | . | . | 15 | 3 | 66 | 6 | 1 | 2 | 3 | . | 1 | . | . | . |
| Michailidis and d'Alché Buc (2013) | . | . | . | 83 | . | . | . | 1 | . | 8 | . | . | . | 1 | . | . | . | . | . | 24 | . | 2 | . | . | . | . | . | . |
| Michalik et al. (2009) | 4 | . | 1 | 94 | . | . | . | . | . | 4 | . | . | . | . | . | . | . | . | . | . | . | 3 | . | . | . | . | . | . |
| Mihaylova et al. (2012) | . | . | . | 33 | . | . | . | . | . | 14 | . | 1 | . | 1 | 3 | 12 | 5 | 4 | 3 | . | . | 2 | . | . | 1 | . | . | . |
| Mihaylova et al. (2014) | . | . | . | 51 | . | 1 | . | . | . | 39 | . | . | 3 | 52 | 6 | 65 | 8 | 1 | 2 | . | . | . | . | . | . | . | . | . |
| Mikeev and Wolf (2012) | . | . | . | 35 | . | . | 3 | . | . | 42 | . | 1 | . | . | . | . | . | . | . | . | . | 1 | . | 1 | . | . | . | . |
| Mikelson and Khammash (2016) | 1 | 1 | . | 61 | . | . | . | . | . | 191 | . | . | . | 2 | . | . | . | . | . | . | . | . | 1 | . | 1 | . | . | 1 |
| Milios et al. (2018) | 17 | . | . | 26 | . | . | . | . | . | 18 | . | . | . | . | . | 16 | . | . | . | . | . | . | . | . | . | . | . | 4 |
| Milner et al. (2013) | . | . | . | 23 | . | . | . | . | . | 29 | 4 | . | . | 11 | 5 | 1 | . | . | . | . | . | . | . | . | 1 | . | . | . |
| Mizera et al. (2014) | . | . | . | 27 | . | . | . | . | . | 4 | . | . | . | . | . | . | . | . | . | . | . | . | . | . | . | . | . | . |
| Moles et al. (2003) | . | . | . | 20 | . | . | . | . | . | 4 | . | . | 1 | . | . | . | . | . | . | . | 5 | 9 | 7 | 7 | . | . | . | 1 |
| Jaime and Denis (2015) | 25 | . | . | 2 | . | . | . | . | . | . | . | . | . | . | . | . | . | . | . | . | . | 1 | . | 3 | . | . | . | . |
| Moritz (2014) | 30 | . | . | 172 | . | . | . | 1 | . | 30 | . | . | . | . | . | . | . | . | . | . | . | 7 | . | . | 2 | . | . | . |
| Mozgunov et al. (2018) | . | . | . | 3 | . | . | . | 1 | . | . | . | . | . | . | . | . | . | . | . | . | . | 1 | . | . | . | . | . | . |
| Mu (2010) | . | . | . | 185 | 2 | . | 1 | 1 | 4 | . | . | . | . | . | . | . | 1 | 1 | . | 19 | 4 | 1 | 1 | . | . | . | 2 | . |
| Müller et al. (2012) | 1 | . | . | 48 | . | . | . | 4 | . | 13 | 19 | . | 2 | 11 | 2 | 2 | . | . | . | . | 2 | . | . | 1 | 2 | . | . | . |
| Murakami (2014) | 2 | 2 | . | 80 | . | . | . | . | 1 | 119 | 98 | 1 | . | 13 | 4 | 10 | . | . | . | . | . | . | 4 | . | . | . | . | . |
| Nemeth et al. (2014) | . | . | . | 67 | . | . | . | . | . | 40 | . | 1 | . | 2 | 2 | 26 | 3 | . | 1 | . | . | 1 | . | . | 1 | . | . | . |
| Nienaltowski et al. (2015) | 48 | . | . | 11 | 5 | . | 7 | 5 | . | 22 | . | 1 | . | . | . | . | . | . | . | . | . | 9 | . | 1 | . | . | . | . |
| Nim et al. (2013) | . | . | . | 62 | . | . | . | . | 8 | 10 | . | . | . | . | . | . | . | . | . | 2 | 2 | 4 | . | . | . | 4 | 1 | . |

**Table S2.** Coverage of parameter estimation strategies for BRNs. (cont.)

| | Tasks | | | | Measures | | | | | Bayesian methods | | | | Monte Carlo | | | Kalman filter | | | Model fitting | | | | | | | | XLR |
|---|---|---|---|---|---|---|---|---|---|---|---|---|---|---|---|---|---|---|---|---|---|---|---|---|---|---|---|---|
| Reference | identifi., observab., reachability | optimum experiment design | bifurcation analysis | inference, identification | sensitivity analysis | confidence/credible intervals | Akaike/Fisher/mutual info. | entropy | sum of squared errors | MAP, ML, likelihood | approximate Bayesian comput. | expectation-maximization | variational Bayesian inference | MCMC | Metropol./import. sampling | sequential MC, particle filters | Kalman filter | extended Kalman filter | unscented Kalman filter | LS and regression | genetic algorithms | optimization programming | simulated annealing | differential evolution | scatter, tabu, cuckoo search | particle swarm optimization | other algorithms | mach./deep/transf. learning |
| Nobile et al. (2012) | . | . | . | 27 | . | . | . | . | . | . | . | . | . | . | . | . | . | . | . | . | 4 | . | . | . | 1 | 41 | 1 | . |
| Nobile et al. (2013) | . | . | . | 24 | . | . | 1 | . | . | 2 | . | . | . | . | . | . | . | . | . | . | 2 | 23 | . | . | 1 | 39 | 1 | . |
| Nobile et al. (2015) | . | . | . | 7 | . | . | . | . | . | . | . | . | . | . | . | . | . | . | . | . | 2 | 2 | . | 1 | . | 125 | 1 | . |
| Nobile et al. (2016) | . | . | . | 26 | 1 | . | . | . | . | . | . | . | . | . | . | . | . | . | . | . | 1 | 2 | . | . | . | 139 | 1 | . |
| Nobile et al. (2018a) | . | . | . | 19 | 2 | . | . | . | . | . | . | . | . | . | . | . | . | . | . | . | 5 | 2 | . | 13 | . | 237 | 14 | . |
| Nobile et al. (2018b) | . | . | . | 1 | 1 | . | . | . | . | 1 | . | . | . | . | . | . | . | . | . | . | 1 | 1 | . | 2 | . | 2 | 2 | . |
| Pahle et al. (2012) | 1 | 3 | . | 8 | 2 | . | 1 | . | . | 45 | . | . | . | . | . | . | . | . | . | . | . | 2 | 2 | . | 3 | 1 | 4 | . |
| Palmisano (2010) | 6 | 2 | . | 141 | . | . | 1 | . | . | 71 | 3 | . | . | 1 | 1 | 1 | . | . | . | 4 | 3 | 24 | 2 | 4 | 5 | . | . | 1 |
| Pan and Yang (2010) | . | . | . | 14 | . | . | 1 | . | . | 20 | . | . | . | . | 2 | . | . | . | . | 12 | . | 3 | . | . | . | . | . | 145 |
| Pantazis et al. (2013) | 13 | . | . | 18 | 49 | . | 8 | 47 | . | 7 | . | . | 1 | . | . | . | . | . | . | . | . | 1 | . | 3 | . | . | . | . |
| Paul (2014) | . | 1 | . | 25 | . | . | . | . | . | 29 | . | . | . | 42 | 5 | . | . | . | . | . | . | 1 | . | . | . | . | . | . |
| Penas et al. (2017) | . | . | . | 39 | . | 1 | . | . | . | 1 | . | . | . | . | . | . | . | . | . | . | . | 10 | 1 | 9 | 39 | 1 | . | . |
| Plesa et al. (2017) | . | 87 | . | 19 | . | . | . | . | . | . | . | . | . | . | . | . | . | . | . | . | . | 1 | . | . | . | . | . | . |
| Poovathingal and Gunawan (2010) | 6 | 1 | . | 63 | 2 | . | . | . | . | 40 | . | 1 | . | . | . | . | . | . | . | . | . | . | . | 13 | . | . | . | . |
| Pullen and Morris (2014) | 1 | 1 | . | 46 | . | . | 2 | 1 | . | 77 | . | . | . | 20 | . | 1 | 1 | . | . | 3 | 1 | 1 | 6 | . | . | 1 | 2 | 2 |
| Quach et al. (2007) | . | . | . | 52 | . | 1 | . | . | . | 10 | . | . | . | 1 | . | 1 | 13 | 3 | 19 | 2 | . | . | 1 | . | . | . | 1 | . |
| Radulescu et al. (2012) | 3 | . | . | 13 | 1 | . | . | 3 | . | 1 | . | 5 | . | . | . | . | . | . | . | . | . | 2 | . | 1 | 1 | . | . | 3 |
| Rakhshania et al. (2016) | . | . | . | 32 | . | 4 | . | . | . | . | . | . | . | . | . | . | . | . | . | . | 4 | 2 | 3 | 8 | 26 | 22 | 22 | . |
| J. O. Ramsay and Cao (2007) | 9 | 10 | 2 | 171 | 1 | 4 | 3 | . | 1 | 43 | . | . | . | . | 2 | 4 | 7 | . | . | 27 | . | 2 | 1 | 7 | . | . | 1 | . |
| Rapaport and Dochain (2005) | . | . | . | 16 | . | . | . | . | . | . | . | . | . | . | . | . | 1 | . | . | . | . | . | . | . | . | . | . | . |
| Reinker et al. (2006) | 1 | 1 | . | 32 | . | 7 | 2 | . | . | 46 | . | . | . | . | . | . | . | . | . | . | . | . | . | . | . | . | . | . |
| Reis et al. (2018) | . | . | . | 4 | . | . | . | 1 | . | 2 | . | . | . | . | . | . | . | . | . | . | . | . | . | . | . | . | . | . |
| Remlia et al. (2017) | 1 | . | . | 56 | . | . | . | . | . | 8 | . | . | . | . | . | . | . | . | . | . | . | 3 | 3 | 35 | 19 | 36 | 3 | 1 |
| Rempala (2012) | 7 | . | . | 19 | 1 | . | 2 | . | . | . | . | . | . | . | . | . | . | . | . | 3 | . | . | . | . | . | . | . | . |
| Revell and Zuliani (2018) | 1 | 1 | . | 44 | . | . | . | 26 | . | 17 | 4 | 3 | . | . | 1 | 5 | . | . | . | . | 2 | 3 | . | . | . | . | . | . |
| Rosati et al. (2018) | . | . | . | 4 | . | . | . | . | . | 3 | . | . | . | . | . | . | . | . | . | . | . | 4 | 1 | 1 | . | . | . | . |
| Ruess et al. (2011) | 5 | . | . | 15 | . | . | . | . | . | 7 | . | . | . | . | . | . | 29 | 9 | . | . | . | 1 | . | . | . | . | . | . |
| Ruess (2014) | 5 | 1 | . | 120 | . | 1 | 41 | . | . | 115 | . | . | 1 | 5 | 2 | . | 14 | 4 | . | 2 | . | 4 | 2 | . | 13 | . | . | . |
| Ruess and Lygeros (2015) | 4 | . | . | 64 | . | . | 23 | . | . | 28 | . | . | . | 4 | . | . | 1 | 1 | . | 1 | . | . | . | 1 | . | . | . | . |
| Rumschinski et al. (2010) | 3 | . | . | 44 | 3 | . | . | . | . | 1 | . | . | . | . | . | . | . | . | . | . | 1 | 13 | 1 | 6 | . | . | . | . |
| Ruttor and Opper (2009) | . | . | . | 16 | . | . | . | . | . | 22 | . | . | . | . | 1 | . | 3 | . | . | . | . | . | 1 | 4 | . | . | 1 | . |
| Sadamoto et al. (2017) | . | . | . | 51 | . | . | . | . | . | . | . | 1 | . | . | . | . | 8 | . | . | . | . | . | . | 5 | . | . | . | . |
| Sagar et al. (2017) | . | . | . | 33 | . | . | . | . | . | . | . | . | . | . | . | . | . | . | . | 1 | 7 | 2 | 8 | 9 | 5 | 27 | . | . |
| Schenkendorf (2014) | 10 | 1 | 1 | 237 | 23 | 28 | 12 | 8 | 4 | 23 | . | 4 | 1 | . | . | . | 29 | 9 | 24 | 6 | 3 | 1 | 1 | 3 | 1 | . | 8 | . |
| Schilling et al. (2016) | . | . | . | 53 | . | . | . | 1 | . | 34 | . | . | . | . | 1 | . | 1 | 1 | . | . | . | 2 | . | . | 1 | . | . | . |
| Schnoerr (2016) | . | . | . | 90 | . | . | . | . | 2 | 67 | . | . | . | . | . | . | . | . | . | . | . | 2 | . | . | 4 | . | . | . |
| Schnoerr et al. (2017) | . | 2 | . | 69 | . | . | . | 10 | 9 | 25 | . | . | 5 | 5 | . | . | 1 | . | . | . | . | 4 | . | . | 17 | . | 3 | 3 |
| Septier and Peters (2016) | . | . | . | 22 | . | . | . | . | . | 39 | . | . | . | 130 | 22 | 111 | 14 | 4 | 3 | . | . | . | 1 | . | 1 | . | 1 | . |
| Shacham and Brauner (2014) | 3 | . | . | 53 | . | 22 | . | . | . | 1 | . | . | . | . | . | . | . | . | . | 62 | . | 1 | . | 1 | . | . | 2 | . |
| Sherlock et al. (2014) | . | . | . | 40 | . | . | . | . | . | 21 | . | . | . | 8 | 4 | 1 | . | . | . | . | . | . | . | . | . | . | . | . |
| Shiang (2009) | . | 1 | . | 54 | . | 1 | 1 | . | . | 4 | . | . | . | . | . | . | . | . | . | 3 | . | 24 | 2 | . | . | . | . | . |
| Zamora-Sillero et al. (2011) | . | 3 | . | 15 | 2 | . | . | . | . | . | . | 1 | . | 1 | 8 | . | . | . | . | . | . | 1 | 7 | 1 | . | 1 | . | . |
| Singh and Hahn (2005) | 34 | . | . | 20 | . | . | . | . | . | . | . | . | . | . | . | . | 2 | . | . | . | . | . | . | . | . | . | . | . |
| Slezak et al. (2010) | 1 | . | . | 16 | . | . | . | 1 | . | . | . | . | . | . | . | . | . | . | . | 1 | . | 2 | . | . | . | . | . | 1 |
| Smadbeck (2014) | . | 4 | . | 4 | 7 | . | . | 23 | . | 2 | . | 2 | . | . | . | . | 2 | 1 | . | 5 | . | 8 | 3 | 1 | . | . | . | . |
| Smet and Marchal (2010) | . | . | . | 129 | . | . | 2 | . | . | 8 | . | . | . | . | . | . | . | . | . | 4 | . | 26 | . | . | 1 | . | . | . |
| Smith and Grima (2018) | . | . | . | 2 | . | . | . | . | . | 2 | . | 1 | 1 | . | . | . | . | . | . | . | . | 1 | 5 | . | 1 | . | . | . |
| He et al. (2004) | . | . | . | 20 | . | . | . | . | . | 3 | . | 1 | . | . | . | . | . | . | . | 1 | . | 9 | . | . | . | . | . | . |
| Srinath and Gunawan (2010) | 85 | . | . | 41 | 3 | 5 | 1 | . | . | 3 | . | . | . | . | . | . | . | . | . | 3 | . | . | . | 1 | . | . | . | . |

**Table S2.** Coverage of parameter estimation strategies for BRNs. (cont.)

| | Tasks | | | | Measures | | | | | Bayesian methods | | | | Monte Carlo | | | Kalman filter | | | Model fitting | | | | | | | | XLR |
|---|---|---|---|---|---|---|---|---|---|---|---|---|---|---|---|---|---|---|---|---|---|---|---|---|---|---|---|---|
| Reference | identifi., observab., reachability | optimum experiment design | bifurcation analysis | inference, identification | sensitivity analysis | confidence/credible intervals | Akaike/Fisher/mutual info. | entropy | sum of squared errors | MAP, ML, likelihood | approximate Bayesian comput. | expectation-maximization | variational Bayesian inference | MCMC | Metropol./import. sampling | sequential MC, particle filters | Kalman filter | extended Kalman filter | unscented Kalman filter | LS and regression | genetic algorithms | optimization programming | simulated annealing | differential evolution | scatter, tabu, cuckoo search | particle swarm optimization | other algorithms | mach./deep/transf. learning |
| Srinivas and Rangaiah (2007) | . | . | . | 31 | . | . | . | . | 1 | . | . | . | . | . | . | . | . | . | . | . | 2 | 7 | 1 | 227 | 45 | 3 | . | . |
| Srivastava (2012) | 1 | 2 | . | 89 | 6 | 3 | . | . | . | 78 | . | . | 1 | . | 5 | 2 | . | . | . | 2 | 1 | 7 | . | . | 3 | . | . | . |
| Srivastavaa and Rawlingsb (2014) | . | 1 | . | 43 | . | 4 | . | . | . | 43 | . | 1 | 1 | . | . | . | . | . | . | 1 | 1 | 7 | . | . | 1 | . | . | . |
| von Stosch et al. (2014) | 2 | . | . | 96 | . | 3 | 1 | . | . | 5 | . | . | . | . | . | . | 1 | 3 | . | 3 | 1 | 1 | . | . | 1 | . | 48 | . |
| Emmert-Streib et al. (2012) | . | . | . | 83 | . | . | 48 | 3 | . | 5 | . | . | . | . | . | . | . | . | . | . | . | . | 5 | 2 | . | . | . | . |
| Sun et al. (2008) | . | 1 | . | 70 | . | . | . | . | . | 17 | . | 2 | . | . | . | 5 | 19 | 48 | 10 | 1 | . | 1 | 1 | . | . | . | . | . |
| Sun et al. (2012) | 7 | . | 1 | 119 | . | . | 2 | . | . | 4 | . | 1 | . | . | . | . | . | . | . | 9 | 26 | 31 | 20 | 28 | 2 | 5 | 4 | 3 |
| Sun et al. (2014) | . | . | . | 39 | . | . | . | . | . | 1 | . | . | . | . | . | . | . | . | . | 7 | 4 | 12 | 3 | 9 | 3 | 118 | 3 | . |
| Swaminathan and Murray (2014) | 2 | . | . | 23 | . | . | . | 1 | . | 15 | . | . | . | . | . | . | . | . | . | . | . | 3 | . | . | . | . | . | 2 |
| Tanevski et al. (2010) | . | 1 | . | 54 | . | . | . | . | . | 57 | 32 | . | . | 3 | 4 | 8 | . | . | . | . | . | . | . | . | . | . | . | . |
| Tangherloni et al. (2016) | . | . | . | 15 | 1 | . | . | . | . | . | . | . | . | . | . | . | . | . | . | . | 1 | 1 | . | 1 | . | 43 | . | . |
| Teijeiro et al. (2017) | . | . | . | 25 | . | . | . | . | . | . | . | . | . | . | . | . | . | . | . | . | . | 14 | . | 94 | 14 | . | . | . |
| Tenazinha and Vinga (2011) | 12 | 3 | . | 13 | 1 | . | . | . | . | 1 | . | 11 | . | . | . | . | . | . | . | . | . | 6 | 1 | . | . | . | . | . |
| Thomas et al. (2012) | . | 2 | . | 1 | . | . | . | . | . | . | . | . | . | . | . | . | . | . | . | . | . | . | . | . | 3 | . | 4 | . |
| Tian et al. (2007) | . | 1 | . | 66 | . | . | . | . | . | 24 | . | . | . | . | . | . | . | . | . | . | 14 | 8 | . | . | . | . | . | 2 |
| Tian et al. (2010) | . | . | . | 24 | 1 | . | . | . | . | . | . | . | . | . | . | . | . | . | . | . | . | 1 | . | 1 | . | . | . | . |
| Toni and Stumpf (2010) | 6 | . | . | 38 | 3 | 4 | 1 | . | . | 36 | 18 | . | . | 2 | . | 8 | 1 | . | 1 | . | 2 | . | 2 | . | 3 | . | . | . |
| Transtrum and Qiu (2012) | 2 | . | . | 21 | . | 6 | 27 | . | . | 5 | . | . | . | 2 | . | . | . | . | . | . | . | . | . | . | . | . | . | . |
| Siegal-Gaskins et al. (2015) | 12 | . | . | 26 | . | 8 | . | . | . | 3 | . | . | . | 3 | . | . | . | . | . | . | . | 1 | 1 | . | 1 | . | . | . |
| Vanlier et al. (2013) | 19 | . | . | 39 | 6 | 14 | . | . | . | 70 | 6 | . | . | 14 | 7 | 3 | . | . | . | 1 | . | 1 | . | . | . | . | . | 1 |
| Vargas et al. (2014) | 5 | . | . | 37 | . | . | . | . | . | . | . | . | . | . | . | . | 2 | 3 | . | . | . | 1 | . | . | . | . | . | . |
| Veerman et al. (2018) | . | . | . | 30 | . | . | . | . | . | 18 | . | . | . | . | . | . | . | . | . | . | . | . | . | . | 16 | . | . | . |
| Venayak et al. (2018) | . | . | . | 1 | . | . | 1 | . | . | . | . | . | . | . | . | . | . | . | . | . | . | 4 | . | . | . | . | . | . |
| Villaverde et al. (2012) | 7 | . | . | 25 | 1 | . | . | . | . | 2 | . | . | . | . | . | . | . | . | . | 1 | 1 | 11 | 1 | 11 | 23 | . | . | . |
| Villaverde et al. (2014) | . | . | . | 59 | . | . | 41 | 56 | . | 11 | . | 5 | . | . | . | . | . | . | . | . | . | 1 | . | . | . | . | 1 | 1 |
| Villaverde et al. (2016) | 192 | . | . | 22 | . | . | . | . | . | 6 | . | . | . | . | . | . | 4 | 1 | . | 1 | . | 4 | . | . | . | . | . | . |
| Villaverde and Barreiro (2016) | 145 | . | . | 37 | 13 | 4 | 4 | . | . | 14 | . | . | . | . | . | . | 2 | . | . | 5 | . | 5 | 1 | . | 1 | . | . | . |
| Voit (2013) | 1 | 8 | . | 197 | 8 | . | . | 2 | . | 10 | . | . | 1 | 8 | . | 1 | 2 | 1 | . | 8 | 11 | 24 | 4 | 7 | 4 | 2 | 7 | 1 |
| Vrettas et al. (2011) | 1 | . | . | 136 | . | . | . | . | . | 124 | . | 4 | 59 | 7 | 3 | 1 | 24 | 4 | 7 | 6 | . | . | . | . | . | . | . | . |
| Wang et al. (2010) | . | 1 | . | 40 | 3 | . | . | . | . | 63 | . | 4 | . | 45 | 2 | 1 | . | . | . | . | 2 | 4 | 4 | . | 2 | . | . | . |
| Weber and Frey (2017) | . | . | . | 5 | . | . | . | 1 | . | . | . | . | 13 | . | 1 | . | . | . | . | . | . | 1 | . | . | 3 | . | 4 | . |
| Weiss et al. (2016) | . | . | . | 67 | . | . | . | 1 | . | 10 | . | . | . | . | . | . | . | . | . | 6 | . | 3 | . | . | 15 | . | 5 | 295 |
| Whitaker et al. (2017) | 2 | . | . | 62 | . | 1 | . | . | . | 54 | . | . | . | 8 | 18 | . | 2 | 2 | . | . | . | 1 | . | . | . | . | . | . |
| White et al. (2015) | . | . | . | 34 | . | . | . | . | . | 115 | 154 | 2 | . | 18 | . | 1 | . | . | . | 3 | . | 1 | . | . | . | . | . | . |
| White et al. (2016) | 17 | . | . | 48 | 1 | . | 1 | . | 2 | 5 | . | . | . | . | . | . | . | . | . | 3 | . | . | . | . | . | . | . | . |
| Wong et al. (2015) | 1 | . | . | 5 | . | . | . | . | . | 4 | . | . | . | . | . | . | . | . | . | 1 | . | 3 | . | . | . | . | . | . |
| Woodcock et al. (2011) | . | . | . | 41 | . | 1 | . | . | . | 32 | . | . | . | 5 | 6 | . | . | . | . | . | . | 1 | 1 | 1 | 4 | . | . | . |
| Xiong and Zhou (2013) | . | . | . | 67 | . | . | . | . | . | 2 | . | . | . | . | . | . | 15 | 46 | . | 1 | . | 4 | . | . | . | . | . | . |
| Yang et al. (2014) | . | . | . | 59 | 8 | . | . | . | . | 32 | 85 | . | 1 | . | 1 | 61 | 16 | 6 | 2 | . | 2 | 2 | . | . | 1 | . | . | . |
| Yang et al. (2012) | . | . | . | 25 | . | . | . | . | . | . | . | . | . | . | . | . | . | . | . | 7 | 1 | 2 | 1 | 1 | . | . | . | . |
| Yenkie et al. (2016) | . | . | . | 44 | . | . | . | . | . | 1 | . | 1 | . | . | . | . | . | . | . | . | . | 3 | . | . | . | . | . | . |
| Zechner et al. (2011) | . | . | . | 34 | . | . | . | . | . | 33 | . | . | . | 4 | 6 | 4 | . | . | . | . | . | 1 | . | . | . | . | . | . |
| Zechner et al. (2012) | 2 | . | . | 20 | 1 | . | 1 | 1 | . | 18 | . | . | 7 | . | 4 | 2 | . | . | . | 1 | . | 1 | . | 1 | 2 | . | . | . |
| Zechner (2014) | 9 | . | . | 191 | 1 | 3 | 1 | 1 | . | 156 | 2 | 2 | 18 | 8 | 14 | 13 | 2 | 1 | . | 3 | . | 4 | 1 | . | 40 | . | 2 | 2 |
| Zeng et al. (2012) | . | . | . | 35 | . | . | . | . | . | . | . | . | . | . | . | 1 | 24 | 57 | . | . | . | . | . | . | . | 47 | 2 | . |
| Zhan and Yeung (2011) | 4 | 2 | . | 65 | 3 | . | . | . | . | 3 | . | . | . | . | . | . | . | . | . | 1 | 3 | 15 | 5 | . | . | 1 | 1 | . |
| Zhan et al. (2014) | 4 | 1 | . | 68 | 2 | 1 | . | . | . | . | . | . | . | . | . | . | . | . | . | 1 | . | 7 | 1 | 35 | . | 2 | . | . |
| Zimmer et al. (2014) | 22 | . | . | 46 | . | 3 | . | . | . | 2 | . | . | . | . | . | . | . | . | . | . | . | . | . | . | . | 1 | 1 | . |
| Zimmer and Sahle (2012) | 2 | . | 2 | 112 | . | 4 | . | . | . | 9 | . | 1 | . | . | . | . | . | . | . | 1 | . | 1 | . | . | . | . | . | . |

**Table S2.** Coverage of parameter estimation strategies for BRNs. (cont.)

| | Tasks | | | | Measures | | | | | Bayesian methods | | | | Monte Carlo | | | Kalman filter | | | Model fitting | | | | | | | | XLR |
|---|---|---|---|---|---|---|---|---|---|---|---|---|---|---|---|---|---|---|---|---|---|---|---|---|---|---|---|---|
| Reference | identifi., observab., reachability | optimum experiment design | bifurcation analysis | inference, identification | sensitivity analysis | confidence/credible intervals | Akaike/Fisher/mutual info. | entropy | sum of squared errors | MAP, ML, likelihood | approximate Bayesian comput. | expectation-maximization | variational Bayesian inference | MCMC | Metropol./import. sampling | sequential MC, particle filters | Kalman filter | extended Kalman filter | unscented Kalman filter | LS and regression | genetic algorithms | optimization programming | simulated annealing | differential evolution | scatter, tabu, cuckoo search | particle swarm optimization | other algorithms | mach./deep/transf. learning |
| Zimmer and Sahle (2015) | 7 | . | . | 68 | . | 1 | . | . | . | 24 | . | . | . | . | . | . | 2 | . | . | . | . | . | . | . | . | . | . | . |
| Zimmer (2015) | 4 | . | . | 82 | . | . | . | . | . | 20 | . | . | . | 1 | 1 | . | 2 | . | . | 1 | . | . | . | . | . | 1 | 1 | . |
| Zimmer (2016) | 4 | . | 2 | 47 | . | 8 | 129 | . | . | 20 | . | . | . | . | . | . | 2 | . | . | . | . | . | . | 1 | . | . | . | . |
| **# papers** | 148 | 62 | 9 | 297 | 83 | 63 | 70 | 52 | 21 | 238 | 31 | 59 | 34 | 84 | 80 | 79 | 89 | 54 | 30 | 112 | 82 | 196 | 99 | 87 | 117 | 45 | 73 | 46 |

**Table S3.** The references with citation links to Google Scholar.

| | | | |
|---|---|---|---|
| Abdullah et al. (2013b) | Cited by | Famili et al. (2005) | Cited by |
| Abdullah et al. (2013a) | Cited by | Farina et al. (2006) | Cited by |
| Abdullah et al. (2013c) | Cited by | Fearnhead and Prangle (2012) | Cited by |
| Alberton et al. (2013) | Cited by | Fearnhead et al. (2014) | Cited by |
| Ale et al. (2013) | Cited by | Rodriguez-Fernandez et al. (2006b) | Cited by |
| Ali et al. (2015) | Cited by | Rodriguez-Fernandez et al. (2006a) | Cited by |
| Amrein and Künsch (2012) | Cited by | Rodriguez-Fernandez et al. (2013) | Cited by |
| Anai et al. (2006) | Cited by | Fey et al. (2008) | Cited by |
| Andreychenko et al. (2011) | Cited by | Fey and Bullinger (2010) | Cited by |
| Andreychenko et al. (2012) | Cited by | Folia and Rattray (2018) | Cited by |
| Andrieu et al. (2010) | Cited by | Fröhlich et al. (2014) | Cited by |
| Angius and Horváth (2011) | Cited by | Fröhlich et al. (2016) | Cited by |
| Arnold et al. (2014) | Cited by | Fröhlich et al. (2017) | Cited by |
| Ashyraliyev et al. (2009) | Cited by | Gábor and Banga (2014) | Cited by |
| Babtie and Stumpf (2017) | Cited by | Gábor et al. (2017) | Cited by |
| Backenköhler et al. (2016) | Cited by | Geffen et al. (2008) | Cited by |
| Baker et al. (133, 2010) | Cited by | Gennemark and Wedelin (2007) | Cited by |
| Baker et al. (2011) | Cited by | Georgieva et al. (2016) | Cited by |
| Baker et al. (2013) | Cited by | Ghusinga et al. (2017) | Cited by |
| Baker et al. (2015) | Cited by | Gillespie and Golightly (2012) | Cited by |
| Banga and Canto (2008) | Cited by | Golightly and Wilkinson (2005) | Cited by |
| Barnes et al. (2011) | Cited by | Golightly and Wilkinson (2006) | Cited by |
| Bayer et al. (2016) | Cited by | Golightly and Wilkinson (2011) | Cited by |
| Berrones et al. (2016) | Cited by | Golightly et al. (2012) | Cited by |
| Besozzi et al. (2009) | Cited by | Golightly and Wilkinson (2014) | Cited by |
| Bhaskar et al. (2010) | Cited by | Golightly et al. (2015) | Cited by |
| Blei et al. (2017) | Cited by | Golightly and Kypraios (2017) | Cited by |
| Bogomolov et al. (2015) | Cited by | González et al. (2013) | Cited by |
| Bouraoui et al. (2015) | Cited by | Gordon et al. (1993) | Cited by |
| Farza et al. (2016) | Cited by | Guillén-Gosálbez et al. (2013) | Cited by |
| Boys et al. (2008) | Cited by | Gratie et al. (2013) | Cited by |
| Bronstein et al. (2015) | Cited by | Gupta (2013) | Cited by |
| Brunel et al. (2014) | Cited by | Gupta and Rawlings (2014) | Cited by |
| Busetto and Buhmann (2009) | Cited by | Hagen et al. (2013) | Cited by |
| Camacho et al. (2018) | Cited by | Hasenauer et al. (2010) | Cited by |
| Balsa-Canto et al. (2008) | Cited by | Hasenauer (2013) | Cited by |
| Carmi et al. (2013) | Cited by | Mustafa et al. (2013) | Cited by |
| Cazzaniga et al. (2015) | Cited by | Th and Manini (2008) | Cited by |
| Cedersund et al. (2016) | Cited by | Hussain et al. (2015) | Cited by |
| Česka et al. (2014) | Cited by | Iwata et al. (2014) | Cited by |
| Češka et al. (2017) | Cited by | Jagiella et al. (2017) | Cited by |
| Chen et al. (2015) | Cited by | Jaqaman and Danuser (2006) | Cited by |
| Chevaliera and Samadb (2011) | Cited by | Ji and Brown (2009) | Cited by |
| Chong et al. (2012) | Cited by | Jia et al. (2011) | Cited by |
| Chong et al. (2014) | Cited by | Joshia et al. (2006) | Cited by |
| Chou et al. (2006) | Cited by | Karnaukhov et al. (2007) | Cited by |
| Chou and Voit (2009) | Cited by | Karimi and Mcauley (2013) | Cited by |
| Cseke et al. (2016) | Cited by | Karimi and Mcauley (2014a) | Cited by |
| Dai and Lai (2010) | Cited by | Karimi and Mcauley (2014b) | Cited by |
| Daigle et al. (2012) | Cited by | Kimura et al. (2015) | Cited by |
| Jr. et al. (2012) | Cited by | Klein et al. (2011) | Cited by |
| Dattner (2015) | Cited by | Kleinstein et al. (2006) | Cited by |
| Deng and Tian (2014) | Cited by | Ko et al. (2009) | Cited by |
| Dinh and Sidje (2017) | Cited by | Koblents and Míguez (2011) | Cited by |
| Dochain (2003) | Cited by | Koblents and Míguez (2014) | Cited by |
| Drovandi et al. (2016) | Cited by | Koeppl et al. (2012) | Cited by |
| Eghtesadi and Mcauley (2014) | Cited by | Komorowski et al. (2009) | Cited by |
| Eisenberg and Hayashi (2014) | Cited by | Komorowski et al. (2011) | Cited by |
| Engl et al. (2009) | Cited by | Kravaris et al. (2013) | Cited by |
| Erguler and Stumpf (2011) | Cited by | Kuepfer et al. (2007) | Cited by |
| Fages et al. (2015) | Cited by | Kügler (2012) | Cited by |

**Table S3.** The references with citation links in Google Scholar. (cont.)

| | | | |
|---|---|---|---|
| | | Pantazis et al. (2013) | Cited by |
| Kulikov and Kulikova (2015b) | Cited by | Penas et al. (2017) | Cited by |
| Kulikov and Kulikova (2015a) | Cited by | Plesa et al. (2017) | Cited by |
| Kulikov and Kulikova (2017) | Cited by | Poovathingal and Gunawan (2010) | Cited by |
| Kurt et al. (2016) | Cited by | Pullen and Morris (2014) | Cited by |
| Kutalik et al. (2007) | Cited by | Quach et al. (2007) | Cited by |
| Kuwahara et al. (2013) | Cited by | Radulescu et al. (2012) | Cited by |
| Lakatos et al. (2015) | Cited by | Rakhshania et al. (2016) | Cited by |
| Lang and Stelling (2016) | Cited by | J. O. Ramsay and Cao (2007) | Cited by |
| Li and Vu (2013) | Cited by | Rapaport and Dochain (2005) | Cited by |
| Li and Vu (2015) | Cited by | Reinker et al. (2006) | Cited by |
| Liao et al. (2015a) | Cited by | Reis et al. (2018) | Cited by |
| Liao et al. (2015b) | Cited by | Remlia et al. (2017) | Cited by |
| Liepe et al. (2014) | Cited by | Rempala (2012) | Cited by |
| Lillacci and Khammash (2010a) | Cited by | Rosati et al. (2018) | Cited by |
| Lillacci and Khammash (2010b) | Cited by | Ruess et al. (2011) | Cited by |
| Lillacci and Khammash (2012) | Cited by | Ruess (2014) | Cited by |
| Lindera and Rempala (2015) | Cited by | Ruess and Lygeros (2015) | Cited by |
| Liu et al. (2006) | Cited by | Rumschinski et al. (2010) | Cited by |
| Liu and Wang (2008b) | Cited by | Ruttor and Opper (2009) | Cited by |
| Liu and Wang (2008a) | Cited by | Sagar et al. (2017) | Cited by |
| Liu and Wang (2009) | Cited by | Saltelli et al. (2004) | Cited by |
| Liu et al. (2012) | Cited by | Saltelli et al. (2005) | Cited by |
| Liu and Gunawan (2014) | Cited by | Schilling et al. (2016) | Cited by |
| Loos et al. (2016) | Cited by | Schnoerr et al. (2017) | Cited by |
| Lück and Wolf (2016) | Cited by | Septier and Peters (2016) | Cited by |
| Mancini et al. (2015) | Cited by | Shacham and Brauner (2014) | Cited by |
| Mannakee et al. (2016) | Cited by | Sherlock et al. (2014) | Cited by |
| Mansouri et al. (2014) | Cited by | Shiang (2009) | Cited by |
| Mansouri et al. (2015) | Cited by | Zamora-Sillero et al. (2011) | Cited by |
| Matsubara et al. (2006) | Cited by | Singh and Hahn (2005) | Cited by |
| Mazur (2012) | Cited by | Slezak et al. (2010) | Cited by |
| Mazur and Kaderali (2013) | Cited by | Smet and Marchal (2010) | Cited by |
| McGoff et al. (2015) | Cited by | Smith and Grima (2018) | Cited by |
| Mendes and Kell (1998) | Cited by | He et al. (2004) | Cited by |
| Meskin et al. (2011) | Cited by | Srinath and Gunawan (2010) | Cited by |
| Meskin et al. (2013) | Cited by | Srinivas and Rangaiah (2007) | Cited by |
| Meyer et al. (2014) | Cited by | Srivastavaa and Rawlingsb (2014) | Cited by |
| Michailidis and d'Alché Buc (2013) | Cited by | von Stosch et al. (2014) | Cited by |
| Michalik et al. (2009) | Cited by | Emmert-Streib et al. (2012) | Cited by |
| Mihaylova et al. (2011) | Cited by | Sun et al. (2008) | Cited by |
| Mihaylova et al. (2012) | Cited by | Sun et al. (2012) | Cited by |
| Mihaylova et al. (2014) | Cited by | Sun et al. (2014) | Cited by |
| Mikeev and Wolf (2012) | Cited by | Tangherloni et al. (2016) | Cited by |
| Milner et al. (2013) | Cited by | Teijeiro et al. (2017) | Cited by |
| Mizera et al. (2014) | Cited by | Tenazinha and Vinga (2011) | Cited by |
| Moles et al. (2003) | Cited by | Thomas et al. (2012) | Cited by |
| Jaime and Denis (2015) | Cited by | Tian et al. (2007) | Cited by |
| Mu (2010) | Cited by | Tian et al. (2010) | Cited by |
| Müller et al. (2012) | Cited by | Toni and Stumpf (2010) | Cited by |
| Murakami (2014) | Cited by | Transtrum and Qiu (2012) | Cited by |
| Nemeth et al. (2014) | Cited by | Vanlier et al. (2013) | Cited by |
| Nienaltowski et al. (2015) | Cited by | Vargas et al. (2014) | Cited by |
| Nim et al. (2013) | Cited by | Villaverde et al. (2012) | Cited by |
| Nobile et al. (2012) | Cited by | Villaverde et al. (2014) | Cited by |
| Nobile et al. (2013) | Cited by | Villaverde and Barreiro (2016) | Cited by |
| Nobile et al. (2015) | Cited by | Villaverde et al. (2016) | Cited by |
| Nobile et al. (2016) | Cited by | Voit (2013) | Cited by |
| Nobile et al. (2018a) | Cited by | Vrettas et al. (2011) | Cited by |
| Pahle et al. (2012) | Cited by | Wang et al. (2010) | Cited by |
| Pan and Yang (2010) | Cited by | Weber and Frey (2017) | Cited by |

**Table S3.** The references with citation links in Google Scholar. (cont.)

| | | | |
|---|---|---|---|
| Weiss et al. (2016) | Cited by | Zechner et al. (2012) | Cited by |
| Whitaker et al. (2017) | Cited by | Zeng et al. (2012) | Cited by |
| White et al. (2015) | Cited by | Zhan and Yeung (2011) | Cited by |
| Wong et al. (2015) | Cited by | Zhan et al. (2014) | Cited by |
| Woodcock et al. (2011) | Cited by | Zimmer et al. (2014) | Cited by |
| Xiong and Zhou (2013) | Cited by | Zimmer and Sahle (2012) | Cited by |
| Yang et al. (2014) | Cited by | Zimmer and Sahle (2015) | Cited by |
| Yang et al. (2012) | Cited by | Zimmer (2015) | Cited by |
| Yenkie et al. (2016) | Cited by | Zimmer et al. (2016) | Cited by |
| Zechner et al. (2011) | Cited by | Zimmer (2016) | Cited by |
